# Achieving Near‐Complete Dechlorination of Poly(vinyl chloride) via Electrochemical Reduction

**DOI:** 10.1002/cssc.70715

**Published:** 2026-05-18

**Authors:** Rahul Kant Jha, Henry E. Thurber, Bertrand J. Neyhouse, Quinn L. VanZile, Anne J. McNeil

**Affiliations:** ^1^ Department of Chemistry University of Michigan Ann Arbor Michigan USA; ^2^ Macromolecular Science and Engineering Program University of Michigan Ann Arbor Michigan USA

**Keywords:** chemical recycling, dechlorination, electrochemistry, plasticizers, poly(vinyl chloride)

## Abstract

Despite its ubiquity, scalable recycling of poly(vinyl chloride) (PVC) remains elusive. One challenge is that the waste streams can be contaminated with plasticizers. Herein, we present a strategy that repurposes these plasticizers as redox mediators in a controlled‐voltage electrolysis to dechlorinate PVC. Five common plasticizers were evaluated as redox mediators. In addition, using voltage limits via a galvanostatic cycling with potential limitation protocol attenuated deleterious mediator decomposition. Among the plasticizers screened, trioctyl trimellitate enabled up to 97% dechlorination of PVC across a range of PVC samples, including commercial‐grade rigid pipe and flexible tubing, and scales (up to 0.5 g), illustrating broad applicability. This method achieves near‐quantitative PVC dechlorination in a simple, one‐pot transformation while simultaneously utilizing the liberated chloride, offering a promising solution for transforming waste PVC into recycled products.

## Graphical abstract

A one‐pot controlled‐voltage electrolysis achieved up to 97% dechlorination of poly(vinyl chloride) using common plasticizers as redox mediators. The liberated chloride ions were oxidized at the anode and utilized in a subsequent arene chlorination, offering a route for repurposing the chlorine from PVC. The protocol was successful with diverse PVC samples, from rigid pipe to flexible tubing, and at scale (0.5 g).

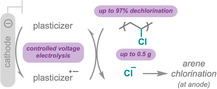

## 1 Introduction

Poly(vinyl chloride) (PVC) is the third‐largest‐volume polymer produced globally, owing to its exceptional durability, versatility, flexibility, and chemical stability [1]. Despite these advantages, additives and plasticizers that provide the desired properties in flexible PVC plastics [2, 3] often lead to cross‐contamination within the plastic waste stream, complicating waste sorting and disrupting recycling efficacy at scale [4, 5]. Furthermore, because thermal treatment of PVC generates corrosive hydrochloric acid and other volatile by‐products, thermochemical recycling processes, such as pyrolysis and combustion, pose safety risks and increase reactor corrosion [6]. Consequently, of an estimated 700,000 tons of post‐consumer PVC discarded annually in the United States, ca. 3% is recycled, while the remainder is primarily landfilled (88%) or incinerated (9%), leaving ca. 380,000 tons of chlorine content unutilized [7]. As such, alternative recycling technologies must be developed to facilitate end‐of‐life management for PVC plastics [8, 9, 10].

Chemical functionalization of PVC presents a promising platform for transforming waste into value‐added products [8]. Hydrothermal treatment under strongly basic conditions (pH > 14) and elevated temperatures (>200°C) produces insoluble carbonaceous char and chlorinated products like hydrochloric acid or sodium chloride [11]. Alternatively, milder, base‐mediated methods have been reported for PVC dehydrochlorination to generate polyene‐like polymers [12, 13, 14, 15, 16]. Recent efforts from Fieser et al. and De Vos et al. have focused on silylium‐mediated [17, 18, 19] and metal‐catalyzed [20, 21, 22] methods to replace chlorine and generate functional polymers. Despite the elegance of these approaches, some practical challenges remain. For example, additives present in commercial PVC products can interfere with reaction outcomes or necessitate additional processing steps to remove them [23]. In contrast, if additives inherently present in PVC products, such as plasticizers, could be used directly as reagents to repurpose PVC, a more streamlined approach to converting waste into useful products could be achieved.

To this end, we had previously reported an electrochemical method to reductively dechlorinate PVC—mediated by the phthalate plasticizer di(2‐ethylhexyl)phthalate (DEHP)—and utilized the chlorine atoms in a tandem electrooxidative chlorination to produce valuable chlorinated aromatics [24, 25]. Compared to conventional pyrolysis of PVC—which typically operates at elevated temperatures (>400°C), consumes substantial energy, generates undesired chlorine contamination of pyrolytic oils, and releases hazardous by‐products such as HCl and polycyclic aromatic hydrocarbons [6, 26, 27]—our electrochemical repurposing offers a more sustainable alternative by proceeding at room temperature and utilizing the chlorine in a productive process, thus lowering its environmental impact [28, 29, 30, 31]. While promising, this method faced key challenges. By operating under constant‐current electrolysis, the voltage was not controlled, resulting in progressively higher voltages as dechlorinated PVC (dPVC) precipitated, fouling the electrodes and increasing resistance. This elevated voltage also triggered decomposition of the solvent (*N*, *N*‐dimethylformamide (DMF)), the supporting electrolyte, and the mediator. Consequently, an excess of PVC (8 equiv) was required to chlorinate an aromatic substrate (1 equiv). Combined, the excess reagents and parasitic reactions limited PVC dechlorination to ca. 20%. Recently, Besenius and Waldvogel advanced this plasticizer‐mediated PVC dechlorination via the same constant‐current electrolysis [32]. They achieved up to 94% dechlorination, but it required four sequential electrolysis cycles, involving extraction and redissolution of the partially dechlorinated PVC—significantly increasing process complexity and leading to poor charge utilization.

We hypothesized that conducting a constant‐current electrolysis with a defined voltage limit could minimize these undesired side reactions and optimize energy consumption. Herein, we describe a controlled‐voltage method for plasticizer‐mediated dechlorination of PVC that restricts voltage progression during electrolysis and minimizes unwanted decomposition. Under these new conditions, up to 90% PVC dechlorination was observed, and DEHP degradation was reduced by 50%. Next, we assessed whether other plasticizers could act as redox mediators for PVC dechlorination, as DEHP and similar plasticizers (e.g., butyl benzyl phthalate (BBP), diisononyl phthalate (DINP)) have been largely phased out due to their substantial health and environmental risks [33, 34, 35, 36]. Alternative, but structurally analogous plasticizers, including dioctyl terephthalate (DOTP) and trioctyl trimellitate (TOTM), have become more prevalent in PVC plastics. Notably, TOTM achieved up to 97% dechlorination in a simple, one‐pot process when applied to commercial PVC plastics, including rigid pipe and flexible tubing, and on a larger scale (0.5 g). While this process productively used the generated chloride ions to produce a chlorinated aromatic, the resulting hydrocarbon content (dPVC) was insoluble. Overall, this work demonstrates that plasticizers, a significant barrier in most recycling approaches, can serve as beneficial redox mediators, enabling near‐complete PVC dechlorination.

## 2 Results and Discussion

### 2.1 Screening Plasticizers as Potential Redox Mediators

**FIGURE 1:**
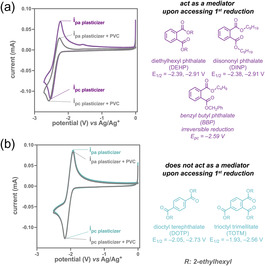
(a) Plasticizers that act as a mediator in their first reduction with a representative cyclic voltammogram of DEHP with and without PVC at a scan rate of 100 mV/s. (b) Plasticizers with no mediator behavior in their first reduction with a representative cyclic voltammogram of DOTP with and without PVC at a scan rate of 100 mV/s. Reduction potentials (*E*_1/2_) are referenced to Ag/Ag^+^. Due to BBP's irreversibility, the reduction at the peak cathodic current (*E*_pc_) is reported. Concentrations include PVC_37k_ (30 mM), plasticizers (10 mM), and NBu_4_BF_4_ (0.1 M) in DMF, whereas *i*_pa_ and *i*_pc_ refer to the anodic and cathodic peak currents, respectively.

We began by conducting cyclic voltammetry (CV) on five commercial plasticizers (e.g., DEHP, DINP, BBP, DOTP, and TOTM), both with and without PVC present (Figures S21–S32, Tables S9–S13). As shown in Figure 1a, redox mediation can be inferred when the reversibility of the reduction peak is diminished upon adding PVC, indicating that electron transfer occurred between the reduced plasticizer and PVC [24]. Conversely, if a plasticizer is not a mediator, its CV response remains unchanged upon PVC addition, as no electron transfer is happening (Figure 1b). All five plasticizers demonstrated a reversible first‐reduction potential (*i*_pa_/*i*_pc_ ≈ 1) without PVC except for BBP, which likely undergoes benzyl radical release upon reduction (Figure S26) [37]. With PVC present, both DEHP (*E*_1/2_ = –2.39 V vs Ag/Ag^+^) and DINP (*E*_1/2_ = –2.38 V vs Ag/Ag^+^) acted as mediators, whereas DOTP (*E*_1/2_ = –2.05 V vs Ag/Ag^+^) and TOTM (*E*_1/2_ = –1.93 V vs Ag/Ag^+^) did not. The PVC concentration was then doubled to assess whether the biomolecular electron transfer reaction with DOTP and TOTM could be promoted [38]. However, no mediation was observed at the first reduction peak (Figures S29 and S32). Taken together, these results suggest a strong correlation between reduction potential and redox mediation: that is, only mediators with a reduction potential more negative than –2.05 V versus Ag/Ag^+^ were successful. Indeed, when DOTP and TOTM were pushed to their quasi‐reversible second reduction potentials (–2.73 and –2.56 V vs Ag/Ag^+^, respectively), they exhibited mediating behavior (Figures S28 and S31). However, an additional peak appeared around –0.6 V upon reoxidation, likely due to the partial degradation of the reduced aromatic esters [37, 39]. To explore if this chemistry extends to chloroalkanes, we performed CV using 2‐chloroheptane as a small‐molecule mimic of PVC (Figure S23). No mediation was observed with DEHP, suggesting that PVC has a more facile reduction due to the proximate chlorines and structural defects [40, 41, 42].

### 2.2 Optimizing Conditions to Maximize Dechlorination

Our previously reported method was carried out in an Electrasyn, which had limited control over operating voltages during electrolysis. We hypothesized that setting a voltage cutoff could limit undesired side reactions (e.g., mediator, solvent, and supporting electrolyte degradation). To test this hypothesis, our electrolyses herein were carried out under constant‐current conditions in a glovebox using a custom‐designed H‐cell (Figures S15 and S16) equipped with an Ag/Ag^+^ reference electrode to monitor and control the electrode potential. Reticulated vitreous carbon (RVC) electrodes were selected for their high surface area and low cost. The cathode (working electrode) and anode (counter electrode) compartments were separated by a glass frit to prevent undesired reactions at both electrodes. Phenetole was used as the substrate for anodic chlorination [24].

**FIGURE 2:**
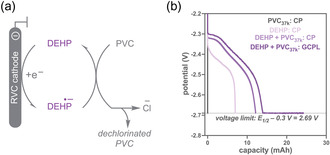
(a) DEHP‐mediated electrochemical dechlorination of PVC; anodic reaction is omitted for clarity. (b) Plot of potential versus capacity for CP and GCPL electrolysis. PVC_37k_ (0.2 M) and/or DEHP (0.05 M), NBu_4_BF_4_ (0.3 M), DMF (5 mL) in working side; phenetole (0.2 M, 0.05 M without PVC), NBu_4_BF_4_ (0.3 M), DMF (5 mL) in counter side. CP/GCPL electrolysis, –8 mA, voltage limit: –2.69 V versus Ag/Ag^+^. CP = Chronopotentiometry; GCPL = galvanostatic cycling with potential limitation.

In this setup, we performed chronopotentiometry (CP) by applying a constant current with a voltage limit set to 300 mV more negative than the mediator's *E*_1/2_ (SI Section II.G, method 1). The additional 300 mV of overpotential, relative to the formal redox potential (*E*_1/2_), was applied to drive mediator reduction to near‐complete conversion [43]. Upon reaching the voltage limit, the potentiostat was automatically turned off, preventing further voltage progression that could trigger undesired side reactions. As a baseline, the constant‐current electrolysis of DEHP alone yielded a capacity of 6.8 mAh, closely matching the theoretical capacity (6.7  mAh, SI Section II.F for calculation; Figure 2b). Conducting the same experiment with both DEHP (0.25 equiv) and PVC_37k_ (1 equiv) resulted in a marked capacity increase due to the electron transfer from reduced DEHP to PVC (13 mAh, 40% of the theoretical 33.4 mAh) (Figure 2b). Post‐electrolysis analysis indicated that only 16% of DEHP was consumed, as measured by ^1^H NMR spectroscopy (SI Section II.B for calculation). This DEHP loss is substantially lower than the ≈99% we observed previously without voltage control [24]. Using combustion ion chromatography (CIC), ≈40% PVC dechlorination was achieved, based on the residual chlorine content (Table S15). In contrast, the same electrolysis conducted with PVC_37k_ alone (no DEHP) reached the voltage limit immediately without generating any capacity (Figure 2b), confirming that DEHP is necessary for PVC reduction under these conditions.

One strategy to further increase the dechlorination yield is to extend the reaction time without exceeding the voltage threshold. To this end, we leveraged galvanostatic cycling with potential limitation (GCPL) electrolysis (SI Section II.G, method 2). In the GCPL process, the reaction initially proceeds at a constant current until the voltage limit is reached, at which point the system maintains that voltage. The current will then slowly decrease over time. The total reaction time was set to 7 h (≈2 h at constant current and the remaining 5 h at the potential limit) after which electrolysis was manually stopped because the current was ≈|1.5 mA|. This threshold was chosen because at this low current, we anticipate minimal additional capacity while potentially increasing undesired side reactions (Figure S34) [44]. Excitingly, a capacity of ≈23 mAh (70% of the theoretical capacity) was achieved under GCPL conditions with DEHP and PVC_37k_ (Figure 2b). Post‐electrolysis analysis revealed a 50% loss of DEHP and a remarkable 80% PVC dechlorination, reflecting a fourfold improvement over our previous work and a twofold increase compared to the CP electrolysis discussed above (Table S15).

Despite significant PVC dechlorination, the yield of chlorinated phenetole remained surprisingly low (<2%) after the GCPL electrolysis. We hypothesized that this low yield reflected sluggish chloride ion migration from the cathode compartment through the frit to the anode. More specifically, we surmised that the supporting electrolyte's relatively high concentration (0.3 M NBu_4_BF_4_) led to preferential migration of BF_4_^–^ ions over Cl^–^ ([0.16 M] at 80% PVC dechlorination). As a result, the anode lacked sufficient chloride to drive phenetole chlorination. To overcome this challenge, we replaced NBu_4_BF_4_ with NBu_4_Cl as the supporting electrolyte. Here, chloride ions come from both the NBu_4_Cl itself and the PVC dechlorination. This modification significantly improved the phenetole chlorination yield to 41% (SI Section II.C). Considering that chlorination is a 2‐electron process, the obtained 41% yield translates to a ≈100% Faradaic yield (SI Section II.D) (Table 1, entry 1).

**TABLE 1:** Optimization of PVC dechlorination as a function of DEHP concentration^a^.

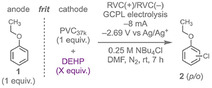						
Entry	DEHP, equiv	Capacity, mAh	PVC dechlorination, %	Loss of DEHP, %	Yield of 2, % (by GC–MS)	Yield of 2, % (Faradaic yield)
1	0.25	22 ± 1	80 ± 3	55 ± 14	41 ± 1	100 ± 8
2	0.50	27 ± 1	88 ± 2	51 ± 9	50 ± 4	100 ± 4
3	0.70	26 ± 2	89 ± 1	37 ± 16	43 ± 4	88.3 ± 0.4
4	0	0.45	<1	na^b^	nd^c^	na^b^

a Cathode and anode compartments each contain 5 mL solution.

b Not applicable.

c Not detected.

Next, we varied the relative DEHP concentration to increase the frequency of productive collisions between the reduced DEHP radical anion and PVC. Indeed, a higher DEHP concentration (0.5 equiv) gave both a higher capacity (80% of theoretical capacity) (Table 1, entry 2) and higher PVC dechlorination (88%). Phenetole chlorination was also increased to ca. 50% yield. Potentiometric titration of the electrolysis solutions with AgNO_3_ confirmed a substantial increase in solution chloride ion concentration [45], corresponding to 86% PVC dechlorination when accounting for the chloride consumed in the reaction (SI Section V.A). Further increasing the DEHP concentration (0.7 equiv) yielded a comparable capacity, with marginally higher dechlorination (89% by CIC) (Table 1, entry 3). As a result, 0.5 equiv was selected for all subsequent reactions to balance plasticizer loading and dechlorination yields. As expected, a control experiment without DEHP instantaneously hit the voltage limit, yielding no appreciable dechlorination (Table 1, entry 4). Because chloride ions from NBu_4_Cl are present at the anode, running the electrolysis with DEHP alone resulted in phenetole chlorination comparable to that of the DEHP/PVC system (Table S19). In this case, however, the chloride ions get depleted, whereas the chloride ions get replenished when PVC was present.

### 2.3 Understanding the Loss of DEHP

**FIGURE 3:**
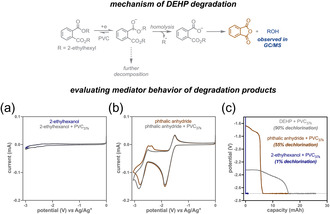
Proposed decomposition pathway of DEHP. Cyclic voltammograms of (a) 2‐ethylhexanol and (b) phthalic anhydride with PVC_37k_ to assess their mediated behavior. (c) Plot of potential versus capacity during GCPL with DEHP, phthalic anhydride, and 2‐ethylhexanol (independently) with PVC_37k_ under standard electrolysis conditions: PVC_37k_ (0.2 M), DEHP (0.1 M), phthalic anhydride/2‐ethylhexanol (0.05 M), NBu_4_Cl (0.25 M) in DMF (5 mL) in working side; phenetole (0.2 M), NBu_4_Cl (0.25 M) in DMF (5 mL) in counter side. GCPL, –8  mA, voltage limit: –2.69 V (vs Ag/Ag^+^) for 7 h.

To identify the DEHP decomposition pathways responsible for its loss during electrolysis, we quantified its (i) migration through the frit to the anode chamber, (ii) decomposition from the radical anion, and (iii) reaction with macroradicals on the polymer backbone. To measure migration, the solutions were analyzed by ^1^H NMR spectroscopy before and after electrolysis; these data showed that ≈12% of the DEHP had migrated (due to a concentration imbalance) through the frit from the cathode to the anode (Table S21). To measure DEHP decomposition, similar analyses were performed on solutions following DEHP electrolysis without PVC; these studies revealed a 70% loss stemming from competitive side reactions of the radical anion (e.g., anionic dimerization, oxygen–alkyl homolysis) [37]. The oxygen–alkyl homolysis pathway, which would generate a monoester carboxylate anion, may further react to generate phthalic anhydride and 2‐ethylhexanol (observed in a mass spectrum, Figure S38) (Figure 3 top). Notably, these decomposition pathways were attenuated when PVC was present (≈50% DEHP loss) due to the electron transfer to PVC. To determine the reactivity of DEHP (and its decomposition products) with the macroradical, we again used NMR spectroscopy. Specifically, a shorter electrolysis (2 h) was run to isolate a soluble dechlorinated PVC (dPVC–20%) (SI Section VI.C; Table S23). Then, diffusion‐ordered spectroscopy showed aromatic, alkene, and aliphatic substitution on the polymer backbone (Figure S40), consistent with radical–radical reactions, as well as substitution and elimination. Size‐exclusion chromatography showed that dPVC–20% has a slightly lower *M*_*n*_ (36,000 g/mol) and higher dispersity compared to PVC_37k_ (2.54 vs 1.86) (Table S24). The lower number‐average molar mass may be due to loss of chlorine and/or some radical‐induced chain scission [18], while the increased dispersity may result from radical coupling of two macroradicals. Combined, these studies reveal multiple pathways for DEHP to undergo losses during the electrolyses.

We also wondered whether the DEHP decomposition products contribute to PVC dechlorination under the electrolysis conditions. To evaluate this hypothesis, cyclic voltammograms of both 2‐ethylhexanol and phthalic anhydride were run with and without PVC (Figure 3a,b). While no reduction peak was observed for 2‐ethylhexanol, phthalic anhydride exhibited two quasireversible reduction waves at –1.65 and –2.65 V versus Ag/Ag^+^, with the second reduction potential showing modest mediated behavior with PVC. Next, GCPL electrolysis of PVC (1 equiv) and phthalic anhydride (0.25 equiv) was performed with the same voltage limit previously used with DEHP to mimic the authentic reaction conditions (Figure 3c). The rationale for 0.25 equiv is that it represents a 50% loss of DEHP (0.5 equiv), similar to what we saw above. Under these conditions, we observed 55% PVC dechlorination with complete loss of the phthalic anhydride (Table S22; Figure S35). In contrast, no appreciable dechlorination (≈1%) was observed upon GCPL electrolysis of 2‐ethylhexanol with PVC_37k_, confirming its nonreactivity under these conditions (Table S22). Although 2‐ethylhexanol was not recovered postelectrolysis, its retrieval remains feasible and could be efficiently leveraged for synthesizing PVC plasticizers (such as DEHP) and acrylates for coatings and adhesives [46]. Together, these results show that DEHP and at least one of its decomposition products can dechlorinate PVC; other unidentified DEHP decomposition products may also facilitate this reaction.

### 2.4 Applying Optimized Conditions to Other Plasticizers

**FIGURE 4:**
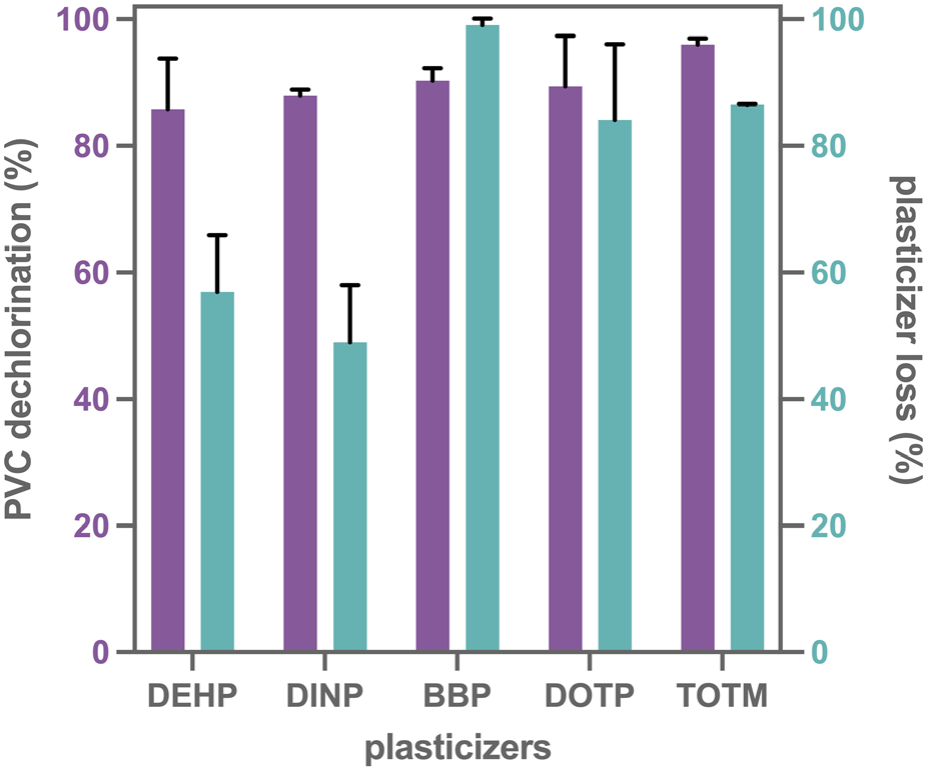
Plot of dechlorination efficiency (and plasticizer loss) versus plasticizer identity when electrolyses were run under GCPL conditions: PVC_37k_ (0.2 M), plasticizer (0.1 M), NBu_4_Cl (0.25 M) in DMF (5 mL) in working side; phenetole (0.2 M), NBu_4_Cl (0.25 M) in DMF (5 mL) in counter side. GCPL, –8 mA, voltage limit: –2.8 V (vs Ag/Ag^+^) for 7 h.

With optimized GCPL electrolysis conditions in hand, we evaluated four other plasticizers: DINP, BBP, DOTP, and TOTM. Recall that the cyclic voltammograms showed that all these plasticizers can act as mediators for PVC dechlorination at their first or second reduction potentials (Figures S21–S32). Therefore, the voltage limit was set to –2.8 V to access both these reduction potentials. Notably, under these conditions, all tested plasticizers (and/or their decomposition products) acted as mediators, resulting in significant PVC dechlorination (Figures 4 and S42). Among all tested plasticizers, TOTM achieved the highest PVC dechlorination, reaching up to 96% by CIC. This higher dechlorination may be due to more efficient electron transfer to PVC and/or the formation of redox‐active decomposition products. To support the CIC results, the insoluble dPVC extracted from the TOTM electrolysis was analyzed by thermogravimetric analysis, which showed significantly less HCl evolution than that of native PVC, consistent with high dechlorination (Figure S44). A solid‐state Fourier transform infrared spectrum showed a much smaller C–Cl stretch than in PVC (Figure S45). Additional spectral features, including enhanced stretching at ≈1600 cm^−1^ and shifting of C—H stretching above 3000 cm^−1^, suggest alkene formation and a polyacetylene‐like backbone, likely accounting for its insolubility. The nearly dechlorinated, hydrocarbon‐rich dPVC may be useful as activated carbon or filler material, among others [47], thereby enhancing overall process sustainability through valorization of both the recovered chlorine and the remaining polymer. Given its superior dechlorination efficiency, TOTM was selected for all subsequent studies. In comparison to our earlier work [24], where 160 mAh capacity was accessed to achieve 20% dechlorination, this improved protocol enabled 96% dechlorination with ≈45 mAh—a 17‐fold improvement in dechlorination efficiency per unit charge, demonstrating a significant enhancement in energy utilization. However, the insoluble dPVC is still not utilized, lowering the overall atom economy.

### 2.5 Expanding the Scope and Scale

Because a soluble molecule mediates these electrochemical reactions, we hypothesized that PVC itself need not be soluble. We tested this hypothesis by employing high‐molar‐mass PVC (*M*_*n*_: 122,000 g/mol), which swells but does not fully dissolve in DMF at room temperature. Indeed, an 84% dechlorination of PVC was observed with TOTM (Table S25), underscoring the utility of this approach. The slightly lower yield (compared to PVC_37k_) may be due to slower diffusion of redox mediator into the swollen polymer matrix [48, 49], which is a prerequisite for electron transfer.

A rigid PVC pipe and flexible vinyl tubing were selected as representative examples of unplasticized and plasticized PVC products, respectively. To determine the molar mass of PVC in each sample, the PVC was extracted by dissolution in tetrahydrofuran, followed by precipitation in methanol and analysis via size‐exclusion chromatography (SI Sections VIII.A and VIII.B, PVC_pipe_ (*M*_*n*_ = 102,000 g/mol) and PVC_tubing_ (*M*_*n*_ = 77,000 g/mol)). Gas chromatography coupled with mass spectrometry was used to verify the identity of the plasticizer in the tubing (DEHP, 28% by weight) (Figure S46) [24].

**FIGURE 5:**
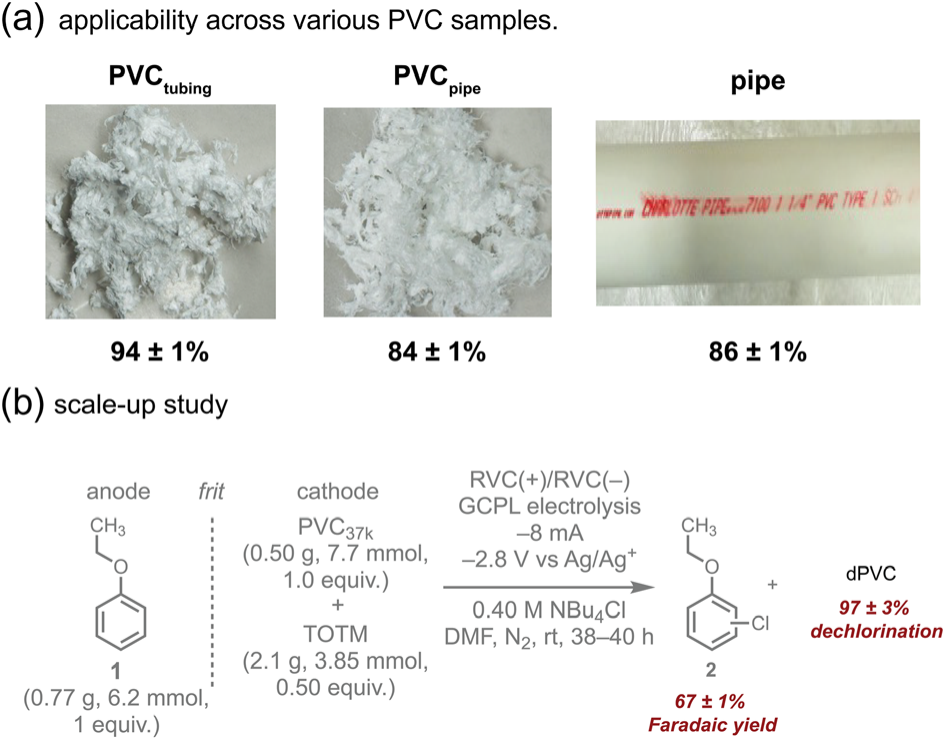
(a) Dechlorination efficiency with various PVC samples; electrolyses were performed with TOTM under standard conditions: PVC source (0.2 M), TOTM (0.1 M), NBu_4_Cl (0.25 M) in DMF (5 mL) in working side; phenetole (0.2 M), NBu_4_Cl (0.25 M) in DMF (5 mL) in counter side. GCPL, –8 mA, voltage limit: –2.8 V (vs Ag/Ag^+^) for 7 h. (b) Scale‐up demonstrating 97% PVC dechlorination.

The rigid pipe was fragmented into small pieces and then stirred in DMF (containing 0.25 M NBu_4_Cl) for 60 min at 50°C to aid dissolution. The resultant heterogeneous mixture appeared as a white suspension, likely due to inorganic fillers and pigments. Electrolysis of this heterogeneous mixture with added TOTM resulted in a remarkable 84% dechlorination (Figure 5a, Table S29), indicating that the method is largely unaffected by these fillers and additives. As a comparison, electrolysis of the PVC extracted from the pipe with added TOTM showed a comparable dechlorination yield of 86% (Figure 5a, Table S29). While separating additives was not necessary to achieve high dechlorination, doing so could simplify downstream separation and enable reuse of these compounds.

For the flexible tubing, which already contained DEHP as a plasticizer, electrolysis was performed without adding TOTM (SI Section VIII, Electrolysis Procedure F). The tubing was first fully dissolved in DMF (containing 0.25 M NBu_4_Cl) by stirring for 30 min at 50°C. Subsequent electrolysis of this solution resulted in 16% PVC dechlorination (Table S29), consistent with the anticipated yield based on the amount of DEHP present. For comparison, electrolysis of extracted PVC from the tubing with added TOTM resulted in 94% dechlorination (Figure 5a; Table S29).

Given the consistently high dechlorination yields observed with TOTM across multiple PVC sources, we decided to try a larger‐scale GCPL electrolysis. Specifically, we used 0.5 g of PVC_37k_ under the optimized conditions (Figure 5b; SI Section IX, Electrolysis Procedure I). This approach achieved an impressive 97% PVC dechlorination (Table S32). While even larger‐scale reactions need to be explored, the near‐complete dechlorination observed here highlights the potential of this electrochemical strategy for commercial PVC recycling.

## 3 Conclusion

Repurposing waste plastics into alternative products is vital for effective waste management and the plastics ecosystem overall. Our work herein demonstrated that a controlled‐voltage electrolysis strategy can achieve near‐complete dechlorination of PVC. This method is mediated by commonly used plasticizers already present in many PVC plastics. Among the tested plasticizers, trioctyl trimellitate (TOTM) exhibited the highest efficiency, achieving up to 97% dechlorination. We demonstrated broad applicability across different PVC samples, including an insoluble (but swellable) high molar‐mass PVC and real‐world PVC products (rigid pipe and flexible tubing). A scale‐up demonstration with PVC_37k_ and TOTM under the optimized electrolysis conditions achieved 97% dechlorination, demonstrating the promise for larger‐scale implementation. While chloride ions generated during PVC dechlorination were productively utilized for the anodic chlorination of phenetole, the resulting hydrocarbon content (dPVC) remained insoluble. Future work aims to harness the polymer macroradicals for functionalization, thereby enabling the conversion of PVC into multiple valuable products. In addition, we aim to expand the anodic chlorination to synthesize other chlorinated products, including chlorobenzene, 1,2‐dichloroethane, chlorophenol, and chlorotoluene, that serve as key intermediates for industrial applications. Moreover, identifying a more benign solvent capable of dissolving or swelling PVC to replace DMF—a toxic and environmentally hazardous solvent [50]—will further enhance the process sustainability.

## Supporting Information

Additional SI can be found online in the Supporting Information section.

## Funding

This work (and RKJ) was supported through a subcontract from the Ames Laboratory with funding from the U.S. Department of Energy (DOE), Office of Science, Office of Basic Energy Sciences, under Contract no. DE‐AC02–07CH11358 as part of the Energy Frontier Research Center titled the Institute for Cooperative Upcycling of Plastics (iCOUP). BJN was supported by The Vinyl Institute through its VIABILITY recycling grant program.

## Conflicts of Interest

The authors declare no conflicts of interest.

## Supporting information

Supplementary Material

## Data Availability

The processed data that support the findings of this study are available in the SI.
