# Supplementary material for: Achieving Near‐Complete Dechlorination of Poly(vinyl chloride) via Electrochemical Reduction
Source: ChemSusChem. 2026 May 18;19(10):e70715. doi: 10.1002/cssc.70715 (PMC13181707; doi:10.1002/cssc.70715)
Supplement: Supplementary file 1 — Supplementary Material [file CSSC-19-e70715-s001.pdf]

Supporting information for:

Achieving Near-Complete Dechlorination of Poly(vinyl chloride) via Electrochemical Reduction

Rahul Kant Jha,[a] Henry E. Thurber,[b] Bertrand J. Neyhouse,[a] Quinn L. VanZile,[a] and Anne J. McNeil[a,b]*

[a] *Department of Chemistry, University of Michigan, Ann Arbor, Michigan, 48109-1055, USA*
[b] *Macromolecular Science and Engineering Program, University of Michigan, Ann Arbor, Michigan, 48109-2102, USA*

Table of Contents

I. Materials 3

II. General experimental techniques 4

Nuclear Magnetic Resonance (NMR) Spectroscopy 4
Section II.A. ^{1}H NMR spectra for compounds used in this work 4
Section II.B. Representative mediator loss calculation via ^{1}H NMR spectroscopy 13

Gas Chromatography–Mass Spectrometry (GC-MS) 16
Section II.C. Representative calculation of phenetole chlorination by GC-MS 16
Section II.D. Representative calculation to determine Faradaic yield of phenetole chlorination 20

Size Exclusion Chromatography (SEC) 21

Fourier Transform Infrared (FTIR) Spectroscopy 22

Combustion Ion Chromatography (CIC) 22
Section II.E. Calculation of % dechlorination in dechlorinated PVC 23

Thermogravimetric Analysis (TGA) 23

Electrochemical Analysis 23
Cyclic Voltammetry (CV) 23
Bulk Electrolysis (BE) 24
Section II.F. Representative example to calculate theoretical capacity 26
Section II.G. Demonstration of electrolyses parameters used in this study 26

III. Screening mediated behavior by cyclic voltammetry 30

IV. CP and GCPL electrolysis with NBu_4BF_4 as supporting salt 40

Section IV.A. Procedure for the isolation of dPVC after electrolysis 41

V. GCPL electrolysis with NBu_4Cl as supporting salt 44

Section V.A. Potentiometric titration to determine dechlorination of PVC 48

VI. Understanding DEHP loss 51

Section VI.A. Crossover studies to determine loss of DEHP 51

Section VI.B. GCPL electrolysis of PVC_{37k} with phthalic anhydride or 2-ethylhexanol as a redox mediator 52

Section VI.C. GCPL electrolysis of PVC_{37k} with DEHP to determine incorporation of DEHP or its decomposition products onto dPVC backbone 55

VII. Applicability to other plasticizers 58

VIII. Electrolysis with real-world PVC plastics 63

Section VIII.A. Extraction of plasticizer from flexible tubing 63

Section VIII.B. Extraction of PVC from rigid pipe 64

IX. Scale-up studies with TOTM and PVC_{37k} 70

X. References 72

I. Materials

Poly(vinyl chloride) (PVC) was obtained from Sigma-Aldrich. Polymers were listed as M_w: ~43,000 g/mol (M_n: ~22,000 g/mol) and M_w: ~233,000 g/mol (M_n: ~99,000 g/mol). Size exclusion chromatography (SEC, *vide infra*) was performed to determine the molar mass of the purchased PVC. We will refer to these samples as PVC_{37k} (M_n: ~37,000 g/mol) and PVC_{122k} (M_n: ~122,000 g/mol) based on our analyses. Flexible tubing (Tygon® B-44-3) was purchased from McMaster-Carr and a rigid PVC pipe (Charlotte Pipe 7100) was obtained from a local hardware store.

Unless otherwise noted, these reagents were used as received.
From Sigma-Aldrich: di(2-ethylhexyl) phthalate (DEHP, analytical standard), benzyl butyl phthalate (BBP, 98%), dioctyl phthalate (DOTP, ≥96%), diisononyl phthalate (DINP, technical grade, ≥99%), tridecane (≥99%), phenetole (99%), 2-ethylhexanol (≥99.6%), tributylamine (for synthesis), silver tetrafluoroborate ($AgBF_4$, >99.99%, trace metal basis). *N,N*-Dimethylformamide (DMF, anhydrous, 99.8%) and acetonitrile (ACN, anhydrous 99.8%) were used after drying over molecular sieves. Tetrabutylammonium tetrafluoroborate (NBu_4BF_4, electrochemical grade, >99%) was used after drying at 100 ºC under high vacuum for 48 h.
From TCI America: tris(2-ethylhexyl) trimellitate (TOTM, >97%, GC), tetrabutylammonium chloride (NBu_4Cl, >98%).
From Fisher: toluene (ACS grade) and tetrahydrofuran (THF, HPLC grade, no preservatives). Molecular sieves (4 Å, 8–12 mesh) were activated by heating at 200 ºC under vacuum for ~12 h.
From Cambridge Isotope Laboratory: Tetrahydrofuran-d_8 (99.5%).

Ultrapure deionized (DI) water (≥ 18 MΩ) was obtained from a Millipore Synergy® water purification system. The glovebox in which specified procedures were carried out was an MBraun LABmaster 130 with an N_2 atmosphere (<0.5 ppm water and <10 ppm oxygen).

II. General experimental techniques

Nuclear Magnetic Resonance (NMR) Spectroscopy

^1H NMR spectra were acquired at room temperature on either a Varian VNMRS 500 or a Bruker Avance Neo 500. DOSY spectra were acquired in tetrahydrofuran-d_8 in a Varian VNMRS 500 operating at 500 MHz. Chemical shift data are reported in units (ppm) relative to tetramethylsilane (TMS) and referenced against residual solvent.

Section II.A. ^1H NMR spectra for compounds used in this work

Procedure for ^1H NMR experiment: ~10 mg of the compound was placed in a scintillation vial, and 0.6 mL of DMF was added using a pipette. The resulting solution was then transferred to an NMR tube. Because a non-deuterated solvent was used, manual gradient shimming was required. Shimming was performed by selecting the DMF peak at 8.03 ppm, using 4 scans with a 10 s delay and a pulse angle of 16.2 degrees. This process was completed in ~5 min. Following shimming, an ^1H NMR experiment was carried out at 500 MHz, using 16 scans with a 10 s delay and a pulse angle of 45 degrees. In some cases, poor shimming led to unusual peak splitting in DMF. The spectra were referenced to the DMF solvent peak at 8.03 ppm (singlet) prior to integrating the relevant compound peaks.

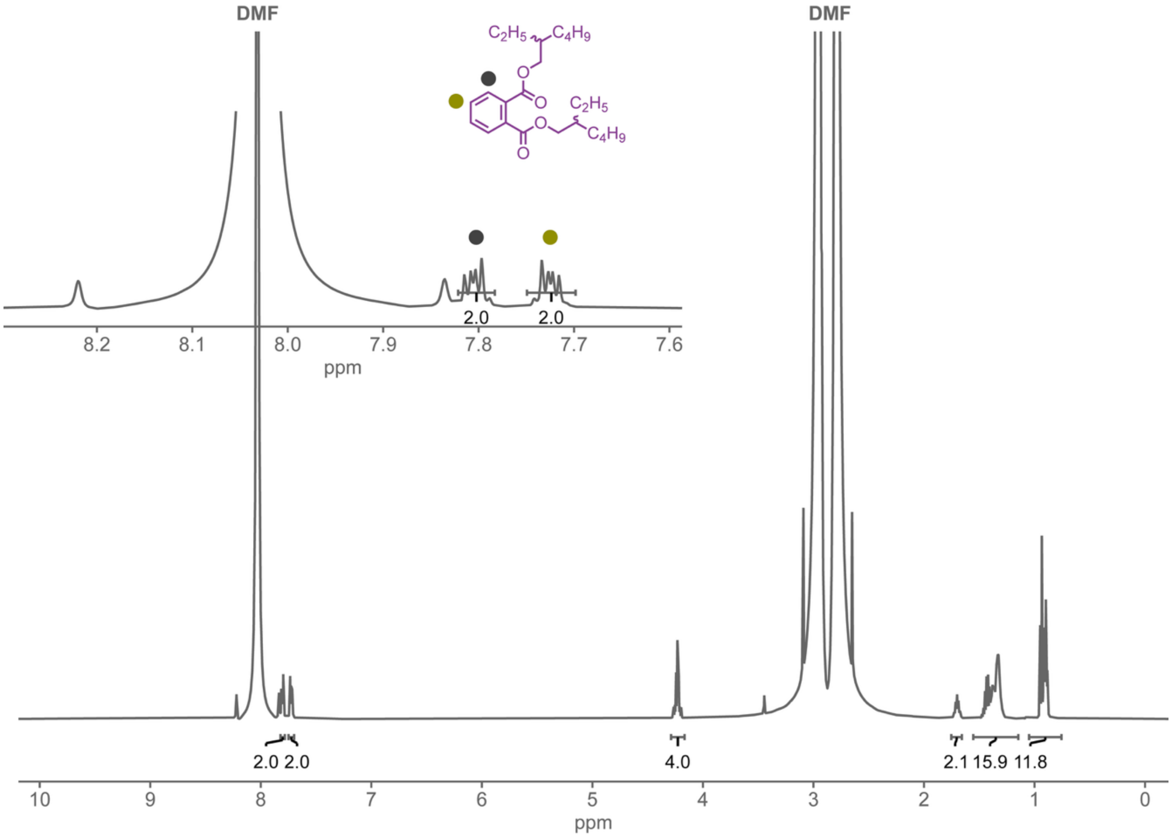

Figure S1. ^1H NMR spectrum of DEHP. ^1H NMR (500 MHz, DMF) δ 7.81 (m, 2H), 7.72 (m, 2H), 4.29 – 4.17 (m, 4H), 1.70 (m, 2H), 1.55 – 1.15 (m, 16H), 0.92 (m, 12H). The peak at 7.72 ppm was analyzed before and after electrolysis to determine the loss of DEHP.

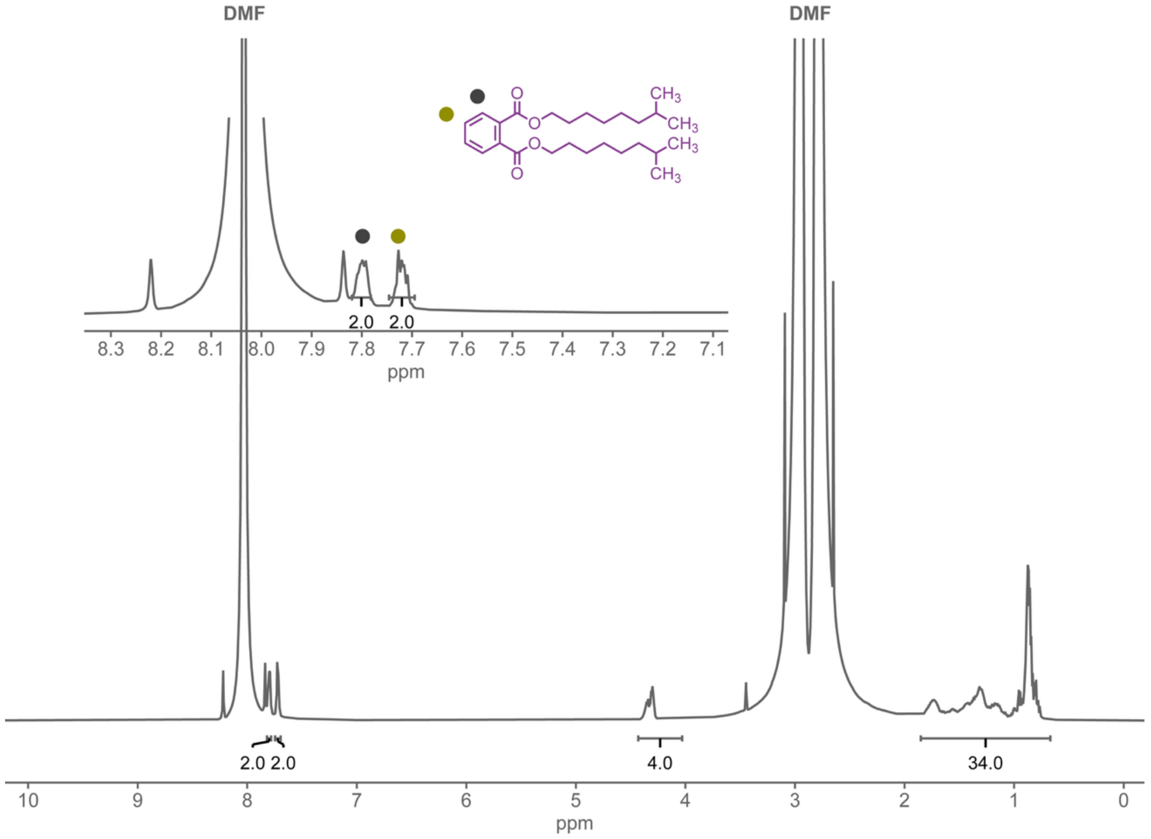

Figure S2. ^{1}H NMR spectrum of DINP. ^{1}H NMR (500 MHz, DMF) δ 7.80 (dd, 2H), 7.72 (m, 2H), 4.33 (m, 4H), 1.85 – 0.67 (m, 34H). The peak at 7.72 ppm was analyzed before and after electrolysis to determine the loss of DINP.

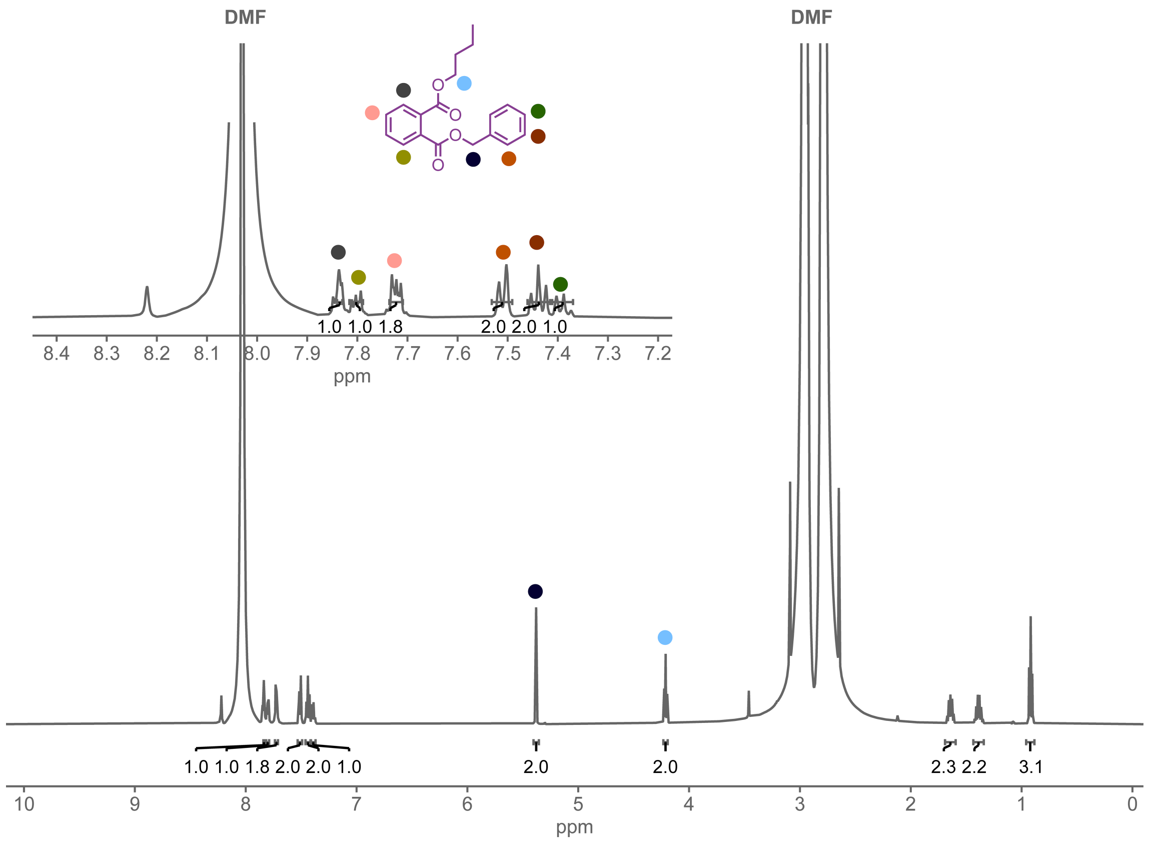

Figure S3. ^1H NMR spectrum of BBP. ^1H NMR (500 MHz, DMF) δ 7.84 (s, 1H), 7.82 – 7.79 (m, 1H), 7.74 – 7.71 (m, 2H), 7.51 (d, 2H), 7.44 (t, 2H), 7.39 (t, 1H), 5.38 (s, 2H), 4.21 (t, 2H), 1.64 (m, 2H), 1.39 (m, 2H), 0.92 (t, 3H). The peak at 5.38 ppm was analyzed before and after electrolysis to determine the loss of BBP.

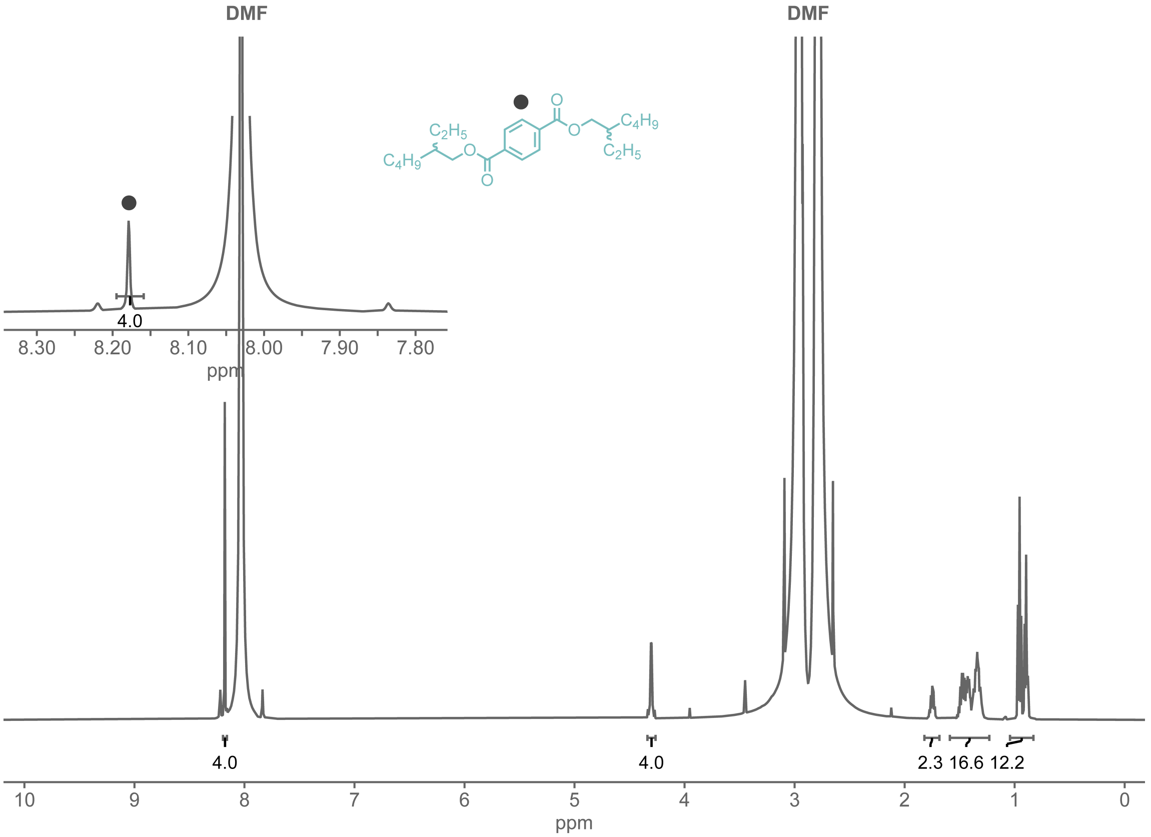

Figure S4. ^{1}H NMR spectrum of DOTP. ^{1}H NMR (500 MHz, DMF) δ 8.18 (s, 4H), 4.34 – 4.26 (m, 4H), 1.75 (m, 2H), 1.59 – 1.23 (m, 17H), 0.93 (m, 12H). The peak at 8.18 ppm was analyzed before and after electrolysis to determine the loss of DOTP.

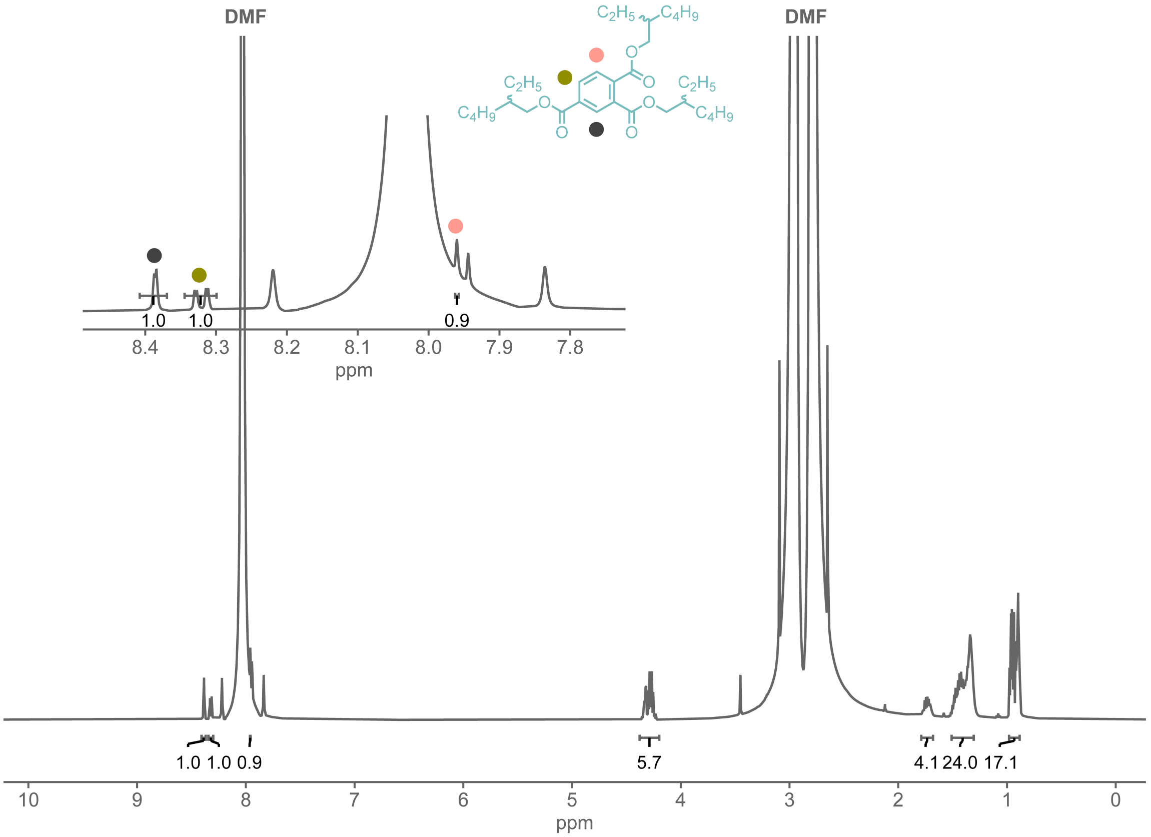

Figure S5. ^{1}H NMR spectrum of TOTM. ^{1}H NMR (500 MHz, DMF) δ 8.39 (d, 1H), 8.32 (dd, 1H), 7.96 (s, 1H), 4.29 (m, 6H), 1.73 (m, 4H), 1.51 – 1.31 (m, 24H), 0.98 – 0.88 (m, 17H). The peak at 8.39 ppm was analyzed before and after electrolysis to determine the loss of TOTM.

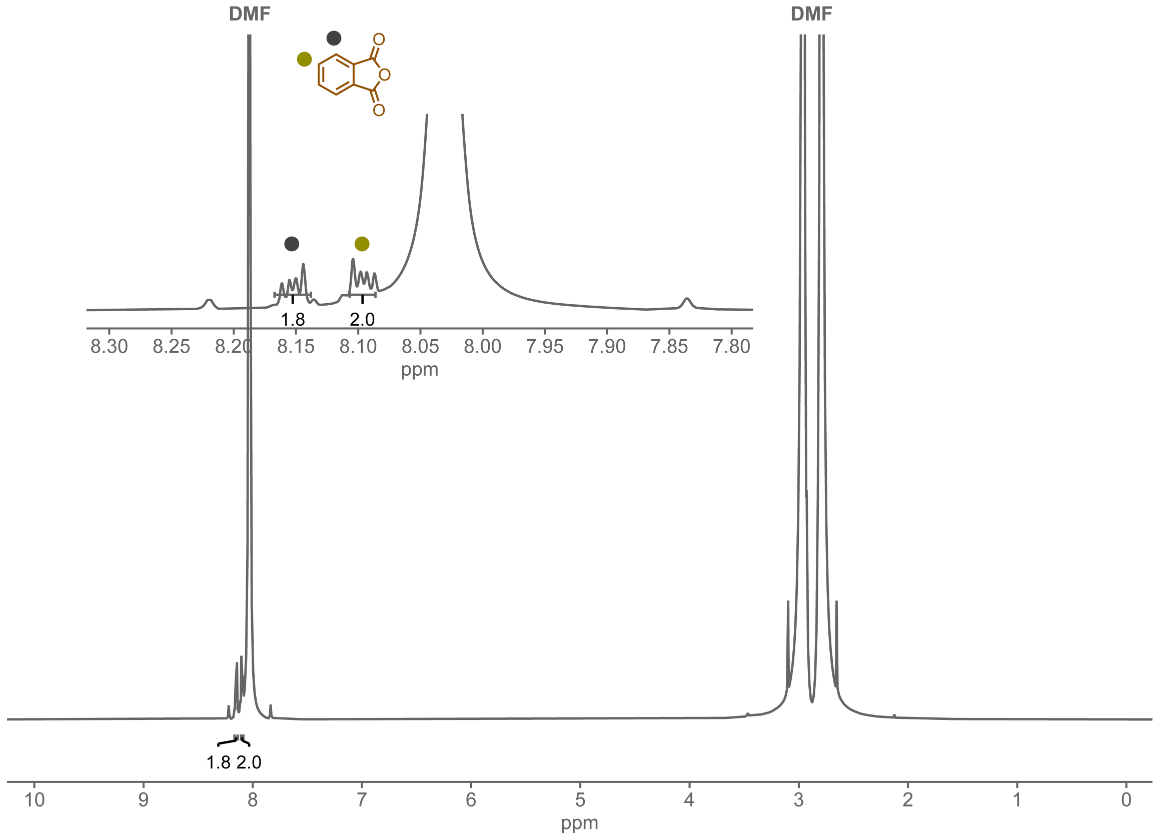

Figure S6. ^{1}H NMR spectrum of phthalic anhydride. ^{1}H NMR (500 MHz, DMF) δ 8.15 (m, 2H), 8.10 (m, 2H). The peak at 8.15 ppm was analyzed before and after electrolysis to determine the loss of phthalic anhydride.

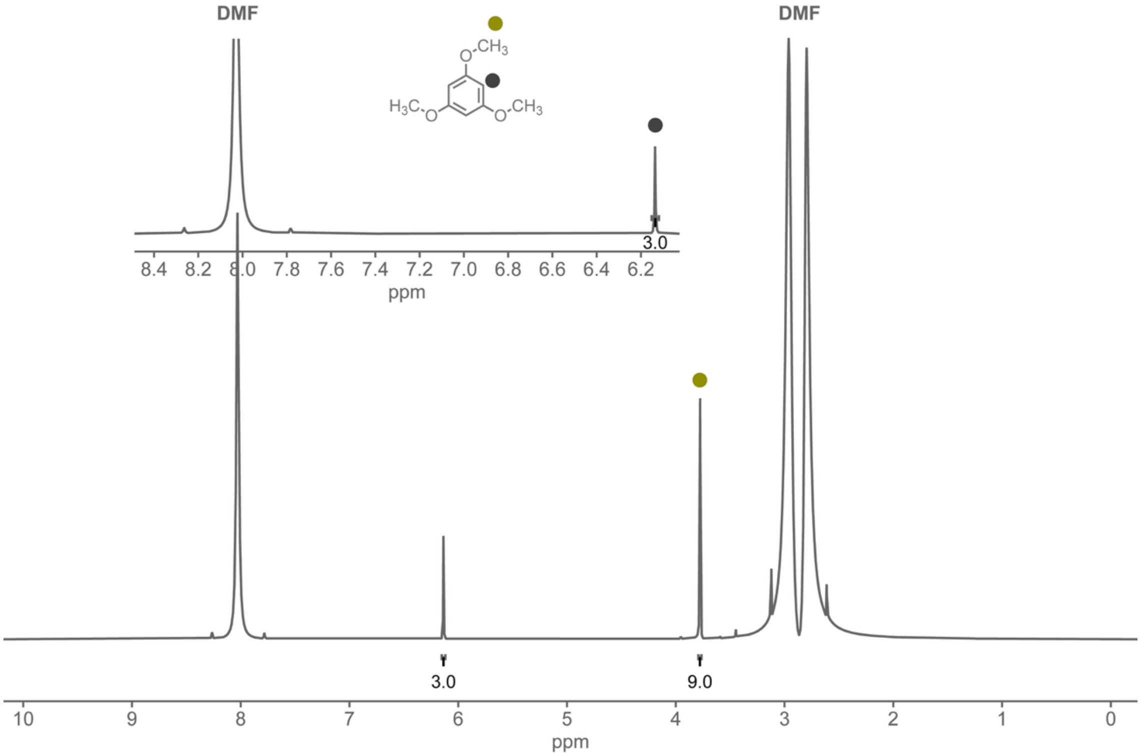

Figure S7. ^{1}H NMR spectrum of trimethoxybenzene (TMB). ^{1}H NMR (500 MHz, DMF) δ 6.14 (s, 3H), 3.78 (s, 9H). TMB was used as an internal standard to determine the loss of mediators. The peak at 6.14 ppm was analyzed before and after electrolysis to determine the loss of mediators.

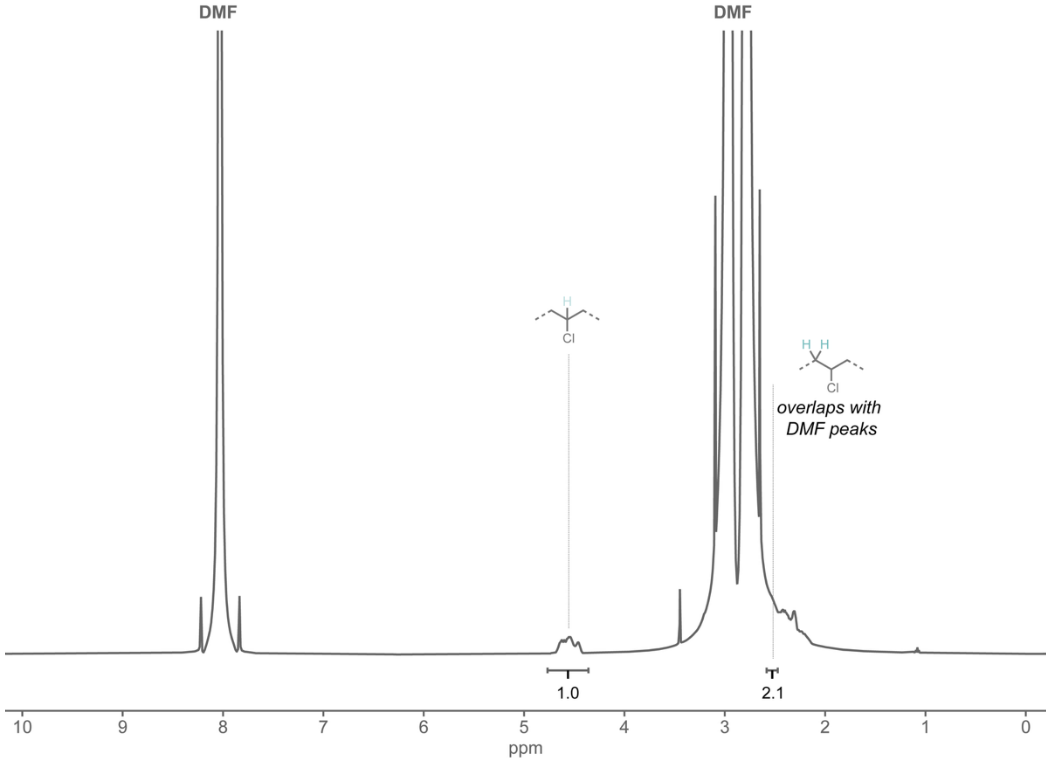

Figure S8. ^{1}H NMR spectrum of poly(vinyl chloride) (PVC_{37k}). ^{1}H NMR (500 MHz, DMF) δ 4.77 – 4.36 (m, 1H), 2.57 – 2.48 (m, 2H).

Section II.B. Representative mediator loss calculation via ^{1}H NMR spectroscopy

A 30 mM stock solution of trimethoxybenzene (TMB), which was used as a standard (std), was prepared by dissolving TMB (25 mg, 0.15 mmol) in anhydrous DMF (5.0 mL). A 0.20 mL aliquot from the working side of the divided cell was collected both before and after electrolysis using a pipette and was combined with 0.40 mL of the std stock solution. After thorough mixing, the solution was transferred to an NMR tube using a syringe. ^{1}H NMR spectra were then recorded for both the pre- and post-electrolysis samples. Because a non-deuterated solvent was used, manual gradient shimming was required. Shimming was performed by selecting the DMF peak at 8.03 ppm, using 4 scans with a 10 s delay and a pulse angle of 16.2 degrees. This process was completed in ~5 min. Following shimming, an ^{1}H NMR experiment was carried out at 500 MHz, using 16 scans with a 10 s delay and a pulse angle of 45 degrees. The spectra were referenced to the DMF solvent peak at 8.03 ppm (singlet) prior to integrating the relevant compound peaks. The aromatic peak of TMB at 6.14 ppm was integrated and normalized to the absolute integral value obtained from the global spectral deconvolution (GSD) using MestReNova software.

Calculation of TMB (std) concentration in the NMR tube:

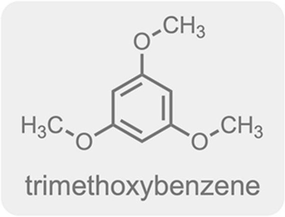

Final concentration of std in the NMR tube =

$$\frac{[concentration\ of\ std\ in\ the\ stock\ solution\ (mM)] \times [volume\ of\ std\ added\ to\ the\ NMR\ tube\ (mL)]}{total\ volume\ (volume\ of\ working\ side\ solution + volume\ of\ std)\ in\ NMR\ tube (mL)}$$

$$= \frac{30\ mM \times 0.4\ mL}{(0.2 + 0.4)\ mL} = 20\ \text{mM}$$

The concentration of mediator before and after the electrolysis via ^{1}H NMR spectroscopy was calculated using the following equation:

Concentration of mediator =

$$\frac{\left[\frac{absolute\ integral\ of\ assigned\ peak\ of\ mediator}{number\ of\ protons\ to\ that\ peak}\right] \times [concentration\ of\ std\ in\ the\ NMR\ tube\ (mM)]}{\frac{absolute\ integral\ of\ assigned\ peak\ of\ std}{number\ of\ protons\ to\ that\ peak}}$$

^{1}H NMR spectrum before electrolysis:

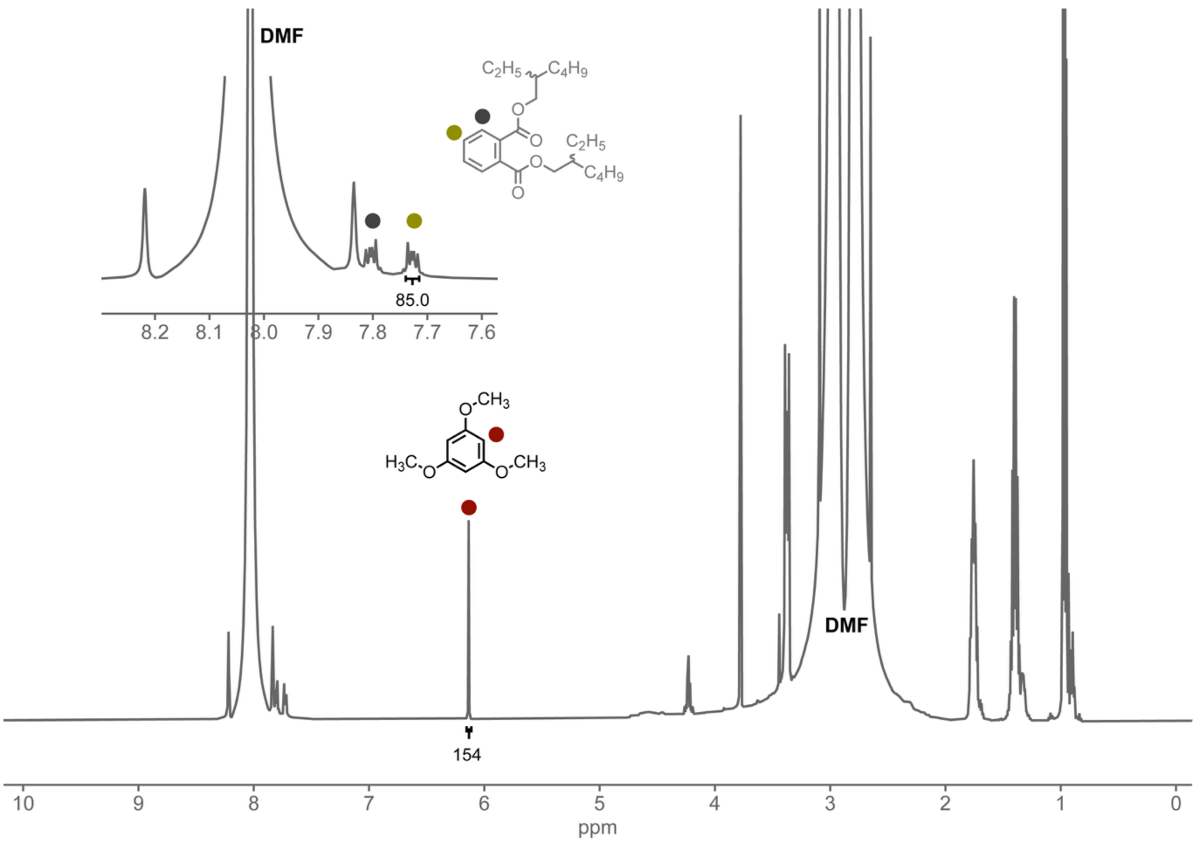

Figure S9. ^{1}H NMR spectrum of working side solution before electrolysis, containing DEHP, PVC_{37k}, NBu_4Cl, and DMF.

Table S1. The peak area for the aromatic protons of DEHP (m at 7.72 ppm) and TMB (s at 6.14 ppm) were extracted using the global spectral deconvolution function in MestReNova software.

ppm	peak area	total peak area
7.74	3.82	for DEHP aromatic proton (2 protons) = 85.0
7.74	24.1	
7.73	18.1	
7.72	15.8	
7.72	16.3	
7.71	6.85	
6.14	154	for TMB (at 6.14 ppm, 3 protons) = 154

Concentration of DEHP before electrolysis =

$$\frac{\frac{85.0}{2} \times 20.0\ mM}{\frac{154}{3}} = 16.6\text{ mM}$$

(intended concentration of DEHP = 16.7 mM)

^{1}H NMR spectrum after electrolysis:

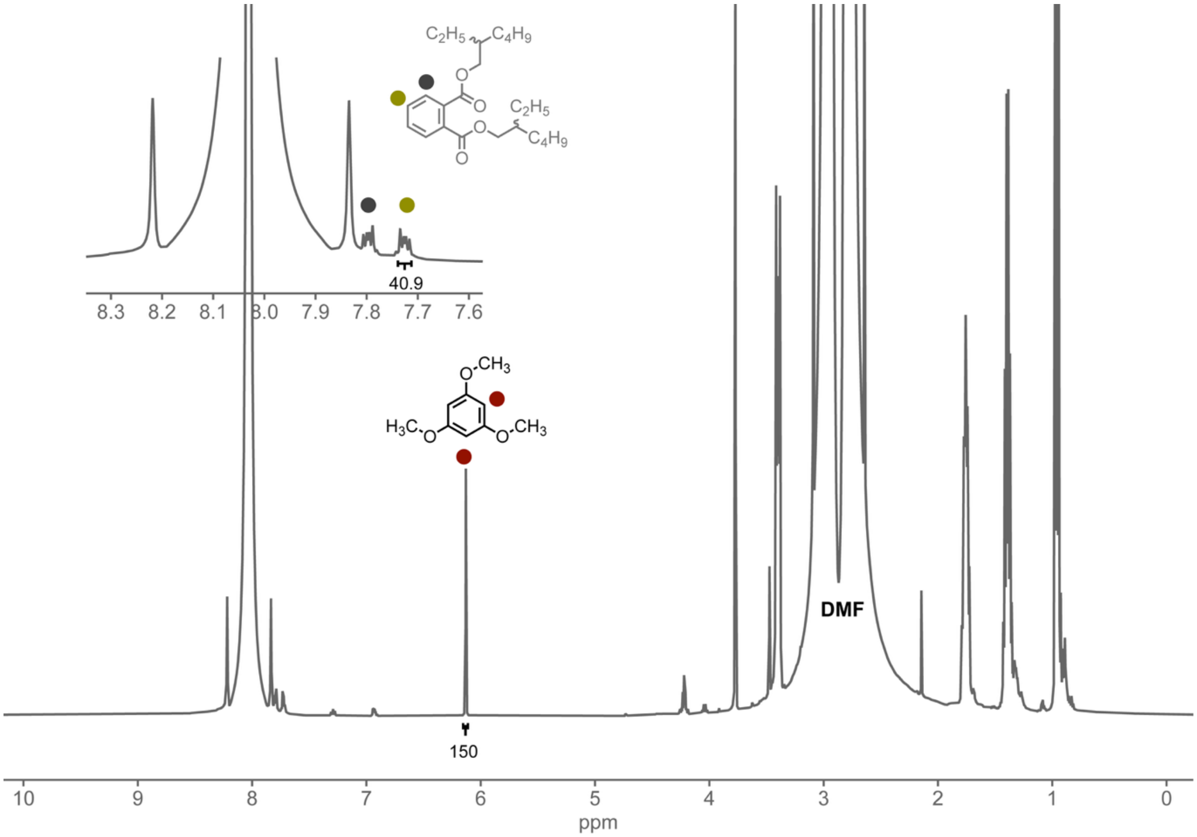

Figure S10. ^{1}H NMR spectrum of working side solution after electrolysis, containing DEHP, PVC_{37k}, NBu_4Cl, and DMF.

Table S2. The peak area for the aromatic protons of DEHP (m at 7.72 ppm) and TMB (s at 6.14 ppm) were extracted using the global spectral deconvolution function in MestReNova software.

ppm	peak area	total peak area
7.74	2.17	for DEHP aromatic proton (2 protons) = 40.9
7.74	11.4	
7.73	11.5	
7.72	6.32	
7.72	7.83	
7.71	1.72	
6.14	150	for TMB (at 6.14 ppm, 3 protons) = 150

Concentration of DEHP after electrolysis =

$$\frac{\frac{40.9}{2} \times 20.0\ mM}{\frac{150}{3}} = 8.18\ \text{mM}$$

Loss of DEHP after electrolysis =

$$\left(\frac{[concentration\ of\ DEHP\ before\ electrolysis] - [concentration\ of\ DEHP\ after\ electrolysis]}{concentration\ of\ DEHP\ before\ electrolysis}\right) \times\ 100\%$$

$$= \left(\frac{16.6\ mM\ - 8.18\ mM}{16.6\ mM}\right) \times\ 100\%$$

= 50.7%

Gas Chromatography–Mass Spectrometry (GC-MS)

GC-MS data were collected on a Shimadzu GC-2010 gas chromatograph equipped with a Restek™ Rtx™-5 capillary column with 15 m length, 0.25 mm inner diameter, 0.25 μm df (film thickness) with 5% diphenyl, 95% dimethylsiloxane as the stationary phase, with a Shimadzu GC-MS-QP2010S mass spectrometer. GC-MS data were analyzed using Shimadzu Corporation LabSolutions GC-MS solution Version 2.70 software. GC method: start and hold at 55 °C for 1 min, ramp 10 °C/min to 270 °C, hold at 270 °C for 10 min, total time: 32.5 min.

Sample preparation for GC-MS: A ~0.30 mL aliquot of the crude reaction mixture (both pre- and post-electrolysis), ~3.5 mL of diethyl ether, and 0.010 mL of tridecane standard were added in a scintillation vial using a pipette. The resulting solution was then filtered through a silica plug and a 0.20 μm PTFE filter into a GC vial.

Section II.C. Representative calculation of phenetole chlorination by GC-MS

GC-MS was analyzed for various materials used in this work (Figure S11).

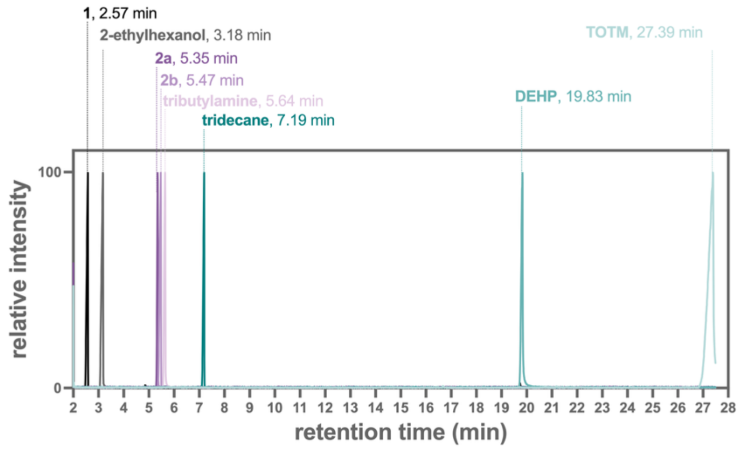

Figure S11. Overlaid GC-MS chromatograms of materials used in this work.

GC-MS response factor for 2-chloro-phenetole (**2a**) and 4-chloro-phenetole (**2b**) versus the starting material, phenetole (**1**), was determined using the procedure reported by Fagnani et al.[1]

response factor of **2a** versus **1** = 1.0 ± 0.07

response factor of **2b** versus **1** = 0.98 ± 0.03

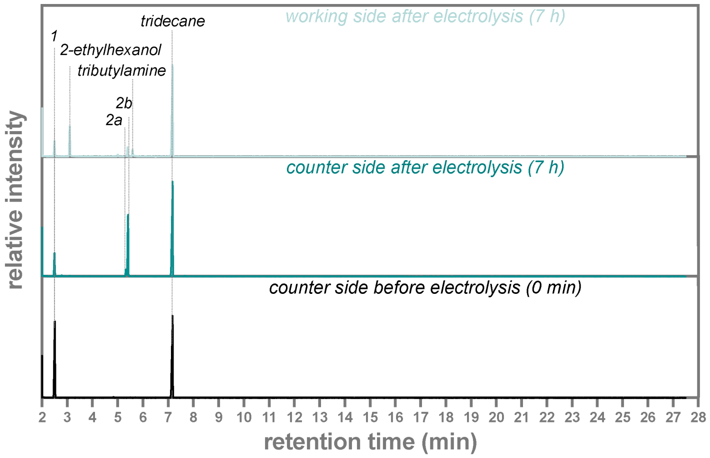

Figure S12. Representative GC-MS data collected before and after the electrolysis (Electrolysis Procedure E) using PVC_{37k} and TOTM in working side and phenetole in counter side for 7 h. Presence of 1, 2a, and 2b in the working side after the electrolysis confirms their crossover from the counter side. Presence of 2-ethylhexanol and tributylamine suggest the decomposition products of TOTM and NBu_4^+, respectively.

Table S3. Raw data extracted from representative GC-MS chromatograms (Figure S12).

compound	GC-MS peak area		
	counter side (0 min)	counter side (7 h)	working side (7h)
**1**	16303185	3688377	2478536
**2a**	0	998755	127685
**2b**	0	9998209	1412987
tributyl amine	0	0	1225881
tridecane	19553615	20100666	20471992
TOTM mediator	0	0	0
2-ethylhexanol	0	0	4983627

The fraction of each analyte (**1**, **2a**, and **2b**) relative to standard tridecane was calculated using the following equation

$$fraction\ relative\ to\ tridecane = \frac{area_{analyte}}{area_{tridecane}}$$

Table S4. Representative GC-MS data normalized to tridecane standard

compound	fraction relative to tridecane		
	counter side (0 min)	counter side (7 h)	working side (7 h)
**1**	0.834	0.183	0.121
**2a**	0.000	0.049	0.006
**2b**	0.000	0.497	0.069

<u>Assumption A</u><u>: consumption of **1** and yield of **2a** and **2b**, based on the initial concentration of **1**</u>

The percent consumption of **1** was calculated based on the remaining phenetole in the counter side after electrolysis with respect to the initial concentration of **1**. Note that this assumption does not take into consideration the amount of unreacted **1** that was crossed over.

$$\%\ of\ \mathbf{1}\ consumed\ = 1 - \left(\frac{fraction\ of\ \mathbf{1}\ at\ 7\ h\ in\ counter\ side}{fraction\ of\ \mathbf{1}\ at\ 0\ min\ in\ counter\ side}\right) \times 100\%$$

The percent conversion of **2a** and **2b** was determined by their amounts in the counter side after electrolysis, and any detected in the working side due to crossover. The relative formation of **2a** during electrolysis was determined using the following equation, in which 1.0 is used as the response factor for **2a** versus **1**:

$$\%\ yield\ of\ \mathbf{2a} = \left(\frac{fraction\ of\ \mathbf{2a}\ at\ 7\ h\ in\ counter\ side}{1.0\ *\ fraction\ of\ \mathbf{1}\ at\ 0\ min\ in\ counter\ side}\right) + \left(\frac{fraction\ of\ \mathbf{2a}\ at\ 7\ h\ in\ working\ side}{1.0\ *\ fraction\ of\ \mathbf{1}\ at\ 0\ min\ in\ counter\ side}\right)$$

The relative formation of **2b** during electrolysis was determined using the following equation, in which 0.98 is used as the response factor for **2b** versus **1**:

$$\%\ yield\ of\ \mathbf{2b} = \left(\frac{fraction\ of\ \mathbf{2b}\ at\ 7\ h\ in\ counter\ side}{0.98\ *\ fraction\ of\ \mathbf{1}\ at\ 0\ min\ in\ counter\ side}\right) + \left(\frac{fraction\ of\ \mathbf{2b}\ at\ 7\ h\ in\ working\ side}{0.98\ *\ fraction\ of\ \mathbf{1}\ at\ 0\ min\ in\ counter\ side}\right)$$

Total yield of **2** can be obtained by adding the individual yield of **2a** ***(7%)*** and **2b** ***(69%)***.

With Assumption A, the measured consumption of **1** is either greater than, or roughly comparable to, the yield of **2**. The discrepancy appears to stem from the separate addition of 0.01 mL tridecane to the counter and working side aliquots, both before and after electrolysis, for GC-MS analysis. This process led to varying concentration of tridecane in each vial.

To ensure consistency, we recommend preparing a stock solution of tridecane in diethyl ether and using it to transfer a fixed amount to each samples for GC-MS analysis, thereby maintaining the same tridecane concentration across all samples.

<u>Assumption B</u><u>: consumption of **1** and yield of **2a** and **2b**, based on the final concentration of **1 + 2a + 2b**</u>

$$[\mathbf{1} + \mathbf{2a} + \mathbf{2b}]_{after\ electrolysis} = (fraction\ of\ \mathbf{1} + \mathbf{2a} + \mathbf{2b})_{relative\ to\ tridecane\ in\ counter\ side\ at\ 7\ h} + fraction\ of\ \mathbf{1} + \mathbf{2a} + \mathbf{2b})_{relative\ to\ tridecane\ in\ working\ side\ at\ 7\ h}$$

Taking these values from Table S4,

$$[\mathbf{1}+\mathbf{2a}+\mathbf{2b}]_{at\ 7\ h}=0.927$$

(we are assuming here that there is no decomposition of phenetole during electrolysis, which may not be completely true)

The percent consumption of **1** was calculated based on the remaining phenetole in the counter side after electrolysis. This consumption also includes the presence of **1** in the working side due to crossover.

$$\%\ of\ \mathbf{1}\ consumed\ =1-\left(\frac{fraction\ of\ \mathbf{1}\ at\ 7\ h\ in\ counter\ side}{[\mathbf{1}+\mathbf{2a}+\mathbf{2b}]_{at\ 7\ h}}\right)$$

The percent conversion of **2a** and **2b** was determined by their amounts in the counter side after electrolysis, and any detected in the working side due to crossover. The relative formation of **2a** during electrolysis was determined using the following equation, in which 1.0 is used as the response factor for **2a** versus **1**:

$$\%\ yield\ of\ \mathbf{2a}=\left(\frac{fraction\ of\ \mathbf{2a}\ at\ 7\ h\ in\ counter\ side}{1.0\ *\ [\mathbf{1}+\mathbf{2a}+\mathbf{2b}]_{at\ 7\ h}}\right)+\left(\frac{fraction\ of\ \mathbf{2a}\ at\ 7\ h\ in\ working\ side}{1.0\ *\ [\mathbf{1}+\mathbf{2a}+\mathbf{2b}]_{at\ 7\ h}}\right)$$

The relative formation of **2b** during electrolysis was determined using the following equation, in which 0.98 is used as the response factor for **2b** versus **1**:

$$\%\ yield\ of\ \mathbf{2b}=\left(\frac{fraction\ of\ \mathbf{2b}\ at\ 7\ h\ in\ counter\ side}{0.98\ *\ [\mathbf{1}+\mathbf{2a}+\mathbf{2b}]_{after\ electrolysis}}\right)+\left(\frac{fraction\ of\ \mathbf{2b}\ at\ 7\ h\ in\ working\ side}{0.98\ *\ [\mathbf{1}+\mathbf{2a}+\mathbf{2b}]_{after\ electrolysis}}\right)$$

Total yield of **2** can be obtained by adding the individual yield of **2a** ***(9%)*** and **2b** ***(60%)***.

Table S5. Consumption of 1 and the generation of 2 from the representative GC-MS data (Figure S12).

compounds	assumption A: using $[\mathbf{1}]_{initial}$		assumption B: using $[\mathbf{1}+\mathbf{2a}+\mathbf{2b}]_{final}$	
	% consumption	% yield	% consumption	% yield
**1**	78	-	80	-
**2** (**2a** and **2b**)	-	76	-	69

Note: In the manuscript, we report yield by assumption B.

Section II.D. Representative calculation to determine Faradaic yield of phenetole chlorination

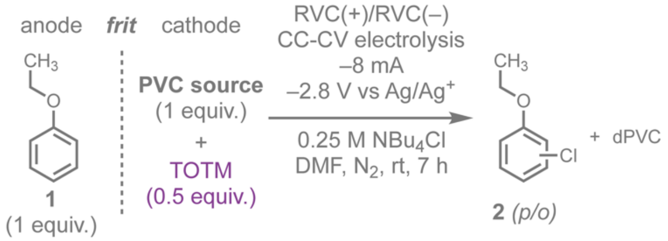

Scheme 1. Representative reaction scheme of GCPL electrolysis performed (Electrolysis Procedure E) using PVC_{37k} and TOTM in working side and phenetole in counter side for 7 h.

Capacity accessed during the electrolysis (Scheme 1) is 44.0 mAh.

$$charge\ passed = capacity\ (mAh) * \frac{1\ Ah}{1000\ mAh} * \frac{3600\ C}{1\ Ah} = 158\ C$$

Chlorination of phenetole (**1**) requires 2 electrons to produce per mole of chlorinated phenetole (Scheme 2).

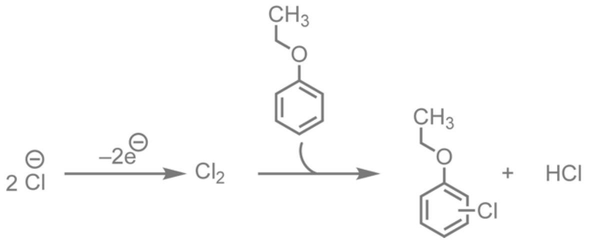

Scheme 2. Schematic of phenetole chlorination at anode.

Given 1 mmol of 1 used in the electrolysis, moles of 2 can be determined by its GC-MS yield (Table S5).

$$moles\ of\ \mathbf{2} = (GC - MS\ yield) * 1\ mmol$$

= 0.00076 mol (by assumption A)
= 0.00069 mol (by assumption B)

$$charge\ needed\ to\ generate\ \mathbf{2} = moles\ of\ \mathbf{2}\ (from\ GC - MS\ yield) * n * F$$

where, n = 2 and F = (96485 C/mol)

= 146 C (for assumption A)
= 133 C (for assumption B)

Now, the Faradaic yield can be calculated as following equation and summarized in Table S6.

$$Faradaic\ yield\ (\%) = \frac{charge\ used\ to\ form\ \mathbf{2}}{total\ charge\ passed} * 100$$

Table S6. Comparison of GC-MS yield of **2** (Section II.C.) with respect to the yield based on the capacity accessed.

yield of **2** (%)	GC-MS yield (assumption A)	Faradaic yield	GC-MS yield (assumption B)	Faradaic yield
	76	92	69	84

Note: In some cases, we observed >100% Faradaic yield, likely because undesired competitive reactions contributed to the accessed capacity.

Size Exclusion Chromatography (SEC)

For SEC analysis, all polymers were dried under vacuum overnight and dissolved (~1 mg polymer/mL) in THF spiked with toluene (<1 vol%) as a flow indicator and filtered through a 0.2 μm PTFE syringe filter. Polymer molar mass and dispersity was measured at 40 °C in THF on a Malvern Viscotek GPCMax VE2001 equipped with an Agilent PSS SDV 5 μm linear XL column (8 mm ID × 300 mm L) and an Agilent PSS SDV 5 μm 1000 Å column (8 mm ID × 300 mm L) and analyzed with Viscotek TDA 305. Apparent molar masses were calculated using PS standards (EasiCal PS-2, 10 standards, M_n: 555–348,500 g/mol) from Agilent. Data presented corresponds to the refractive index (RI) or ultra-violet (UV) response normalized to the highest peak. Data was obtained at a flow rate of 1 mL/min.

Table S7. SEC molar mass and dispersity for PVC_{37k} and PVC_{122k}.

PVC	source and product information	measured M_n (g/mol)	measured M_w (g/mol)	dispersity, *Đ*
PVC_{37k}	Sigma Aldrich<br>listed: M_w: ~43,000;<br>M_n: ~22,000	37,410 (trial 1)<br>37,840 (trial 2)	70,380 (trial 1)<br>69,990 (trial 2)	1.88 (trial 1)<br>1.85 (trial 2)
PVC_{122k}	Sigma Aldrich<br>listed: M_w: ~233,000;<br>*M*n: ~99,000	122,620 (trial 1)<br>121,960 (trial 2)	388,000 (trial 1)<br>398,260 (trial 2)	3.16 (trial 1)<br>3.26 (trial 2)

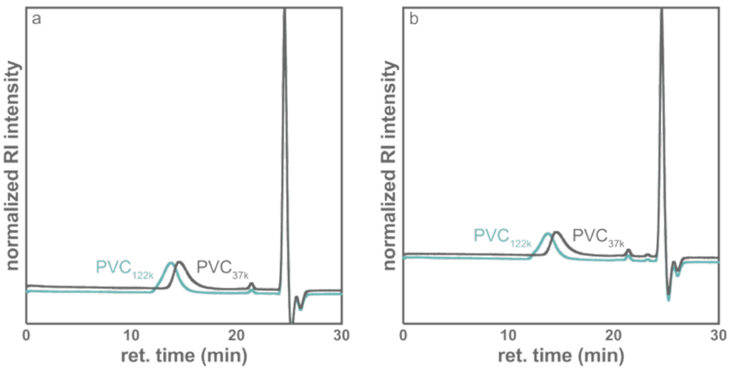

Figure S13. SEC RI chromatograms for trial 1 (a) and trial 2 (b) of PVC_{37k} and PVC_{122k}.

Fourier Transform Infrared (FTIR) Spectroscopy

FTIR spectra were obtained on solid samples of PVC and dechlorinated PVC (dPVC) using a Thermo-Nicolet IS-50 using the attenuated total reflectance (ATR) accessory. Samples (fine powder) were directly placed on the ATR crystal. Measurements were recorded between 4000–600 cm^{-1}, taking 64 scans at a resolution of 4 cm^{-1}.

Combustion Ion Chromatography (CIC)

CIC was performed by Atlantic Microlab, Inc. to determine the chlorine content in PVC and dPVC samples. Samples (~15 mg) were dried overnight (~15 h) under high vacuum at 25 °C before sending for analysis.

CIC protocol was provided by Atlantic Microlab, Inc., which is as follows:
Halogen analyses are performed by Schoniger Flask Combustion followed by analysis using Ion Chromatography. The sample is diluted to either 25 mL, 50 mL, or 75 mL, filtered and injected into the IC. The data is processed to yield the ppm of each halogen and then converted to percentages by the following calculation: ppm * Volume (L) / sample weight (kg) * 10000 (10000 ppm = 1%).

Carbon, hydrogen and nitrogen analyses are performed on automatic analyzers which utilize a technique based on a modification of the classical Pregl and Dumas methods. These analyzers are comprised of the following makes and models: Perkin-Elmer Model 2400 Series II Autoanalyzers and Carlo Erba Model 1108 Analyzers. Each instrument is calibrated daily with ultra-high purity standards prior to the analysis of any samples. In addition, standard runs with an appropriate ultra-high purity standard are made at regular intervals throughout the day to assure that instrument calibration is maintained. Instrument specifications list a precision of +/- 0.3%. Duplicate analyses, which are provided when sample size permits for compounds for which the

analyses do not fall within the range +/- 0.3% of calculated theoretical values, are frequently made on a different instrument by a different lab technician to further assure quality of results.

Section II.E. Calculation of % dechlorination in dechlorinated PVC

An estimate of % dechlorination in dechlorinated PVC (dPVC) was calculated using the following equation:

$$\%\,dechlorination = \left(1 - \frac{\%\,Cl\;dPVC}{\%\,Cl\;PVC}\right) * 100$$

Table S8. Combustion ion chromatography of PVC samples. PVC_{pipe} and PVC_{tubing} refer to the extracted PVC from pipe and tubing, respectively.

element	mass %					
	theoretical	experimental				
	PVC	PVC_{37k}	pipe	PVC_{pipe}	tubing	PVC_{tubing}
C	38.4	38.5	37.8	38.7	53.3	38.6
H	4.84	4.76	4.69	4.90	6.93	4.81
N	0.00	0.00	0.00	0.00	0.00	0.00
Cl	56.7	56.7	55.4	56.4	32.7	56.6
total	99.9	99.9	97.9	100	92.9	100

Thermogravimetric Analysis (TGA)

TGA thermograms were recorded using a TA Instruments Q50 TGA, which was calibrated with the Curie points of alumel and nickel standards. All experiments were conducted on platinum TGA sample pans under a nitrogen purge of 50 mL/min, with a heating rate of 5 °C/min. The TGA procedure involved the following steps: an initial ramp at 5 °C/min from 25–100 °C, an isothermal hold at 100 °C for 20 min, a subsequent ramp at 5 °C/min from 100–320 °C, another isothermal hold at 320 °C for 20 min, and a final ramp at 5 °C/min from 320–600 °C.[2]

Electrochemical Analysis

All electrochemical measurements were performed in a N_2-filled glovebox (MBraun Labmaster 130) with <0.5 ppm water and <10 ppm oxygen using a VSP Bio-Logic potentiostat equipped with EC-Lab software (V11.50) with voltage range ± 20 V, and current ranging from 10 μA up to 1 A.

Cyclic Voltammetry (CV)

All CV experiments were performed in anhydrous DMF. The solvent was chosen because of its wide electrochemical stability window, moderate ionic conductivity, compatibility with electrolyte salts, and ability to dissolve PVC. CV experiments were performed with 10 mM active species in a three-electrode electrochemical cell with a glassy carbon disc working electrode (0.07 cm^2, BASi), a platinum wire counter electrode (0.5 mm, BASi), and a fritted (frit BASi CoralPor, 0.5 mm silver wire BASi) Ag/Ag^+ pseudo-reference electrode in 10 mM $AgBF_4$ in 0.1 M NBu_4BF_4 in

acetonitrile. This reference electrode was used regardless of the solvent and the supporting salt. Due to incompatibility of $AgBF_4$ in DMF (Ag^+ reduces to Ag in DMF),[3] the reference electrode is prepared with acetonitrile, which could induce a junction potential. Although all the redox potentials are reported versus Ag/Ag^+ in this work, a CV experiment with ferrocene was performed, and its oxidation potential was found to be 0.09 V (Figure S14). To convert potentials from Ag/Ag^+ to Fc/Fc^+, subtract 0.09 V (i.e., $E_{1/2}$ versus Fc/Fc^+ = $E_{1/2}$ versus Ag/Ag^+ – 0.09 V).

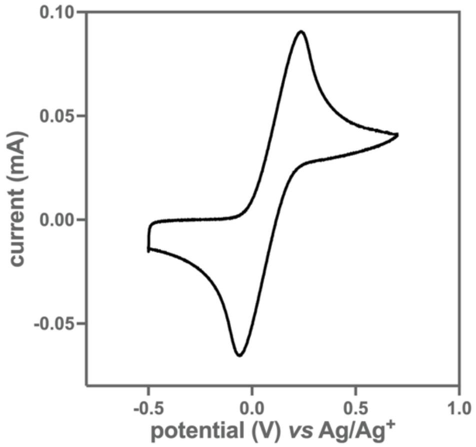

Figure S14. Cyclic voltammogram of 10 mM ferrocene at 25 mV/s collected in 0.1 M NBu_4BF_4 in DMF. The oxidation potential ($E_{1/2}$) of ferrocene is 0.09 V (versus Ag/Ag^+).

Chemical reversibility was determined via CV, using the ratio of peak height of the anodic and cathodic currents (I_{pa} / I_{pc} = 1). CVs were recorded at various scan rates (25 mV/s, 100 mV/s, and 500 mV/s). The reduction potential ($E_{1/2}$) was calculated as the average of the potentials at the peak cathodic current (E_{pc}) and peak anodic current (E_{pa}). For irreversible voltammograms, E_{pc} was used to determine the voltage limit instead of $E_{1/2}$.

Bulk Electrolysis (BE)

All small scale BE experiments were performed in a custom-made H-cell, with both sides separated by an ultrafine glass frit (ROBU VitraPor™, 10 mm * 2.8 mm, porosity 5) as shown in Figure S15. Scale up studies were performed in the H-cell shown in Figure S16. Reticulated vitreous carbon (RVC) electrodes, ~5 cm long (McMaster 100 PPI) were used as the working and counter electrodes. A fritted Ag/Ag^+ pseudo-reference electrode in 10 mM $AgBF_4$ in 0.30 M or 0.25 M (depending on the supporting electrolyte concentration in electrolysis cell) NBu_4BF_4 in acetonitrile was placed in the working chamber. Reduction and oxidation reactions proceeded in the working and counter side, respectively. The counter chamber was loaded with phenetole and the working side was loaded with plasticizer and/or PVC, dissolved in either 0.3 M NBu_4BF_4 or 0.25 M NBu_4Cl in DMF. Small-scale electrolyses were performed in a N_2-filled glovebox at room

temperature. Large-scale electrolysis was performed in a fume hood under N_2 at room temperature.

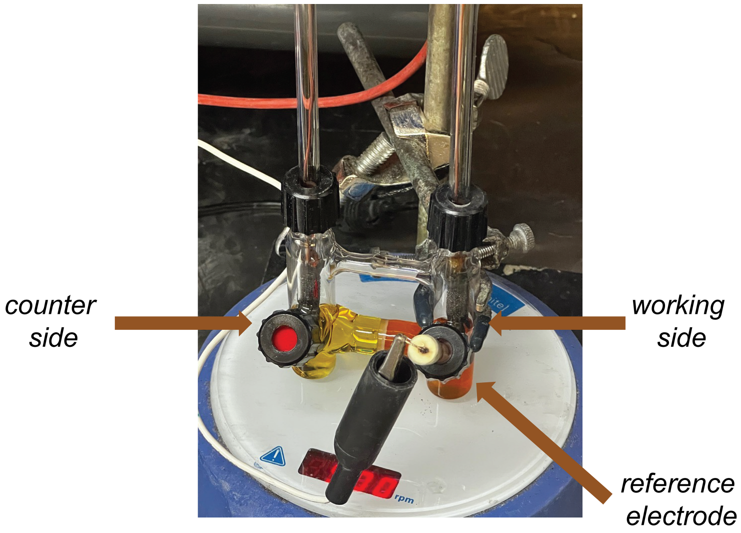

Figure S15. H-cell used for bulk electrolysis experiments. Both the working and counter chambers consisted of 5 mL total volume each.

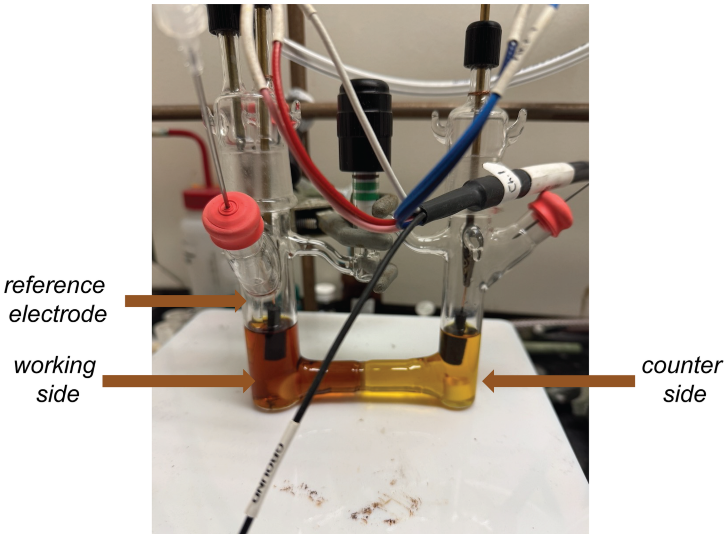

Figure S16. H-cell used for scale-up electrolysis experiments. Working side contains 25 mL volume and the counter side contain 20 mL volume.

Section II.F. Representative example to calculate theoretical capacity

The theoretical capacity of 5.0 mL of a 50 mM DEHP solution (0.25 mmol) was calculated as follows:

$$0.25 \text{ mmol DEHP} * \frac{1 \text{ mol}}{1000 \text{ mmol}} * \frac{96485 \text{ C}}{1 \text{ mol}} * \frac{1 \text{ A} * \text{s}}{1 \text{ C}} * \frac{1000 \text{ mA}}{1 \text{ A}} * \frac{1 \text{ h}}{3600 \text{ s}} = 6.70 \text{ mAh}$$

where 96485 C/mol is the Faraday constant. Similarly, when PVC (1.0 mmol) is added, the theoretical capacity due to PVC is calculated to be 26.8 mAh. Therefore, the combined theoretical capacity becomes 33.5 mAh.

It is important to note that these capacities are calculated assuming no side reactions occur. However, redox-active decomposition products and other electrochemical side reactions can cause the observed capacity to exceed the theoretical value, even when quantitative PVC dechlorination is achieved.

Section II.G. Demonstration of electrolyses parameters used in this study

Method 1: Electrolysis via chronopotentiometry (CP)

In this method, the reaction starts under a constant current, using the chronopotentiometry (CP) mode on the BioLogic potentiostat (Figure S17). The system is configured to hold the current steady until a predefined potential limit (mediator's $|E_{1/2}|$ + 300 mV). Once the reaction hits this potential limit, the electrolysis is automatically terminated.

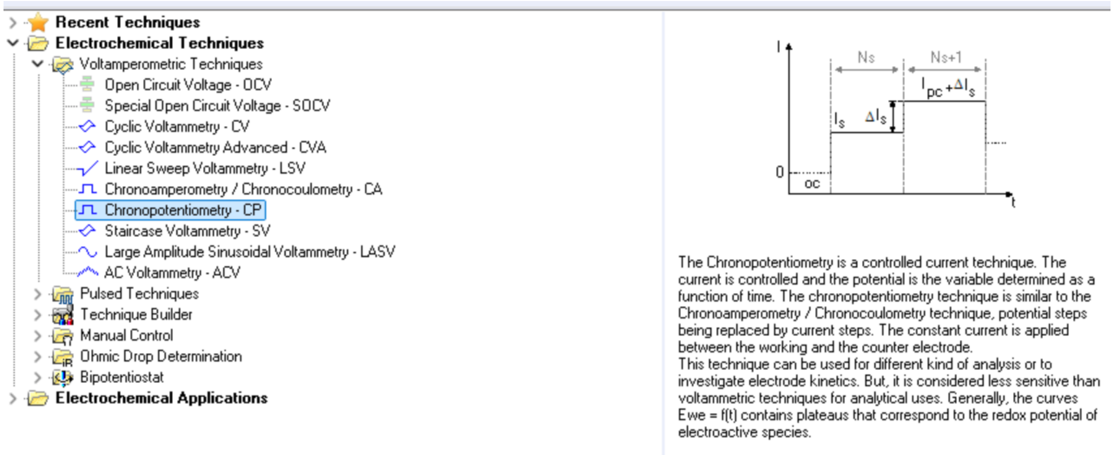

Figure S17. The CP method in the potentiostat used for electrolysis.

The electrolysis parameters are summarized in Figure S18. In this example, the applied current (I_s) is set to –8 mA, with a potential limit at –2.69 V. The chosen runtime (t_s) of 5 h is arbitrary and deliberately set in excess, ensuring that duration does not constrain the reaction prior to reaching

the potential limit. The working electrode potential (E_{we}) is continuously recorded at 1-second intervals (dt_s = 1 s). It is important to adjust both the potential (E range) and current (I range) parameters in accordance with the applied current (I_s) and the potential limit (E_M).

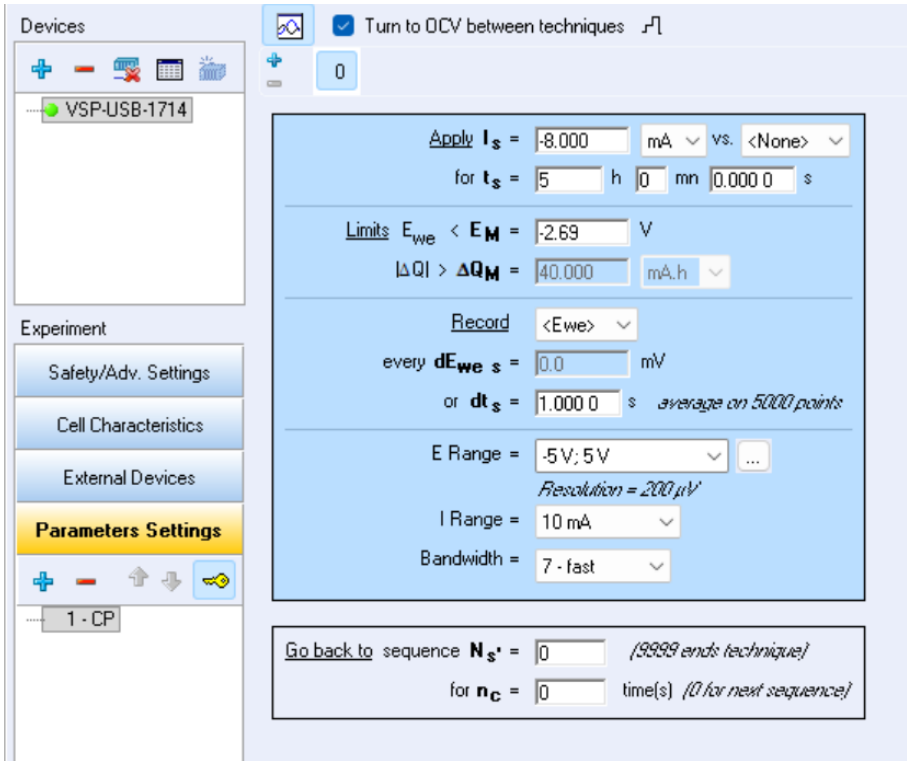

Figure S18. Example of CP parameters used for electrolysis.

Method 2: Electrolysis via galvanostatic cycling with potentiostatic limitation (GCPL)

In this approach, electrolysis was performed under constant current with a potential limitation (set at mediator's $|E_{1/2}|$ + 300 mV). The GCPL function on the BioLogic potentiostat (Figure S19) enables the system to maintain the potential limit. Please note that, during the potential hold phase, the current is no longer constant but declines gradually due to the increased solution resistance.

This technique is typically used for both charging and discharging modes, depending on the direction of the applied current. For our purposes, we focused solely on the charging phase, as the reduction of the mediator in the presence of PVC is sufficient to achieve dechlorination.

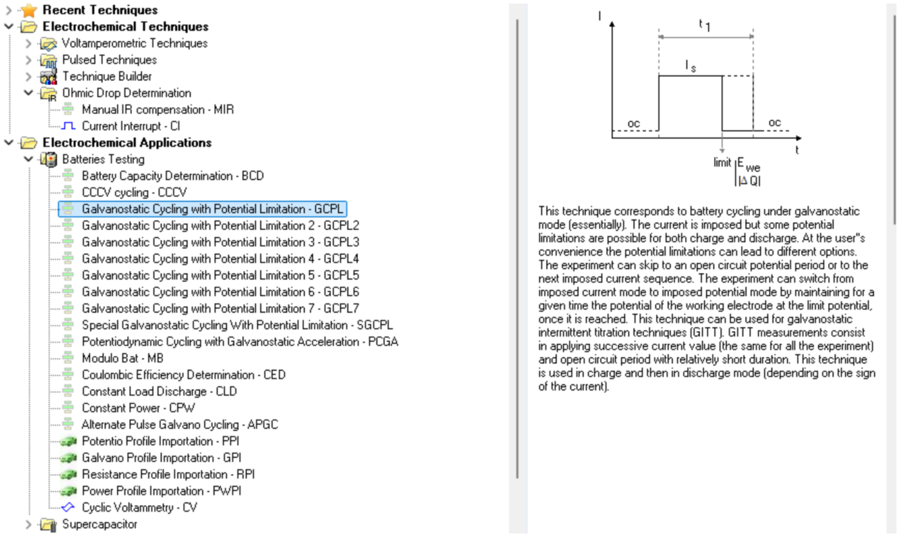

Figure S19. The GCPL method in the potentiostat used for electrolysis.

In box 1 (Figure S20), the applied current (I_s) is set to –8 mA, with a potential limit (E_M) at –2.69. The duration (t_1) is arbitrarily set to 6 h, providing amole time to ensure the reaction reaches the potential threshold without being time-limited. Potential is recorded at 1-second intervals (dt_1 = 1 s).

Upon reaching the potential limit (E_M), the procedure transitions to the next step, wherein the potential is held for a required duration. Here t_M represents this period and is also arbitrary chosen, as we manually terminate the reaction after a total 7 h (which includes the time taken to reach E_M and the hold time at E_M). We chose 7 h because due to solution resistance and polymer precipitation, current declines below –1.5 mA. The holding duration (t_M) can be adjusted according to experimental requirements. Additionally, the parameter I_m offers the option to automatically halt the reaction upon reaching a specific current threshold.

Both the potential (*E range*) and current (*I range*) limits should be tailored to the respective applied current (I_s) and potential limit (E_M). As we did not perform repeated charging cycles, the settings in Boxes 2, 3, and 4 were not utilized.

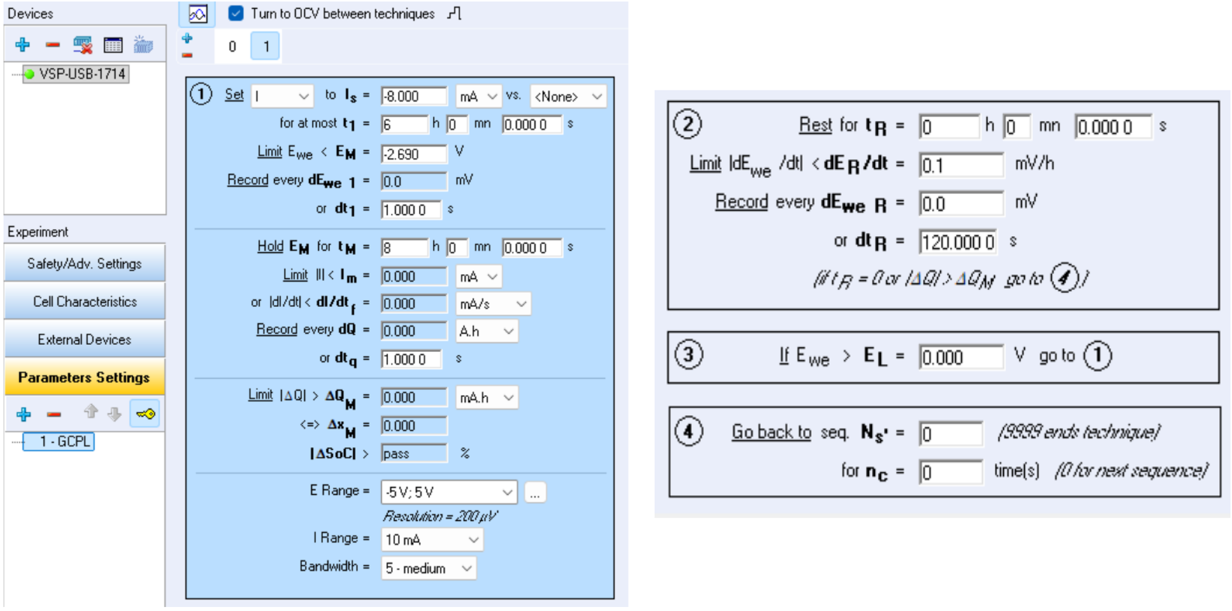

Figure S20. Example of GCPL parameters used for electrolysis.

III. Screening mediated behavior by cyclic voltammetry

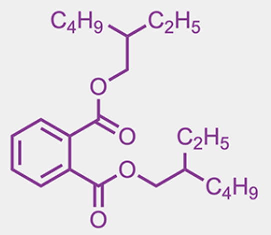

diethylhexyl phthalate (DEHP)

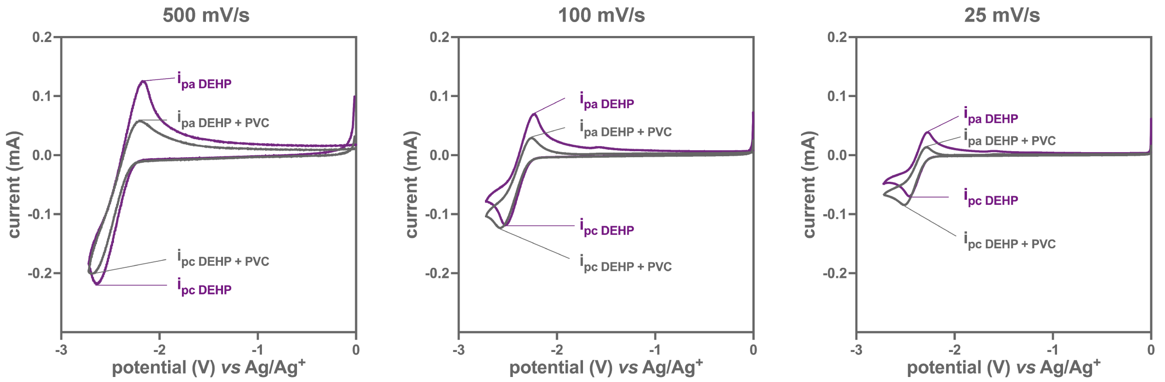

Figure S21. Cyclic voltammograms of 10 mM DEHP (purple) and 10 mM DEHP mixed with 30 mM PVC (gray) at 500 mV/s, 100 mV/s, and 25 mV/s collected in 0.1 M NBu_4BF_4 in DMF. The reduction potentials ($E_{1/2}$) of DEHP at 500 mV/s, 100 mV/s, and 25 mV/s are –2.40 V, –2.39 V, and –2.37 V (versus Ag/Ag^+), respectively. PVC_{37k} was used as PVC source.

Table S9. Current and voltage data extracted from CVs of 10 mM DEHP and 10 mM DEHP mixed with 30 mM PVC at 500 mV/s, 100 mV/s and 25 mV/s.

scan rate (mV/s)	10 mM DEHP			10 mM DEHP + 30 mM PVC_{37k}		
	i_{pc} (mA) reduction	i_{pa} (mA) oxidation	i_{pa} / i_{pc}	i_{pc} (mA) reduction	i_{pa} (mA) oxidation	i_{pa} / i_{pc}
500	0.21	0.25	1.2	0.18	0.16	0.89
100	0.11	0.12	1.1	0.11	0.12	1.1
25	0.070	0.070	1.0	0.080	0.070	0.88

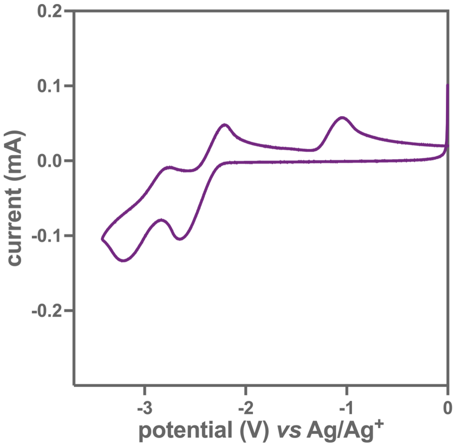

Figure S22. Cyclic voltammogram of 10 mM DEHP (purple) at 100 mV/s collected in 0.1 M NBu_4BF_4 in DMF. The first and second reduction potentials ($E_{1/2}$) of DEHP at 100 mV/s are –2.39 V and –2.91 V (versus Ag/Ag$^+$), respectively.

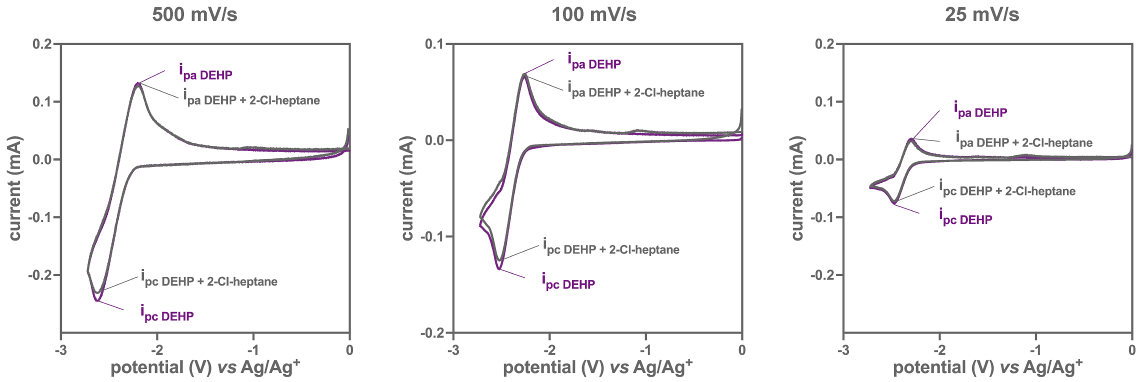

Figure S23. Cyclic voltammograms of 10 mM DEHP (purple) and 10 mM DEHP mixed with 30 mM 2-chloro-heptane (gray) at 500 mV/s, 100 mV/s, and 25 mV/s collected in 0.1 M NBu_4BF_4 in DMF. No appreciable mediation was observed.

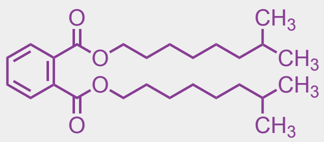

diisononyl phthalate
(DINP)

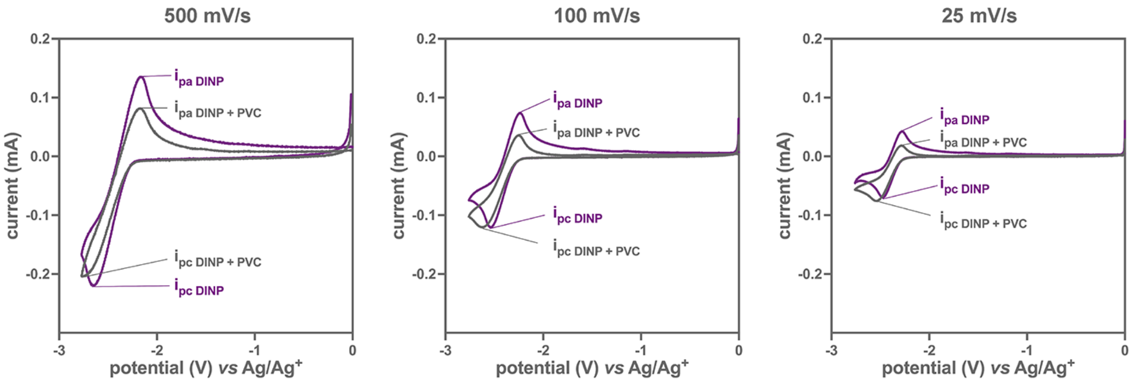

Figure S24. Cyclic voltammograms of 10 mM DINP (purple) and 10 mM DINP mixed with 30 mM PVC (gray) at 500 mV/s, 100 mV/s, and 25 mV/s collected in 0.1 M NBu_4BF_4 in DMF. The reduction potentials ($E_{1/2}$) of DINP at 500 mV/s, 100 mV/s, and 25 mV/s are –2.40 V, –2.38 V, and –2.38 V (versus Ag/Ag^+), respectively. PVC_{37k} was used as PVC source.

Table S10. Current and voltage data extracted from CVs of 10 mM DINP and 10 mM DINP mixed with 30 mM PVC at 500 mV/s, 100 mV/s and 25 mV/s.

scan rate (mV/s)	10 mM DINP			10 mM DINP + 30 mM PVC_{37k}		
	i_{pc} (mA) reduction	i_{pa} (mA) oxidation	i_{pa} / i_{pc}	i_{pc} (mA) reduction	i_{pa} (mA) oxidation	i_{pa} / i_{pc}
500	0.22	0.18	0.82	0.20	0.23	1.2
100	0.13	0.13	1.0	0.12	0.13	1.1
25	0.080	0.080	1.0	0.080	0.070	0.88

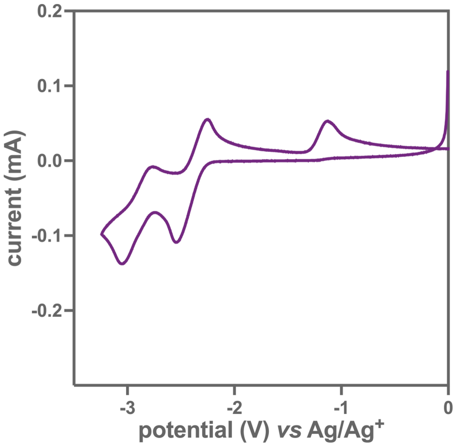

Figure S25. Cyclic voltammogram of 10 mM DINP (purple) at 100 mV/s collected in 0.1 M NBu_4BF_4 in DMF. The first and second reduction potentials ($E_{1/2}$) of DINP at 100 mV/s are –2.38 V and –2.91 V (versus Ag/Ag^+), respectively.

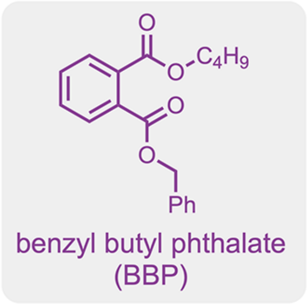

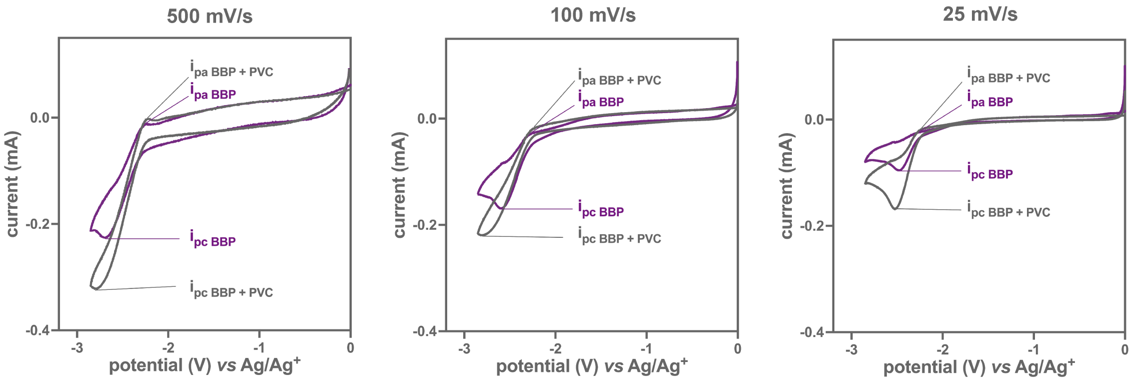

Figure S26. Cyclic voltammograms of 10 mM BBP (purple) and 10 mM BBP mixed with 30 mM PVC (gray) at 500 mV/s, 100 mV/s, and 25 mV/s collected in 0.1 M NBu_4BF_4 in DMF. BBP shows irreversible reduction at 500 mV/s, 100 mV/s, and 25 mV/s. PVC_{37k} was used as PVC source.

Table S11. Current and voltage data extracted from CVs of 10 mM BBP and 10 mM BBP mixed with 30 mM PVC at 500 mV/s, 100 mV/s and 25 mV/s; na: not applicable (due to irreversibility).

scan rate (mV/s)	10 mM BBP			10 mM BBP + 30 mM PVC_{37k}		
	i_{pc} (mA) reduction	i_{pa} (mA) oxidation	i_{pa} / i_{pc}	i_{pc} (mA) reduction	i_{pa} (mA) oxidation	i_{pa} / i_{pc}
500	0.17	na	na	0.28	na	na
100	0.12	na	na	0.20	na	na
25	0.060	na	na	0.14	na	na

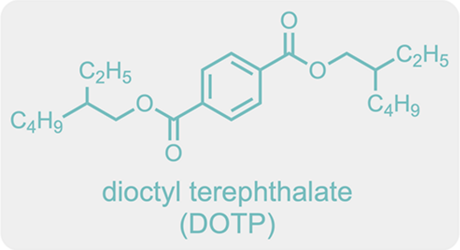

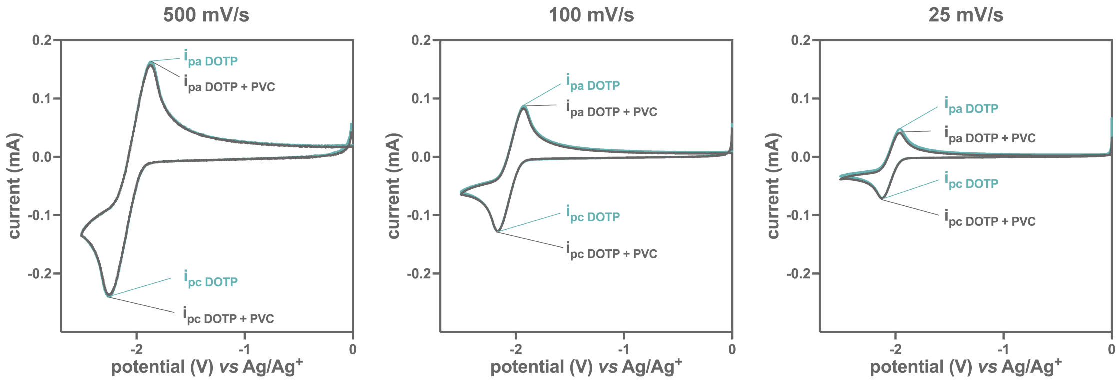

Figure S27. Cyclic voltammograms of 10 mM DOTP (teal) and 10 mM DOTP mixed with 30 mM PVC (gray) at 500 mV/s, 100 mV/s, and 25 mV/s collected in 0.1 M NBu_4BF_4 in DMF. The reduction potentials ($E_{1/2}$) of DOTP at 500 mV/s, 100 mV/s, and 25 mV/s are –2.06 V, –2.05 V, and –2.04 V (versus Ag/Ag^+), respectively. PVC_{37k} was used as PVC source.

Table S12. Current and voltage data extracted from CVs of 10 mM DOTP and 10 mM DOTP mixed with 30 mM PVC at 500 mV/s, 100 mV/s and 25 mV/s.

scan rate (mV/s)	10 mM DOTP			10 mM DOTP + 30 mM PVC_{37k}		
	i_{pc} (mA) reduction	i_{pa} (mA) oxidation	i_{pa} / i_{pc}	i_{pc} (mA) reduction	i_{pa} (mA) oxidation	i_{pa} / i_{pc}
500	0.24	0.25	1.0	0.23	0.24	1.0
100	0.12	0.13	1.1	0.12	0.13	1.1
25	0.070	0.070	1.0	0.070	0.070	1.0

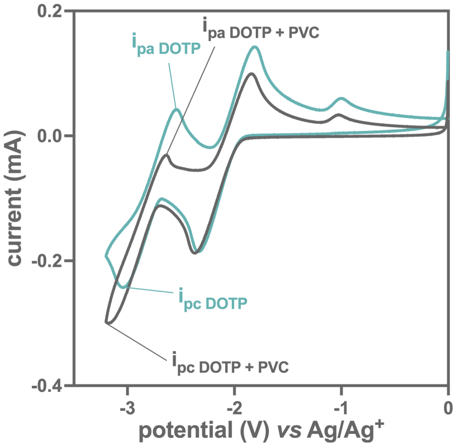

Figure S28. Cyclic voltammograms of 10 mM DOTP (teal) and 30 mM PVC (gray) at 100 mV/s collected in 0.1 M NBu_4BF_4 in DMF. The first and second reduction potentials ($E_{1/2}$) of DOTP at 100 mV/s are –2.05 V and –2.81 V (versus Ag/Ag^+), respectively. Accessing second reduction of DOTP shows mediated behavior for PVC reduction (grey). PVC_{37k} was used as PVC source.

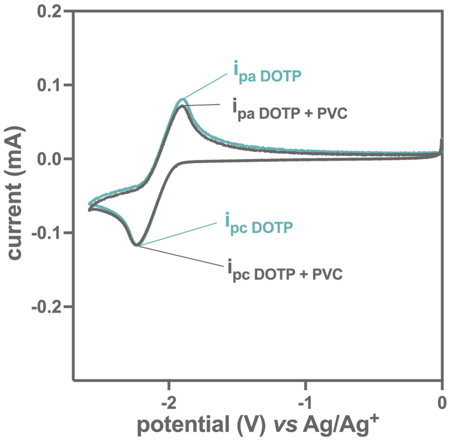

Figure S29. Cyclic voltammograms of 10 mM DOTP (teal) and 10 mM DOTP mixed with 60 mM PVC (gray) at 100 mV/s collected in 0.1 M NBu_4BF_4 in DMF. No mediation was observed after increasing the PVC equivalents (30 mM to 60 mM). PVC_{37k} was used as PVC source.

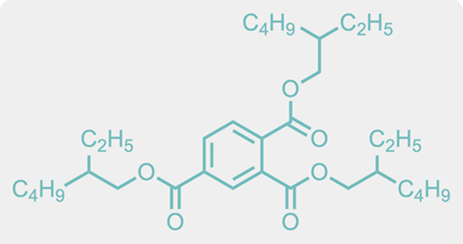

trioctyl trimellitate
(TOTM)

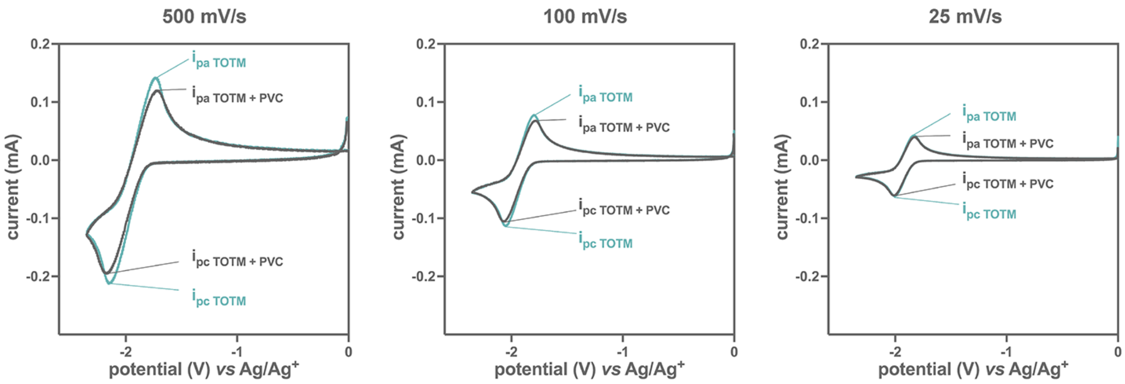

Figure S30. Cyclic voltammograms of 10 mM TOTM (teal) and 10 mM TOTM mixed with 30 mM PVC (gray) at 500 mV/s, 100 mV/s, and 25 mV/s collected in 0.1 M NBu_4BF_4 in DMF. The reduction potentials ($E_{1/2}$) of TOTM at 500 mV/s, 100 mV/s, and 25 mV/s are –1.94 V, –1.93 V, and –1.92 V (versus Ag/Ag^+), respectively. PVC_{37k} was used as PVC source.

Table S13. Current and voltage data extracted from CVs of 10 mM TOTM and 10 mM TOTM mixed with 30 mM PVC at 500 mV/s, 100 mV/s and 25 mV/s.

scan rate (mV/s)	10 mM TOTM			10 mM TOTM + 30 mM PVC_{37k}		
	i_{pc} (mA) reduction	i_{pa} (mA) oxidation	i_{pa} / i_{pc}	i_{pc} (mA) reduction	i_{pa} (mA) oxidation	i_{pa} / i_{pc}
500	0.21	0.24	1.1	0.19	0.23	1.2
100	0.11	0.12	1.1	0.11	0.11	1.0
25	0.060	0.060	1.0	0.060	0.060	1.0

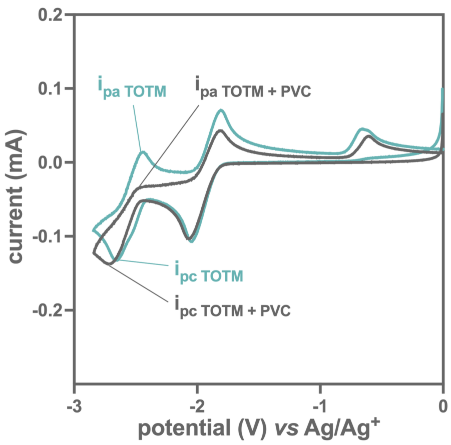

Figure S31. Cyclic voltammograms of 10 mM TOTM (teal) and 10 mM TOTM mixed with 30 mM PVC (gray) at 100 mV/s collected in 0.1 M NBu_4BF_4 in DMF. The first and second reduction potentials ($E_{1/2}$) of TOTM at 100 mV/s are –1.93 V and –2.56 V (versus Ag/Ag^+), respectively. Accessing second reduction of TOTM shows mediated behavior for PVC reduction. PVC_{37k} was used as PVC source.

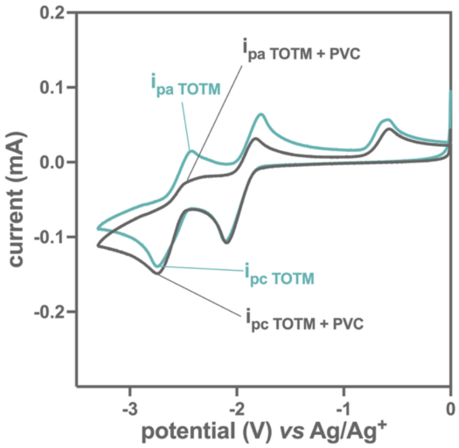

Figure S32. Cyclic voltammograms of 10 mM TOTM (teal) and 10 mM TOTM mixed with 60 mM PVC (gray) at 100 mV/s collected in 0.1 M NBu_4BF_4 in DMF. No mediation was observed at first reduction even after increasing the PVC equivalents (30 mM to 60 mM), although as observed in Figure S27, mediation occurred at 2nd reduction. PVC_{37k} was used as PVC source.

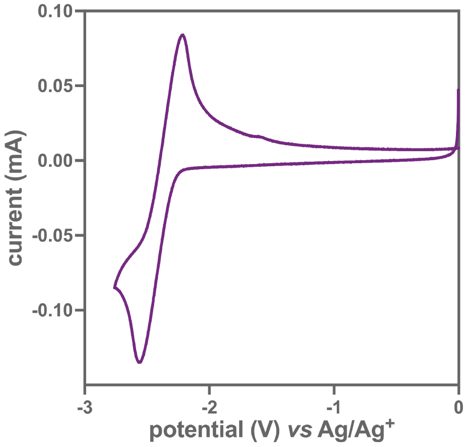

Figure S33. Cyclic voltammogram of 10 mM DEHP at 100 mV/s in 0.1 M NBu_4Cl in DMF. The reduction potential ($E_{1/2}$) of DEHP at 100 mV/s is –2.39 V versus Ag/Ag^+.

IV. CP and GCPL electrolysis with NBu_4BF_4 as supporting salt

Electrolysis Procedure A: (CP electrolysis, DEHP only, divided cell, 5.00 mL scale)

A stock solution of the supporting electrolyte was prepared by adding DMF (12.0 mL) to a vial containing NBu_4BF_4 (1180 mg, 3.60 mmol) using a pipette. Subsequently, 5.50 mL of this supporting electrolyte solution was transferred via pipette to a separate vial containing DEHP (107 mg, 0.275 mmol, 1.00 equiv.) to prepare the working side solution stock. Similarly, 5.50 mL of the supporting electrolyte solution was added by a pipette to a vial with phenetole (35.0 mg, 0.275 mmol, 1.00 equiv.) to prepare the counter side stock solution.

Next, 5.00 mL of the working side solution was dispensed using a pipette into the working compartment of an H-cell containing an RVC electrode and fritted Ag/Ag^+ as a working and reference electrode, respectively. An additional 0.200 mL aliquot from the remaining working side stock was reserved for ^1H NMR analysis to determine DEHP concentration before electrolysis (related to DEHP loss analysis, Section II.B). Simultaneously, 5.00 mL of the counter side solution was transferred to the counter compartment of the H-cell containing RVC counter electrode. Both working and counter side of the H-cell were equipped with a Teflon-coated magnetic stir bar and the reaction was stirred at 950 rpm at room temperature. The electrolysis was performed at constant current (–8 mA) with a voltage limit of –2.69 V (versus Ag/Ag^+). The reaction stopped once reaching the voltage limit. After the electrolysis, a 0.200 mL aliquot from the working side solution was taken for ^1H NMR spectroscopy to assess DEHP loss (Section II.B).

Electrolysis Procedure B: (CP electrolysis, DEHP and PVC, divided cell, 5.00 mL scale)

A stock solution of the supporting electrolyte was prepared by adding DMF (12.0 mL) to a vial containing NBu_4BF_4 (1180 mg, 3.60 mmol) using a pipette. Subsequently, 6.00 mL of this supporting electrolyte solution was transferred via pipette to a separate vial containing PVC_{37k} (75.0 mg, 1.20 mmol (repeat unit), 1.00 equiv. (repeat unit)) to prepare the PVC stock solution. Subsequently, 5.50 mL of this PVC stock solution was transferred via pipette to a separate vial containing DEHP (107 mg, 0.275 mmol, 0.250 equiv.) to prepare the working side solution stock. Similarly, 5.50 mL of the supporting electrolyte solution was added by a pipette to a vial with phenetole (134 mg, 1.10 mmol, 1.00 equiv.) to prepare the counter side stock solution.

Next, 5.00 mL of the working side solution was dispensed using a pipette into the working compartment of an H-cell containing RVC electrode and fritted Ag/Ag^+ as a working and reference electrode, respectively. An additional 0.200 mL aliquot from the remaining working side stock was reserved for ^1H NMR analysis to determine DEHP concentration before electrolysis (related to DEHP loss analysis, Section II.B). Simultaneously, 5.00 mL of the counter side solution was transferred to the counter compartment of the H-cell containing RVC counter electrode. Both working and counter side of the H-cell were equipped with a Teflon-coated magnetic stir bar and

the reaction was stirred at 950 rpm at room temperature. The electrolysis was performed at constant current (–8 mA) with a voltage limit of –2.69 V (versus Ag/Ag^+). The reaction stopped once reaching the voltage limit. After the electrolysis, a 0.200 mL aliquot from the working side solution was taken for ^1H NMR to assess DEHP loss (Section II.B). After the electrolysis, dPVC was extracted (Section IV.A) and sent for CIC to determine % dechlorination of PVC.

Electrolysis Procedure C: (GCPL electrolysis, DEHP and PVC, divided cell, 5.00 mL scale)

A stock solution of the supporting electrolyte was prepared by adding DMF (12.0 mL) to a vial containing NBu_4BF_4 (1180 mg, 3.60 mmol) using a pipette. Subsequently, 6.00 mL of this supporting electrolyte solution was transferred via pipette to a separate vial containing PVC_{37k} (75.0 mg, 1.20 mmol (repeat unit), 1.00 equiv. (repeat unit)) to prepare the PVC stock solution. Subsequently, 5.50 mL of this PVC stock solution was transferred via pipette to a separate vial containing DEHP (107 mg, 0.275 mmol, 0.250 equiv.) to prepare the working side solution stock. Similarly, 5.50 mL of the supporting electrolyte solution was added by a pipette to a vial with phenetole (134 mg, 1.10 mmol, 1.00 equiv.) to prepare the counter side stock solution.

Next, 5.00 mL of the working side solution was dispensed using a pipette into the working compartment of an H-cell containing RVC electrode and fritted Ag/Ag^+ as a working and reference electrode, respectively. Simultaneously, 5.00 mL of the counter side solution was transferred to the counter compartment of the H-cell containing RVC counter electrode. Aliquots were taken from the remaining working and counter side stocks for ^1H NMR analysis (related to DEHP loss analysis, Section II.B) and GC-MS analysis (related to phenetole chlorination, Section II.C), before electrolysis. Both working and counter side of the H-cell were equipped with a Teflon-coated magnetic stir bar and the reaction was stirred at 950 rpm at room temperature. The electrolysis was performed at constant current (–8 mA) with a voltage limit of –2.69 V (versus Ag/Ag^+). Once the reaction reached the voltage limit, electrolysis continued at this voltage until the total time of 7 h. Note that the total reaction time of 7 h consisted of ~2 h at CP until the voltage limit was reached, followed by the remaining time at the constant-voltage. At 7 h the current fell within the range of –1.2 to –1.5 mA, and the reaction was manually stopped. After the electrolysis, aliquots were taken for ^1H NMR (for DEHP loss analysis, Section II.B) and GC-MS analysis for determining the yield of Cl-phenetole (Section II.C). After the electrolysis, dPVC was extracted (Section IV.A) and sent for CIC to determine % dechlorination of PVC.

Section IV.A. Procedure for the isolation of dPVC after electrolysis

After the electrolysis, the solution from the working side was transferred into a centrifuge tube containing ~45 mL diethyl ether. At this stage, any solid polymer precipitate was also added to the centrifuge tube by using a spatula and diethyl ether. The resulting mixture was centrifuged at 4500 rpm for 10 min. The supernatant was decanted, leaving the precipitated dPVC at the bottom of the tube. Diethyl ether (~45 mL) is added and centrifuged to remove any remaining organic

compounds. The supernatant was decanted and the process was repeated two additional times. The obtained dPVC was then transferred into a vial and dried overnight (~15 h) under vacuum at room temperature before sending for characterization.

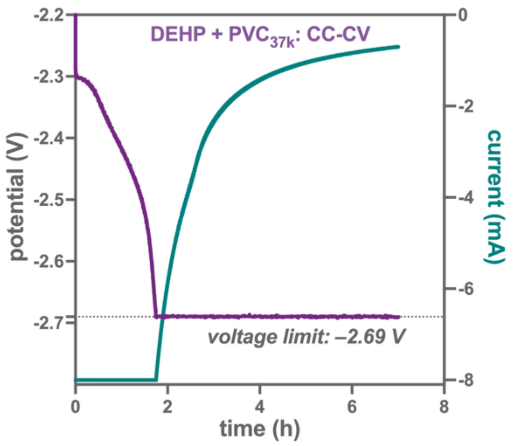

Figure S34. GCPL electrolysis of 0.25 equiv. DEHP and 1.0 equiv. PVC_{37k} in 0.30 M NBu_4BF_4 on the working side, with a current of –8 mA and a voltage limit of –2.69 V versus Ag/Ag^+. As the voltage limit is reached, the current began to decline due to increased resistance. The reaction was manually stopped at 7 h at which the current dropped below –1.5 mA.

Table S14. DEHP loss analysis calculated by ^{1}H NMR spectroscopy (see calculation in Section II.B). Electrolysis was performed as per Electrolysis Procedure A[a], B[b], and C[c].

experiment	peak area of DEHP at 7.72 ppm		peak area of TMB at 6.14 ppm		[DEHP] before BE	[DEHP] after BE	% DEHP loss
	before BE	after BE	before BE	after BE			
[a]CP with DEHP	88.1	67.0	153	138	17.3	14.5	16.2
[b]CP with DEHP + PVC_{37}	47.2	68.1	86.1	149	16.4	13.7	16.4
[c]GCPL with DEHP + PVC_{37k}	85.0	40.9	154	150	16.6	8.18	50.7

Table S15. Combustion ion chromatography of dPVC samples extracted (Section IV.A) from electrolysis as per Electrolysis Procedure B[a] and Electrolysis Procedure C[b]. Percent dechlorination was calculation as shown in Section II.D.

experiment	mass %					dechlorination (%)
	C	H	N	Cl	rest	
[a]CP with DEHP + PVC_{37k}	54.2	6.98	0.00	34.3	4.52	39.5
[b]GCPL with DEHP + PVC_{37k}	77.4	8.52	0.00	12.0	2.08	78.8

Table S16. Raw data extracted from GC-MS chromatograms recorded after a GCPL electrolysis with DEHP and PVC_{37} as per Electrolysis Procedure C. A capacity of 24.2 mAh capacity was accessed during electrolysis. Yield of Cl-phenetole (**2**) was calculated according to the representative calculations shown in Section II.C and Section II.D.

compounds	GC-MS peak area			yield of **2** (%)			
	c-0 min	c-7 h	w-7 h	GC-MS yield (**assumption A**)	Faradaic yield	GC-MS yield (**assumption B**)	Faradaic yield
**1**	25127965	19072005	1451782	2.00	2.00	2.20	2.20
**2a**	0	52051	0				
**2b**	0	393923	0				
tridecane	28506024	25359896	24927527				

V. GCPL electrolysis with NBu_4Cl as supporting salt

Electrolysis Procedure D: (GCPL electrolysis, DEHP and PVC, divided cell, 5.00 mL scale, NBu_4Cl as supporting electrolyte)

A stock solution of the supporting electrolyte was prepared by adding DMF (12.0 mL) to a vial containing NBu_4Cl (834 mg, 3.00 mmol) using a pipette. Subsequently, 6.00 mL of this supporting electrolyte solution was transferred via pipette to a separate vial containing PVC_{37k} (75.0 mg, 1.20 mmol (repeat unit), 1.00 equiv. (repeat unit)) to prepare the PVC stock solution. Subsequently, 5.50 mL of this PVC stock solution was transferred via pipette to a separate vial containing DEHP (107 mg, 0.275 mmol, 0.250 equiv.) to prepare the working side solution stock. Similarly, 5.50 mL of the supporting electrolyte solution was added by a pipette to a vial with phenetole (134 mg, 1.10 mmol, 1.00 equiv.) to prepare the counter side stock solution.

Next, 5.00 mL of the working side solution was dispensed using a pipette into the working compartment of an H-cell containing RVC electrode and fritted Ag/Ag^+ as a working and reference electrode, respectively. Simultaneously, 5.00 mL of the counter side solution was transferred to the counter compartment of the H-cell containing RVC counter electrode. Aliquots were taken from the remaining working and counter side stocks for ^1H NMR analysis (related to DEHP loss analysis, Section II.B) and GC-MS analysis (related to phenetole chlorination, Section II.C), before electrolysis. Both working and counter side of the H-cell were equipped with a Teflon-coated magnetic stir bar and the reaction was stirred at 950 rpm at room temperature. The electrolysis was performed at constant current (–8 mA) with a voltage limit of –2.69 V (versus Ag/Ag^+). Once the reaction reached the voltage limit, electrolysis continued at this voltage until the total time of 7 h. Note that the total reaction time of 7 h consisted of the time at CP until the voltage limit was reached, followed by the remaining time at the constant-voltage. At 7 h the current fell within the range of –1.2 to –1.5 mA, and the reaction was manually stopped. After the electrolysis, aliquots were taken for ^1H NMR (for DEHP loss analysis, Section II.B) and GC-MS analysis for determining the yield of Cl-phenetole (Section II.C). After the electrolysis, dPVC was extracted (Section IV.A) and sent for CIC to determine % dechlorination of PVC.
Note: The mass of DEHP added was adjusted accordingly based on its equivalents.

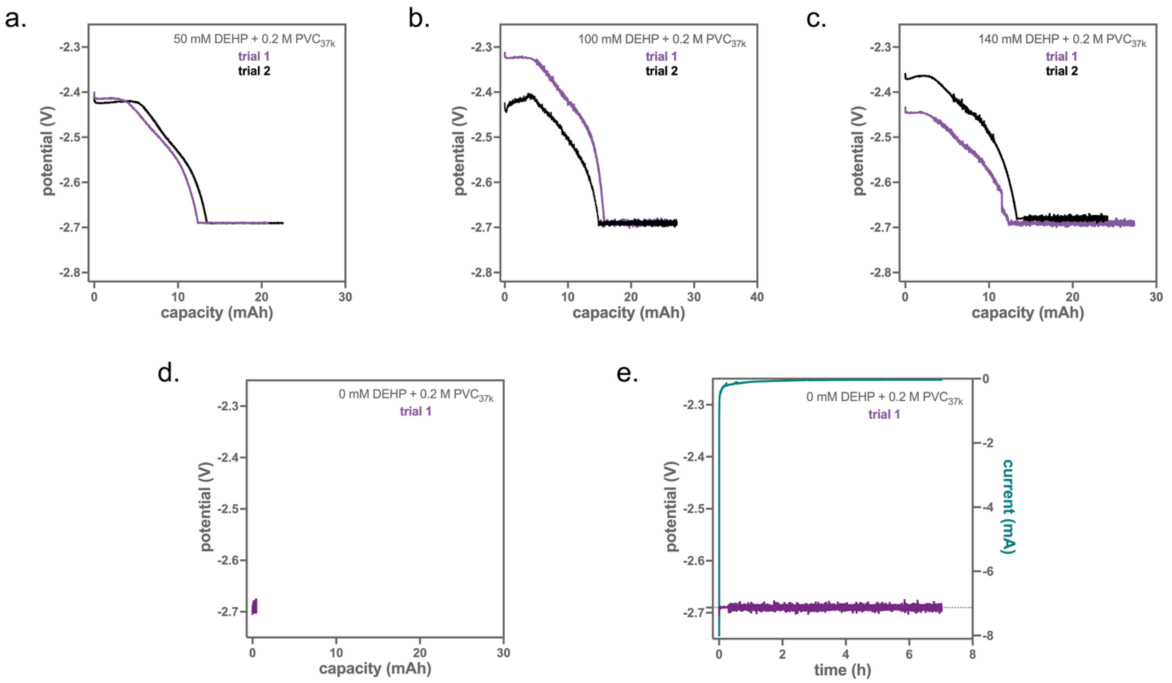

Figure S35. GCPL electrolysis of varying concentration of DEHP with 1 equiv. PVC_{37k} (0.2 M). The voltage versus capacity plots are shown for (a) 0.25 equiv. DEHP, (b) 0.5 equiv. DEHP, (c) 0.7 equiv. DEHP, and (d) 0 equiv. DEHP; Plot (e) shows voltage versus time and current profile for 0 equiv. DEHP, highlighting electrolysis immediately reached the voltage limit upon initiation. No significant capacity was accessed over 7 h, as the current remained ~0 throughout the reaction.

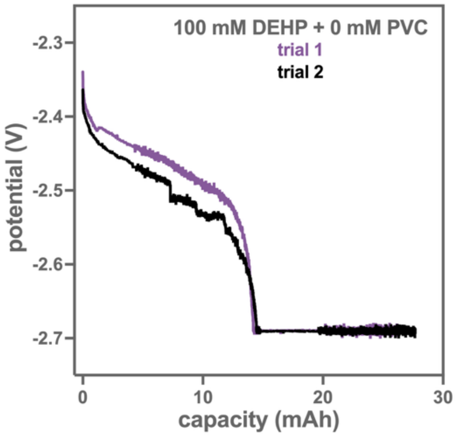

Figure S36. Plot of potential versus capacity obtained from GCPL electrolysis of 100 mM DEHP in 0.25 M NBu_4Cl in DMF on the working side, with a current of –8 mA and a voltage limit of –2.69 V versus Ag/Ag^+ for 7 h.

Table S17. DEHP loss analysis calculated by ^{1}H NMR spectroscopy (see calculation in Section II.B). Electrolysis was performed as per Electrolysis Procedure D.

experiment	peak area of DEHP at 7.72 ppm		peak area of TMB at 6.14 ppm		[DEHP] before BE	[DEHP] after BE	DEHP loss (%)
	before BE	after BE	before BE	after BE			
0.25 equiv. DEHP + 1 equiv. PVC_{37k} **trial 1**	104	59.8	149	157	20.9	11.4	45.4
0.25 equiv. DEHP + 1 equiv. PVC_{37k} **trial 2**	104	19.9	149	81.7	20.9	7.31	65.0
0.5 equiv. DEHP + 1 equiv. PVC_{37k} **trial 1**	96.1	52.4	82.3	85.2	33.0	18.4	44.2
0.5 equiv. DEHP + 1 equiv. PVC_{37k} **trial 2**	94.8	106	81.8	215	34.8	14.8	57.5
0.7 equiv. DEHP + 1 equiv. PVC_{37k} **trial 1**	128	155	75.2	123	51.1	37.9	25.8
0.7 equiv. DEHP + 1 equiv. PVC_{37k} **trial 2**	128	69.5	75.2	78.8	51.1	26.5	48.1
0.5 equiv. DEHP **trial 1**	93.9	32.0	84.5	91.0	33.3	10.5	68.5
0.5 equiv. DEHP **trial 2**	93.9	30.8	84.5	85.4	33.3	10.8	67.6

Table S18. Combustion ion chromatography of dPVC samples extracted (Section IV.A) from electrolysis as per Electrolysis Procedure D. % dechlorination was calculation as shown in Section II.D.

experiment	mass %					dechlorination (%)
	C	H	N	Cl	rest	
0.25 equiv. DEHP + 1 equiv. PVC_{37k} trial 1	73.2	8.80	0.120	10.3	7.58	81.8
0.25 equiv. DEHP + 1 equiv. PVC_{37k} trial 2	70.9	8.82	0.000	12.8	7.48	77.4
0.5 equiv. DEHP + 1 equiv. PVC_{37k} trial 1	75.5	8.27	0.380	5.83	10.0	89.7
0.5 equiv. DEHP + 1 equiv. PVC_{37k} trial 2	74.7	9.38	0.170	7.41	8.34	86.9
0.7 equiv. DEHP + 1 equiv. PVC_{37k} trial 1	75.2	9.75	0.280	5.78	8.99	89.8
0.7 equiv. DEHP + 1 equiv. PVC_{37k} trial 2	74.9	9.95	0.140	6.20	8.81	89.1
1 equiv. PVC_{37k} trial 1	38.9	4.85	0.000	56.2	0.05	0.900

Table S19. Raw data extracted from GC-MS chromatograms recorded after a GCPL electrolysis with DEHP and PVC_{37k} as per Electrolysis Procedure D. The capacity accessed during electrolysis is included with each experiments. Yield of **2** was calculated according to the representative calculations shown in Section II.C and Section II.D.

experiment and accessed capacity	compounds	GC-MS peak area			yield of **2** (%)			
		c-0 min	c-7 h	w-7 h	GC-MS yield (**assumption A**)	Faradaic yield	GC-MS yield (**assumption B**)	Faradaic yield
trial 1 0.25 equiv. DEHP + 1 equiv. PVC_{37k} (22.5 mAh)	**1**	27843210	16171757	2523901	47.0	112	40.0	95.0
	**2a**	0	1191094	0				
	**2b**	0	10728159	290672				
	tridecane	24804852	23402337	18792019				
trial 2 0.25 equiv. DEHP + 1 equiv. PVC_{37k} (20.7 mAh)	**1**	26023738	15326776	22322822	45	116	41.0	106
	**2a**	0	1071884	0				
	**2b**	0	10188303	503826				
	tridecane	21780276	22322822	21816076				
trial 1 0.5 equiv. DEHP + 1 equiv. PVC_{37} (26.0 mAh)	**1**	26023738	13619122	4279187	57.0	117	47.0	97.0
	**2a**	0	1443082	90436				
	**2b**	0	13356992	905226				
	tridecane	21780276	23989868	21409669				
trial 2 0.5 equiv. DEHP + 1 equiv. PVC_{37} (27.3 mAh)	**1**	14083477	6775575	1200620	63	124	52.0	102
	**2a**	0	849963	0				
	**2b**	0	7531640	193196				
	tridecane	17969746	17485332	16816102				
trial 1 0.7 equiv. DEHP + 1 equiv. PVC_{37k} (27.4 mAh)	**1**	18751224	8594169	2022200	54.0	105	45.0	88.0
	**2a**	0	811931	16761				
	**2b**	0	7404888	266080				
	tridecane	26560929	22899941	24103615				
trial 2 0.7 equiv. DEHP + 1 equiv. PVC_{37k} (24.2 mAh)	**1**	18751224	9235868	1417399	44.0	97.4	40.0	88.6
	**2a**	0	685012	0				
	**2b**	0	5938011	227963				
	tridecane	26560929	21957459	25122658				
trial 1 0.5 equiv. DEHP (26.2 mAh)	**1**	23331300	12722960	4279187	59.0	121	45.0	92.0
	**2a**	0	1174241	90436				
	**2b**	0	11377465	905226				
	tridecane	22020156	21752572	21409669				
trial 2 0.5 equiv. DEHP (27.7 mAh)	**1**	23331300	13632089	3857938	55.0	106	42.0	81.3
	**2a**	0	1143812	66572				
	**2b**	0	10610319	727107				
	tridecane	22020156	21890696	22287372				

Section V.A. Potentiometric titration to determine dechlorination of PVC

Potentiometric titration was conducted using an aqueous 10 mM $AgNO_3$ to measure chloride ion concentration post-electrolysis, following the method used by Neyhouse et al. (Figure S36).[4] The samples (0.1 mL) from both the counter and working sides at the outset (0 min) and after electrolysis (7 h) were transferred their respective scintillation vial using a pipette. Deionized water (10 mL) was measured using a volumetric flask and added to each of those vials containing electrolysis samples. The resulting solution was stirred at 900 rpm prior to the titration. The open-circuit potential was recorded using a silver disc working electrode (CH Instruments) and a copper reference electrode, which consisted of a copper wire immersed in 0.1 M $CuSO_4/H_2O$ and separated by a glass frit. An initial baseline open-circuit potential was measured for ~30 s, followed by the addition of 10 mM $AgNO_3$ solution dispensed at a flow rate of 2.5 mL/min (the flow rate was calibrated and adjusted to 2.5 mL/min before starting the experiment), using a Masterflex peristaltic pump with L/S 13 Norprene tubing. The starting point (time = 0) was defined by the first deviation in potential from the established baseline. Titration data is plotted from this starting time (Figure S37a), and the equivalence point (t_{eq}, min) was identified as the point with the highest change in potential versus time (dE/dt) (Figure S37b). The slope at each time point is determined from the difference in time points recorded before and after. The dechlorination yield was determined as follows:

$Ag^+ + Cl^- = AgCl$
Hence, Mole of Ag^+ = mole of chloride ions =

$$teq\ (s) * \frac{1\ min}{60\ s} * flow\ rate\ (2.5\ mL/min) * \frac{1\ L}{1000\ mL} * \frac{0.01\ mol\ Ag^+}{1\ L} = teq\ (s) * (4.167 *\ 10^{-7})\ mol/s$$

A 0.1 mL sample was used to perform titration,

$[Cl^-]$ (M) = moles of chloride ions / volume (in L) = (moles of Cl^- / 10^{-4} L) = (t_{eq} * 0.004167) M

As the counter and working side of the divided cell consist of 5 mL volume each,

mmol of Cl^- = t_{eq} * 0.004167 mol/L * 5 mL = (t_{eq} * 0.020835) mmol

Table S20. Calculation of chloride ion concentration before and after electrolysis based on t_{eq} extracted from titration plots (Figure S37).

	before electrolysis (0 min)			after electrolysis (7 h)		
samples	t_{eq} (s)	Cl^- (mmol)	total Cl^- (mmol) at 0 h	t_{eq} (s)	Cl^- (mmol)	total Cl^- (mmol) at 7 h
counter side	58.6	1.22	2.46	68.8	1.43	2.82
working side	59.6	1.24		66.4	1.39	

Considering, ~50% phenetole (1 mmol) chlorination at the anode, utilization of Cl^{-} will be 0.6 mmol.

Total Cl^{-} post-electrolysis = 2.82 + 0.5 = 3.32 mmol

Hence, Cl^{-} due to PVC dechlorination = (3.32 – 2.46) = 0.86 mmol

Concentration of PVC used in the electrolysis = (0.2 M * 5 mL) = 1 mmol

% dechlorination of PVC = (0.86/1) * 100 = 86%

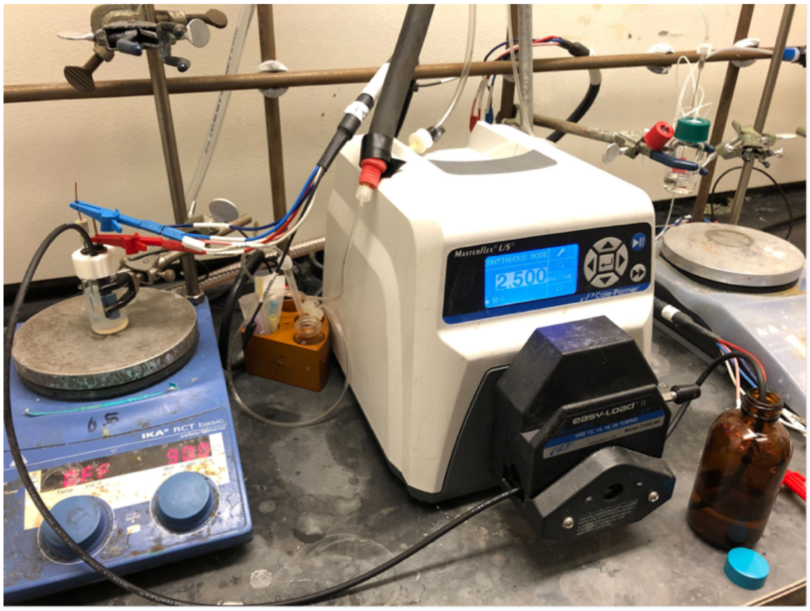

Figure S36. Photo of the potentiometric titration set up, showing the sample vial (containing unknown chloride ion) on the stir plate (left), peristaltic pump (middle), and 10 mM $AgNO_3$ titrant solution in an amber bottle (right).

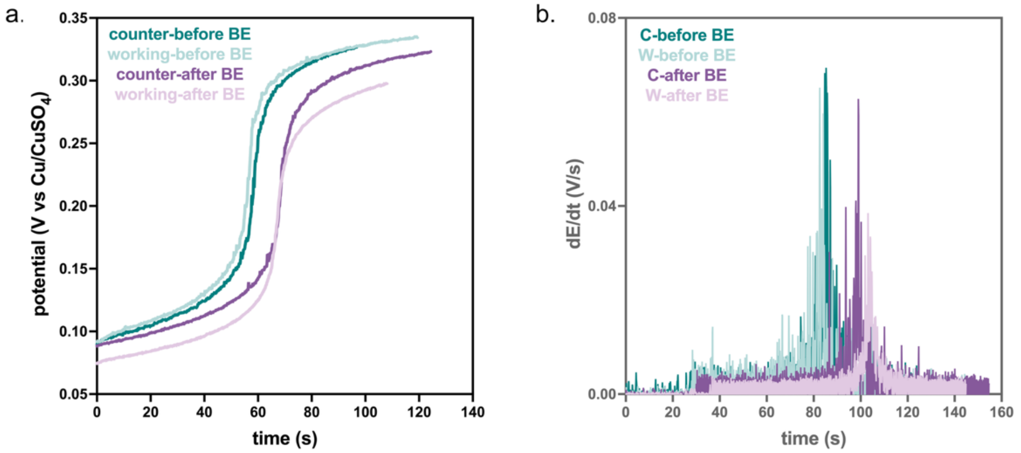

Figure S37. (a) Potentiometric titration plots of solutions from the counter and working side with the addition of $AgNO_3$. (b) Midpoint slopes (dE/dt) on the titration curve.

VI. Understanding DEHP loss

Section VI.A. Crossover studies to determine loss of DEHP

A stock solution of the supporting electrolyte was prepared by adding DMF (12.0 mL) to a vial containing NBu_4Cl (834 mg, 3.00 mmol) using a pipette. Subsequently, 6.00 mL of this supporting electrolyte solution was transferred via pipette to a separate vial containing PVC_{37k} (75.0 mg, 1.20 mmol (repeat unit), 1.00 equiv. (repeat unit)) to prepare the PVC stock solution. Subsequently, 5.50 mL of this PVC stock solution was transferred using a pipette to a separate vial containing DEHP (107 mg, 0.275 mmol, 0.250 equiv.) to prepare the working side solution stock. Similarly, 5.50 mL of the supporting electrolyte solution was added by a pipette to a vial with phenetole (134 mg, 1.10 mmol, 1.00 equiv.) to prepare the counter side stock solution.

Next, 5.00 mL of the working side solution was dispensed using a pipette into the working compartment of an H-cell. An additional 0.200 mL aliquot from the remaining working side stock was reserved for ^1H NMR analysis to determine DEHP concentration at 0 min. Simultaneously, 5.00 mL of the counter side solution was transferred to the counter compartment of the H-cell. No electrodes were used in this analysis. Both working and counter side of the H-cell were equipped with a Teflon-coated magnetic stir bar and the solutions were stirred at 950 rpm at room temperature for 7 h. Subsequently, a 0.200 mL aliquot from the working compartment was collected for ^1H NMR at 7 h to quantify DEHP crossover. ^1H NMR spectra from the working side at 0 min and at 7 h were analyzed to assess DEHP loss in the working side resulting from crossover.

Table S21. DEHP loss analysis via crossover calculated by ^1H NMR spectroscopy (see calculation in Section II.B).

peak area of DEHP at 7.72 ppm		peak area of TMB at 6.14 ppm		[DEHP] before BE	[DEHP] after BE	DEHP loss (%)
before BE	after BE	before BE	after BE			
190	171	138	142	41.4	36.2	12.6

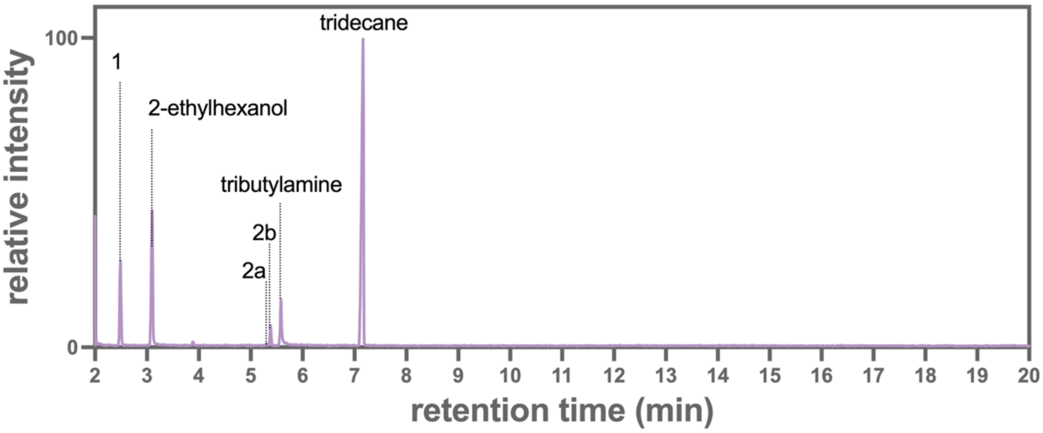

Figure S38. GC-MS chromatogram of working side after electrolysis according to Electrolysis Procedure D, using DEHP in working side and phenetole in counter side, showing formation of 2-ethylhexanol and tributylamine during electrolysis upon decomposition of DEHP and NBu_4^+, respectively.

Section VI.B. GCPL electrolysis of PVC_{37k} with phthalic anhydride or 2-ethylhexanol as a redox mediator

PVC_{37k} with phthalic anhydride: A stock solution of the supporting electrolyte was prepared by adding DMF (12.0 mL) to a vial containing NBu_4Cl (834 mg, 3.00 mmol) using a pipette. Subsequently, 6.00 mL of this supporting electrolyte solution was transferred via pipette to a separate vial containing PVC_{37k} (75.0 mg, 1.20 mmol (repeat unit), 1.00 equiv. (repeat unit)) to prepare the PVC stock solution. Subsequently, 5.50 mL of this PVC stock solution was transferred using a pipette to a separate vial containing phthalic anhydride (41.0 mg, 0.275 mmol, 0.250 equiv.) to prepare the working side solution stock. Similarly, 5.50 mL of the supporting electrolyte solution was added by a pipette to a vial with phenetole (134 mg, 1.10 mmol, 1.00 equiv.) to prepare the counter side stock solution.

Next, 5.00 mL of the working side solution was dispensed using a pipette into the working compartment of an H-cell containing RVC electrode and fritted Ag/Ag^+ as a working and reference electrode, respectively. Simultaneously, 5.00 mL of the counter side solution was transferred to the counter compartment of the H-cell containing RVC counter electrode. Aliquots were taken from the remaining working side stock for ^1H NMR analysis (refer Section II.B for phthalic anhydride loss calculation). Both working and counter side of the H-cell were equipped with a Teflon-coated magnetic stir bar and the reaction was stirred at 950 rpm at room temperature. The electrolysis was performed at constant current (–8 mA) with a voltage limit of –2.69 V (versus Ag/Ag^+). Once the reaction reached the voltage limit, electrolysis continued at this voltage until the total time of 7 h. Note that the total reaction time of 7 h consisted of the time at CP until the voltage limit was reached, followed by the remaining time at the constant-voltage. After the

electrolysis, aliquots were taken for ^{1}H NMR spectroscopic analysis (refer Section II.B for phthalic anhydride loss calculation). After the electrolysis, dPVC was extracted (Section IV.A) and sent for CIC to determine % dechlorination of PVC.

PVC_{37k} with 2-ethylhexanol: A stock solution of the supporting electrolyte was prepared by adding DMF (12.0 mL) to a vial containing NBu_4Cl (834 mg, 3.00 mmol) using a pipette. Subsequently, 6.00 mL of this supporting electrolyte solution was transferred via pipette to a separate vial containing PVC_{37k} (75.0 mg, 1.20 mmol (repeat unit), 1.00 equiv. (repeat unit)) to prepare the PVC stock solution. Subsequently, 5.50 mL of this PVC stock solution was transferred via pipette to a separate vial containing 2-ethylhexanol (36.0 mg, 0.275 mmol, 0.250 equiv.) to prepare the working side solution stock. Similarly, 5.50 mL of the supporting electrolyte solution was added by a pipette to a vial with phenetole (134 mg, 1.10 mmol, 1.00 equiv.) to prepare the counter side stock solution.

Next, 5.00 mL of the working side solution was dispensed using a pipette into the working compartment of an H-cell containing RVC electrode and fritted Ag/Ag^+ as a working and reference electrode, respectively. Simultaneously, 5.00 mL of the counter side solution was transferred to the counter compartment of the H-cell containing RVC counter electrode. Both working and counter side of the H-cell were equipped with a Teflon-coated magnetic stir bar and the reaction was stirred at 950 rpm at room temperature. The electrolysis was performed at constant current (–8 mA) with a voltage limit of –2.69 V (versus Ag/Ag^+). Once the reaction reached the voltage limit, electrolysis continued at this voltage until the total time of 7 h. Note that the total reaction time of 7 h consisted of the time at CP until the voltage limit was reached, followed by the remaining time at the constant-voltage. After the electrolysis, dPVC was extracted (Section IV.A) and sent for CIC to determine % dechlorination of PVC.

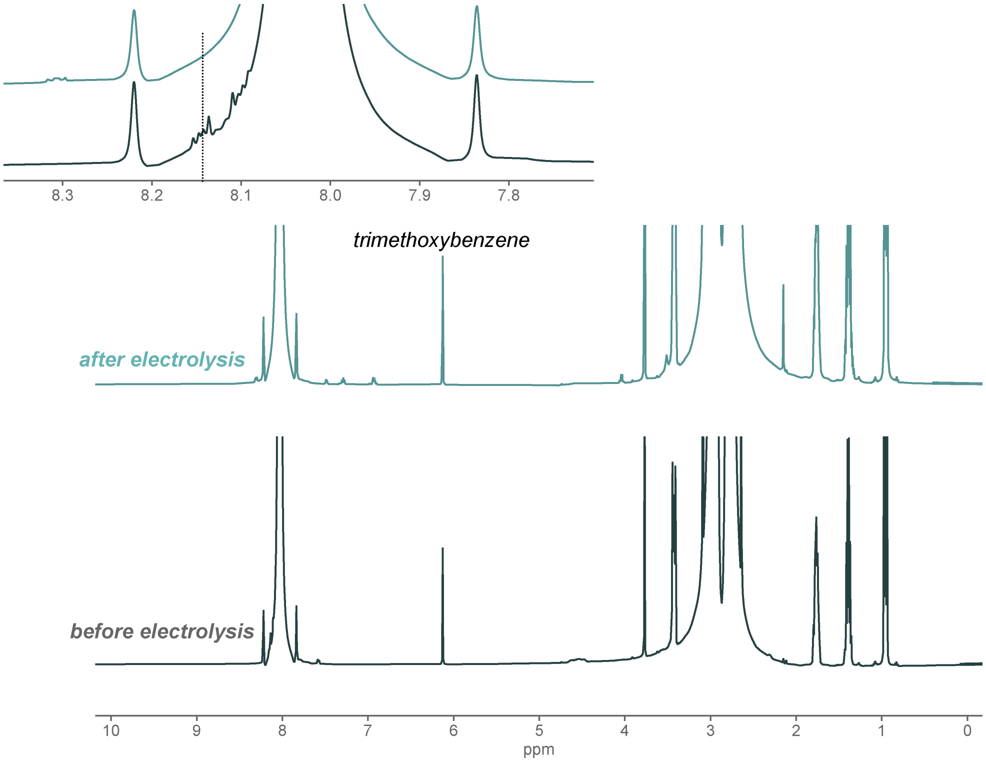

Figure S39. ^{1}H NMR spectroscopy of working side solution both before and after the electrolysis of phthalic anhydride and PVC_{37k} using the procedure described above. The absence of the characteristic phthalic anhydride peak at ~8.15 ppm in the after-electrolysis spectrum indicates the decomposition of phthalic anhydride during electrolysis.

Table S22. Combustion ion chromatography of dPVC samples extracted (Section IV.A) from the electrolysis with phthalic anhydride and 2-ethylhexanol as mediators. Percent dechlorination was calculated as shown in Section II.D.

experiment	mass %					dechlorination (%)
	C	H	N	Cl	rest	
0.25 equiv. phthalic anhydride + 1 equiv. PVC_{37k}	56.2	8.50	2.64	25.0	7.66	55.9
0.25 equiv. 2-ethylhexanol + 1 equiv. PVC_{37k}	38.9	4.88	0.000	56.1	0.12	1.04

Section VI.C. GCPL electrolysis of PVC_{37k} with DEHP to determine incorporation of DEHP or its decomposition products onto dPVC backbone

A stock solution of the supporting electrolyte was prepared by adding DMF (12.0 mL) to a vial containing NBu_4Cl (834 mg, 3.00 mmol) using a pipette. Subsequently, 6.00 mL of this supporting electrolyte solution was transferred via pipette to a separate vial containing PVC_{37k} (225 mg, 3.60 mmol (repeat unit), 6.00 equiv. (repeat unit)) to prepare the PVC stock solution. Then, 5.50 mL of this PVC stock solution was transferred via pipette to a separate vial containing DEHP (215 mg, 0.550 mmol, 1.00 equiv.) to prepare the working side solution stock. Similarly, 5.50 mL of the supporting electrolyte solution was added by a pipette to a vial with phenetole (134 mg, 1.10 mmol, 3.00 equiv.) to prepare the counter side stock solution.

Next, 5.00 mL of the working side solution was dispensed using a pipette into the working compartment of an H-cell containing RVC electrode and fritted Ag/Ag^+ as a working and reference electrode, respectively. Simultaneously, 5.00 mL of the counter side solution was transferred to the counter compartment of the H-cell containing RVC counter electrode. Both working and counter side of the H-cell were equipped with a Teflon-coated magnetic stir bar and the reaction was stirred at 950 rpm at room temperature. The electrolysis was performed for 2 h at constant current (–8 mA) with a voltage limit of –2.69 V (versus Ag/Ag^+). After the electrolysis, dPVC was extracted (Section IV.A) and sent for CIC to determine % dechlorination of PVC.

Table S23. Combustion ion chromatography of dPVC samples extracted (Section IV.A) from the electrolysis with DEHP and PVC_{37k}, according to the procedure in Section VI.C.

experiment	mass %					dechlorination (%)
	C	H	N	Cl	rest	
1 equiv. DEHP + 6 equiv. PVC_{37k}	48.2	6.19	0.000	44.9	0.710	20.8

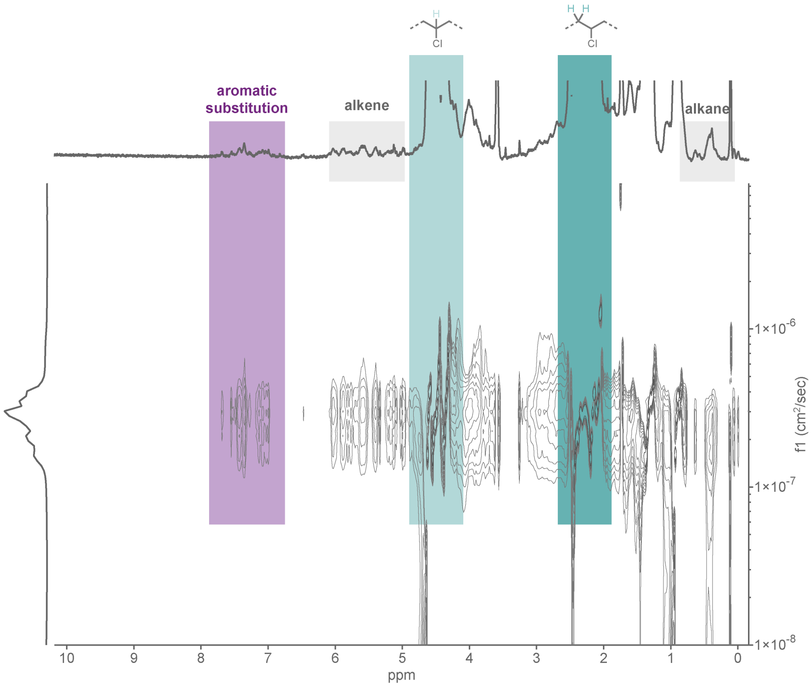

Figure S40. DOSY ^{1}H NMR spectrum of dPVC-20% suggest aromatic, alkene, and alkane substitution on the polymer backbone as they have similar diffusion coefficient compared to the characteristic PVC peaks at ~4.50 and ~2.50 ppm.

Table S24. SEC molar mass and dispersity for PVC_{37k} and dPVC-20% from the RI and UV detectors.

PVC identity	M_n (g/mol)	M_w (g/mol)	dispersity, *Đ*
PVC_{37k} (RI)	37,410 (trial 1)<br>37,840 (trial 2)	70,380 (trial 1)<br>69,990 (trial 2)	1.88 (trial 1)<br>1.85 (trial 2)
dPVC-20% (RI)	36,200 (trial 1)<br>35,480 (trial 2)	91,770 (trial 1)<br>90,390 (trial 2)	2.53 (trial 1)<br>2.55 (trial 2)
dPVC-20% (UV)	33,710 (trial 1)<br>34,610 (trial 2)	107,560 (trial 1)<br>98,530 (trial 2)	3.19 (trial 1)<br>2.85 (trial 2)

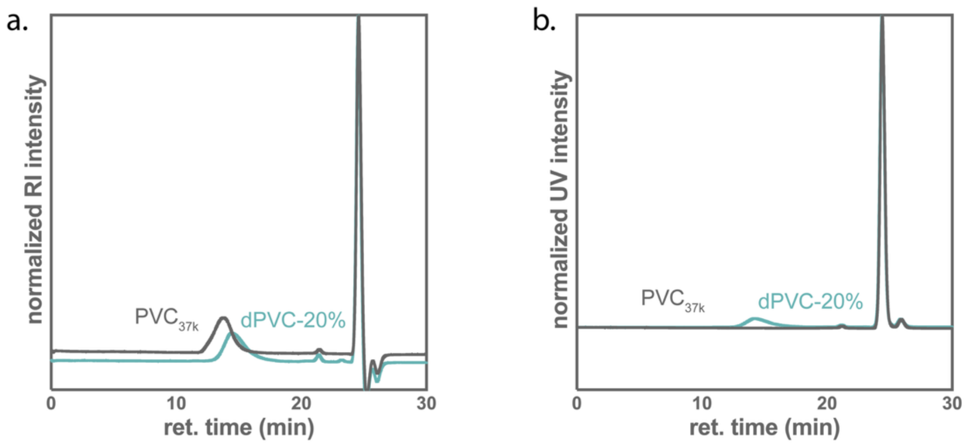

Figure S41. SEC RI (a) and UV (b) chromatograms of PVC_{37k} and dPVC-20%.

VII. Applicability to other plasticizers

Electrolysis Procedure E: (GCPL electrolysis, plasticizer and PVC, divided cell, 5.00 mL scale, NBu_4Cl as supporting electrolyte, voltage limit: –2.80 V versus Ag/Ag^+)

A stock solution of the supporting electrolyte was prepared by adding DMF (12.0 mL) to a vial containing NBu_4Cl (834 mg, 3.00 mmol) using a pipette. Subsequently, 6.00 mL of this supporting electrolyte solution was transferred via pipette to a separate vial containing PVC_{37k} (75.0 mg, 1.20 mmol (repeat unit), 1.00 equiv. (repeat unit)) to prepare the PVC stock solution. Then, 5.50 mL of this PVC stock solution was transferred via pipette to a separate vial containing respective plasticizer (0.550 mmol, 0.500 equiv.) to prepare the working side solution stock. Similarly, 5.50 mL of the supporting electrolyte solution was added by a pipette to a vial with phenetole (134 mg, 1.10 mmol, 1.00 equiv.) to prepare the counter side stock solution.

Next, 5.00 mL of the working side solution was dispensed using a pipette into the working compartment of an H-cell containing RVC electrode and fritted Ag/Ag^+ as a working and reference electrode, respectively. Simultaneously, 5.00 mL of the counter side solution was transferred to the counter compartment of the H-cell containing RVC counter electrode. Aliquots were taken from the remaining working and counter side stocks for ^1H NMR analysis (related to plasticizer loss analysis, Section II.B) and GC-MS analysis (related to phenetole chlorination, Section II.C), before electrolysis. Both working and counter side of the H-cell were equipped with a Teflon-coated magnetic stir bar and the reaction was stirred at 950 rpm at room temperature. The electrolysis was performed at constant current (–8 mA) with a voltage limit of –2.80 V (versus Ag/Ag^+). Once the reaction reaches the voltage limit, electrolysis continued at this voltage until the total time of 7 h. Note that the total reaction time of 7 h consisted of the time at CP until the voltage limit was reached, followed by the remaining time at the constant-voltage. At 7 h the current fell within the range of –1.2 to –1.5 mA, and the reaction was manually stopped. After the electrolysis, dPVC was extracted (Section IV.A) and sent for CIC to determine % dechlorination of PVC.

Note: The plasticizer and PVC source (PVC_{37k} and PVC_{122k}) vary depending on the reaction. For PVC_{122k}, the supporting electrolyte solution was added to the vial containing PVC_{122k} and stirred at room temperature for ~60 min, resulting in a suspension of swollen PVC.

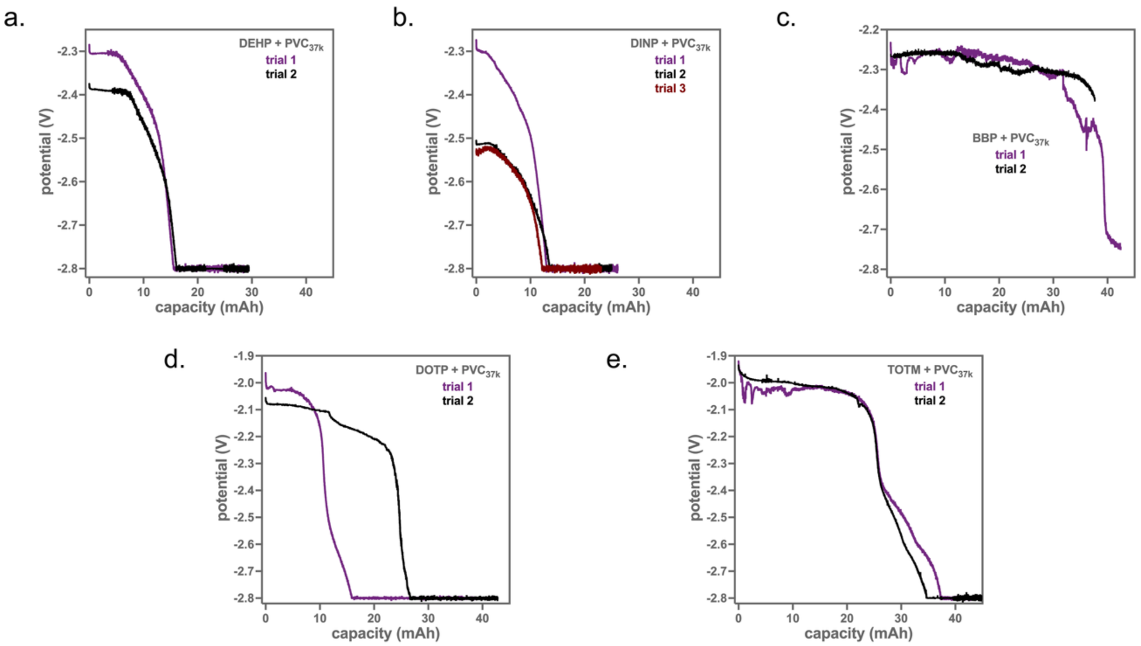

Figure S42. Potential versus capacity plots from GCPL electrolysis per Electrolysis Procedure E (current: –8 mA and voltage limit: –2.8 V versus Ag/Ag^{+}, 7 h); (a) DEHP and PVC_{37k}, (b) DINP and PVC_{37k}, (c) BBP and PVC_{37k},[1] (d) DOTP and PVC_{37k}, and (e) TOTM and PVC_{37k}. [1]*During the experiment with BBP and PVC_{37k}, solution from the counter side migrated to the working side, likely due to osmosis (Figure S43). Consequently, the volume in the counter side become too low for the electrode to remain adequately immersed, causing the potentiostat to automatically stop the reaction after ~5 h.*

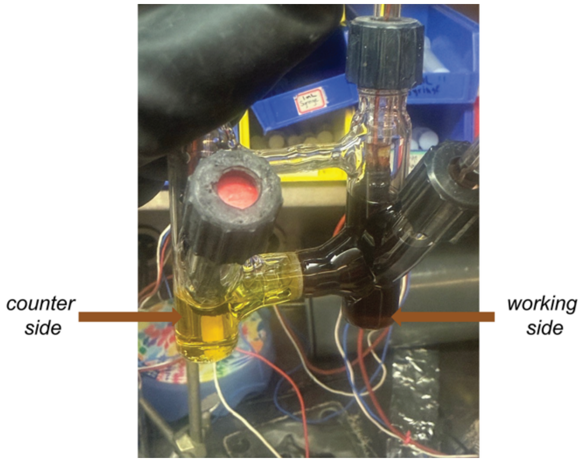

Figure S43. Photo of H-cell after electrolysis of BBP with PVC_{37k}, depicting crossover of counter side solution to the working side.

Table S25. Combustion ion chromatography of dPVC samples extracted from electrolysis as per Electrolysis Procedure E.

experiment	mass %					dechlorination (%)
	C	H	N	Cl	rest	
0.5 equiv. DEHP + 1 equiv. PVC_{37k} trial 1	74.0	9.38	0.180	4.92	11.5	91.3
0.5 equiv. DEHP + 1 equiv. PVC_{37k} trial 2	71.3	9.31	0.580	11.2	7.61	80.2
0.5 equiv. DINP + 1 equiv. PVC_{37k} Trial 1	74.3	9.47	0.180	7.31	8.74	87.1
0.5 equiv. DINP + 1 equiv. PVC_{37k} Trial 2	72.0	9.32	0.270	6.40	12.0	88.7
0.5 equiv. BBP + 1 equiv. PVC_{37k} trial 1	71.3	9.78	2.19	4.52	12.2	92.0
0.5 equiv. BBP + 1 equiv. PVC_{37k} trial 2	73.0	4.84	3.50	6.47	12.2	88.6
0.5 equiv. DOTP + 1 equiv. PVC_{37k} trial 1	68.3	9.84	1.36	9.38	11.1	83.4
0.5 equiv. DOTP + 1 equiv. PVC_{37k} trial 2	75.1	9.90	0.85	2.62	11.5	95.4
0.5 equiv. TOTM + 1 equiv. PVC_{37k} trial 1	70.4	9.38	0.940	2.00	17.3	96.5
0.5 equiv. TOTM + 1 equiv. PVC_{37k} trial 2	70.3	10.4	1.74	2.60	15.0	95.4
0.5 equiv. TOTM + 1 equiv. PVC_{122k}	67.1	10.8	2.15	9.13	10.8	83.9

Table S26. Plasticizer loss analysis calculated by ^{1}H NMR spectroscopy (see calculation in Section II.B). Electrolysis was performed as per Electrolysis Procedure E.

plasticizer	peak area of plasticizer		peak area of TMB at 6.14 ppm		[plasticizer] before BE	[plasticizer] after BE	plasticizer loss (%)
	before BE	after BE	before BE	after BE			
DEHP – trial 1	137	52.4	124	125	33.7	12.5	62.9
DEHP – trial 2	158	75.8	124	121	38.2	18.8	50.8
DINP – trial 1	134	59.5	119	118	33.9	15.1	55.4
DINP – trial 2	184	105	152	150	36.3	21.0	42.1
BBP – trial 1	136	2.63	126	134	32.3	0.600	98.1
BBP – trial 2	136	0	126	119	32.3	0	100
DOTP – trial 1	290	70.9	134	135	32.4	7.88	75.7
DOTP – trial 2	280	21.9	118	122	35.6	2.70	92.4
TOTM – trial 1	67.7	9.35	127	129	31.9	4.35	86.4
TOTM – trial 2	67.7	10.0	127	132	31.9	4.54	86.6

Table S27. Raw data extracted from representative GC-MS chromatograms recorded after a GCPL electrolysis of TOTM with PVC_{37k} and PVC_{122k} as per Electrolysis Procedure E. The capacity accessed during electrolysis is included with each experiment to determine the yield of **2**. Yield of **2** was calculated according to the representative calculations shown in Section II.C and Section II.D.

experiment and capacity accessed	compounds	GC-MS peak area			yield of **2** (%)			
		c-0 min	c-7 h	w-7 h	GC-MS yield (**assumption A**)	Faradaic yield	GC-MS yield (**assumption B**)	Faradaic yield
0.5 equiv. TOTM + 1 equiv. PVC_{37k} (44 mAh)	**1**	16303185	3688377	2478536	76	93	69	84
	**2a**	0	998755	127685				
	**2b**	0	9998209	1412987				
	tridecane	19553615	20100666	20471992				
0.5 equiv. TOTM + 1 equiv. PVC_{122k} (36 mAh)	**1**	16303185	4430495	2339501	64	95	66	98
	**2a**	0	1127992	90828				
	**2b**	0	11359780	896970				
	tridecane	19553615	25560367	23203904				

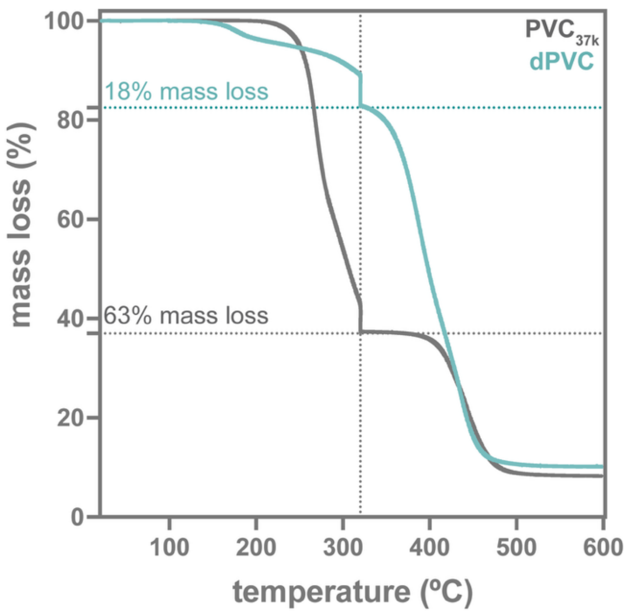

Figure S44. Plot of mass loss versus temperature obtained for PVC_{37k} and dPVC extracted from the reaction of GCPL electrolysis, as per Electrolysis Procedure E, using TOTM and PVC_{37k} in the working side and phenetole in the counter side.

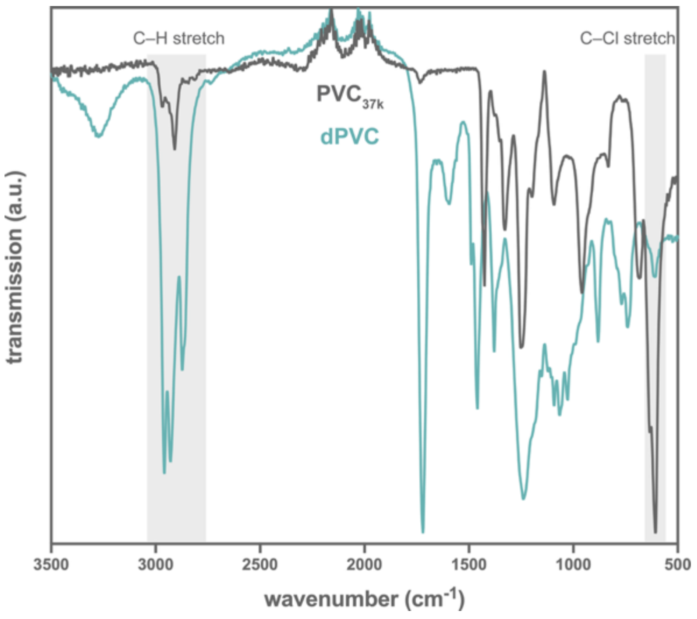

Figure S45. FT-IR spectra obtained for PVC_{37k} and dPVC extracted from the reaction of GCPL electrolysis, as per Electrolysis Procedure E, using TOTM and PVC_{37k} in the working side and phenetole in the counter side.

VIII. Electrolysis with real-world PVC plastics

Section VIII.A. Extraction of plasticizer from flexible tubing

Flexible tubing (~2.00 g) manually cut into smaller pieces and then stirred in THF (~50.0 mL) at 60 ºC for 30 min. Upon dissolution, the solution was poured into cold methanol (~200 mL) to precipitate the PVC. The polymer was filtered, and the filtrate was concentrated under reduced pressure. The dissolution and filtration was repeated on the filtered polymer two more times. During the final recovery, the precipitated PVC was then filtered, washed with methanol (~100 mL), and dried under high vacuum overnight (~15 h) to obtain white, flaky PVC_{tubing} (~1.15 g). The combined filtrates were concentrated under reduced pressure and dried overnight (~15 h) under vacuum to remove any residual solvent. A colorless oil was obtained (0.567 g, ~28.3 weight %). GC-MS analysis matched with the DEHP standard (Figure S42).

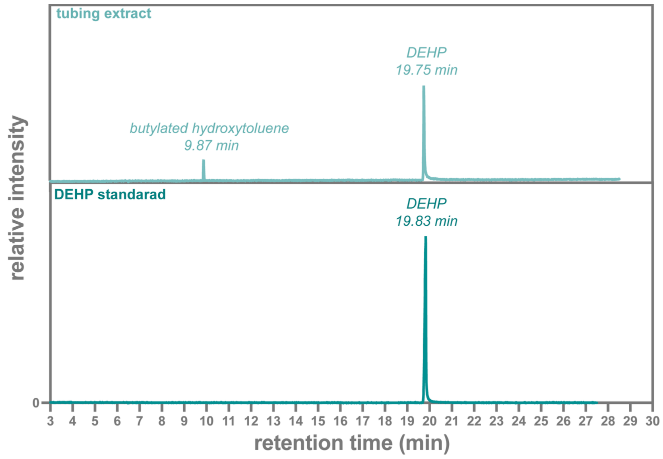

Figure S46. Stacked GC-MS chromatograms of DEHP standard (Sigma-Aldrich) and liquid extracted from the tubing sample. Butylated hydroxytoluene, a common additive used with flexible tubing,[5] was also observed.

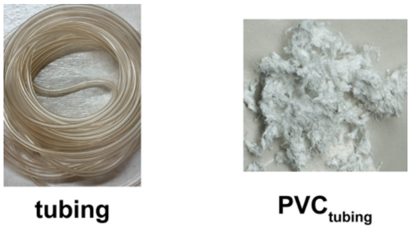

Figure S47. Photos of flexible tubing and extracted PVC from the tubing (PVC_{tubing}).

Section VIII.B. Extraction of PVC from rigid pipe

Rigid pipe (~2.00 g) was manually cut into smaller pieces and then stirred in THF (~60.0 mL) at 60 ºC for 60 min. A white cloudy mixture was observed. While still warm, the cloudy suspension was filtered through a Whatman® GF/D 2.7 µm glass fiber filter paper before being further filtered through Chromafil GF/RC 1.0/0.20 µm syringe filters to remove insoluble fillers, yielding a clear solution. The solution was then poured into cold methanol (~300 mL) to precipitate the PVC. The precipitated PVC was filtered, washed with methanol (~100 mL), and dried under vacuum overnight (~15 h) to obtain white, fluffy PVC_{pipe} (~1.45 g).

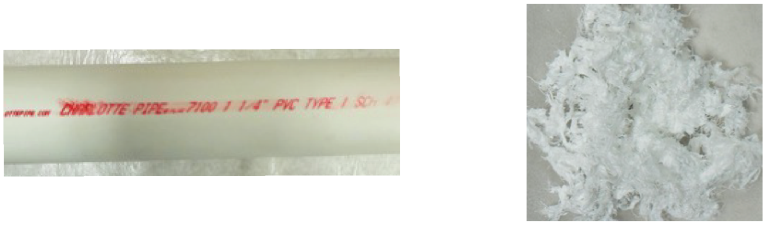

pipe **PVC_{pipe}**

Figure S48. Photos of pipe and extracted PVC from it (PVC_{pipe}).

Table S28. SEC molar mass and dispersity for PVC_{tubing} and PVC_{pipe}.

PVC identity	M_n (g/mol)	M_w (g/mol)	dispersity, *Đ*
PVC_{tubing}	102,220 (trial 1)<br>101,900 (trial 2)	251,730 (trial 1)<br>252,880 (trial 2)	2.46 (trial 1)<br>2.48 (trial 2)
PVC_{pipe}	78,280 (trial 1)<br>75,090 (trial 2)	172,760 (trial 1)<br>165,990 (trial 2)	2.21 (trial 1)<br>2.21 (trial 2)

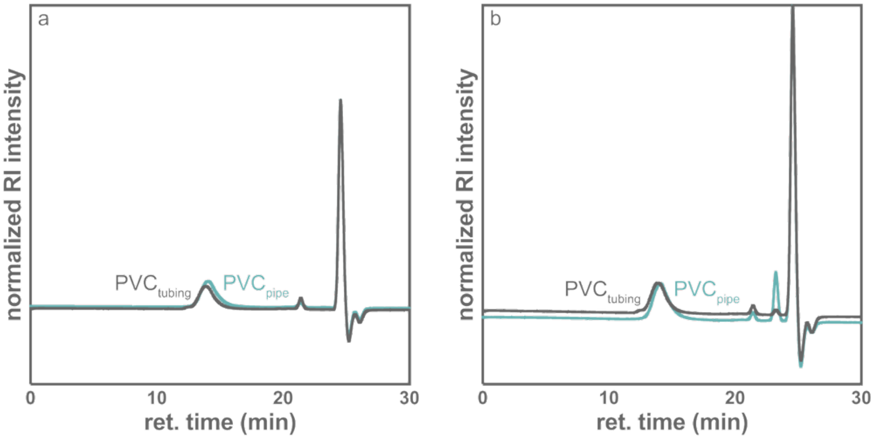

Figure S49. SEC RI chromatograms for trial 1 (a) and trial 2 (b) of PVC_{tubing} and PVC_{pipe}.

Electrolysis Procedure F: (GCPL electrolysis with tubing, divided cell, 5.00 mL scale, NBu_4Cl as supporting electrolyte, voltage limit: –2.80 V versus Ag/Ag^+)

A stock solution of the supporting electrolyte was prepared by adding DMF (12.0 mL) to a vial containing NBu_4Cl (834 mg, 3.00 mmol) using a pipette. Subsequently, 6.00 mL of this supporting electrolyte solution was transferred via pipette to a separate vial containing tubing (105 mg, cut into smaller pieces) to prepare the working stock solution. The mixture was stirred at 50 °C for 30 min to obtain a clear solution, which was cooled back to room temperature. Similarly, 5.50 mL of the supporting electrolyte solution was added by a pipette to a vial with phenetole (134 mg, 1.10 mmol) to prepare the counter side stock solution.

Next, 5.00 mL of the working side solution was dispensed using a pipette into the working compartment of an H-cell containing RVC electrode and fritted Ag/Ag^+ as a working and reference electrode, respectively. Simultaneously, 5.00 mL of the counter side solution was transferred to the counter compartment of the H-cell containing RVC counter electrode. Aliquots were taken from the remaining working and counter side stocks for ^1H NMR analysis (related to DEHP loss analysis, Section II.B) and GC-MS analysis (related to phenetole chlorination, Section II.C), before electrolysis. Both working and counter side of the H-cell were equipped with a Teflon-coated magnetic stir bar and the reaction was stirred at 950 rpm at room temperature. The electrolysis was performed at constant current (–8 mA) with a voltage limit of –2.80 V (versus Ag/Ag^+). Once the reaction reached the voltage limit, electrolysis continued at this voltage until the total time of 7 h. Note that the total reaction time of 7 h consisted of the time at CP until the voltage limit was reached, followed by the remaining time at the constant-voltage. After the electrolysis, aliquots were taken for ^1H NMR (for DEHP loss analysis, Section II.B) and GC-MS analysis for determining the yield of Cl-phenetole (Section II.C). After the electrolysis, dPVC was extracted (Section IV.A) and sent for CIC to determine % dechlorination of PVC.

Given the ~28.3 wt.% of DEHP in the tubing, 105 mg of tubing should include ~75.0 mg of PVC. This same mass of PVC was used in the electrolysis with neat PVC_{37k}. Based on these calculations, the DEHP concentration in 105 mg of tubing is estimated to be 0.0137 M (with a theoretical capacity of ~1.84 mAh).

Electrolysis Procedure G: (GCPL electrolysis with rigid pipe and TOTM, divided cell, 5.00 mL scale, NBu_4Cl as supporting electrolyte, voltage limit: –2.80 V versus Ag/Ag^+)

A stock solution of the supporting electrolyte was prepared by adding DMF (12.0 mL) to a vial containing NBu_4Cl (834 mg, 3.00 mmol) using a pipette. Subsequently, 6.00 mL of this supporting electrolyte solution was transferred via pipette to a separate vial containing pipe (100 mg, cut into smaller pieces). The mixture was stirred at 50 °C for 60 min to obtain a cloudy solution, which was cooled back to room temperature. Subsequently, 5.50 mL of this cloudy solution was

transferred via pipette to a separate vial containing TOTM (301 mg, 0.550 mmol) to prepare the working side solution stock. Similarly, 5.50 mL of the supporting electrolyte solution was added by a pipette to a vial with phenetole (134 mg, 1.10 mmol) to prepare the counter side stock solution.

Next, 5.00 mL of the working side solution was dispensed using a pipette into the working compartment of an H-cell containing RVC electrode and fritted Ag/Ag^+ as a working and reference electrode, respectively. Simultaneously, 5.00 mL of the counter side solution was transferred to the counter compartment of the H-cell containing RVC counter electrode. Aliquots were taken from the remaining working and counter side stocks for ^1H NMR spectroscopic analysis (related to TOTM loss analysis, Section II.B) and GC-MS analysis (related to phenetole chlorination, Section II.C), before electrolysis. Both working and counter side of the H-cell were equipped with a Teflon-coated magnetic stir bar and the reaction was stirred at 950 rpm at room temperature. The electrolysis was performed at constant current (–8.00 mA) with a voltage limit of –2.80 V (versus Ag/Ag^+). Once the reaction reaches the voltage limit, electrolysis continues at this voltage until the total time of 7 h. The total reaction time of 7 h consist of the time at CP until the voltage limit was reached, followed by the remaining time at the constant-voltage. After the electrolysis, aliquots were taken for ^1H NMR (for TOTM loss analysis, Section II.B) and GC-MS analysis for determining the yield of Cl-phenetole (Section II.C). After the electrolysis, dPVC was extracted (Section IV.A) and sent for CIC to determine % dechlorination of PVC.

Electrolysis Procedure H: (GCPL electrolysis with PVC_{pipe} or PVC_{tubing} and TOTM, divided cell, 5.00 mL scale, NBu_4Cl as supporting electrolyte, voltage limit: –2.80 V versus Ag/Ag^+)

A stock solution of the supporting electrolyte was prepared by adding DMF (12.0 mL) to a vial containing NBu_4Cl (834 mg, 3.00 mmol) using a pipette. 6.00 mL of this supporting electrolyte solution was transferred via pipette to a separate vial containing PVC_{pipe} or PVC_{tubing} (75.0 mg, 1.10 mmol (repeat unit), 1.00 equiv. (repeat unit)). The mixture was stirred at 50 °C for 30 min to obtain a clear solution, which was cooled back to room temperature. Subsequently, 5.50 mL of this clear solution was transferred via pipette to a separate vial containing TOTM (301 mg, 0.550 mmol, 0.500 equiv.) to prepare the working side solution stock. Similarly, 5.50 mL of the supporting electrolyte solution was added by a pipette to a vial with phenetole (134 mg, 1.10 mmol) to prepare the counter side stock solution.

Next, 5.00 mL of the working side solution was dispensed using a pipette into the working compartment of an H-cell containing RVC electrode and fritted Ag/Ag^+ as a working and reference electrode, respectively. Simultaneously, 5.00 mL of the counter side solution was transferred to the counter compartment of the H-cell containing RVC counter electrode. Aliquots were taken from the remaining working and counter side stocks for ^1H NMR analysis (related to TOTM loss analysis, Section II.B) and GC-MS analysis (related to phenetole chlorination, Section II.C), before

electrolysis. Both working and counter side of the H-cell were equipped with a Teflon-coated magnetic stir bar and the reaction was stirred at 950 rpm at room temperature. The electrolysis was performed at constant current (–8.00 mA) with a voltage limit of –2.80 V (versus Ag/Ag^{+}). Once the reaction reached the voltage limit, electrolysis continued at this voltage until the total time of 7 h. The total reaction time of 7 h consist of the time at CP until the voltage limit was reached, followed by the remaining time at the constant-voltage. After the electrolysis, aliquots were taken for ^{1}H NMR (for TOTM loss analysis, Section II.B) and GC-MS analysis for determining the yield of Cl-phenetole (Section II.C). After the electrolysis, dPVC was extracted (Section IV.A) and sent for CIC to determine % dechlorination of PVC.

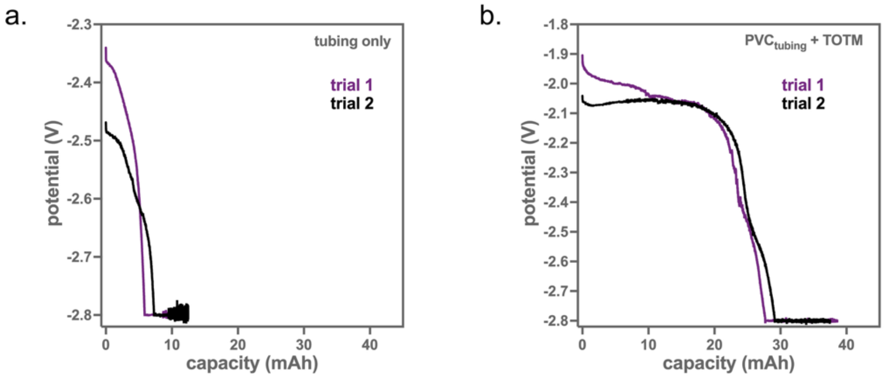

Figure S50. Potential versus capacity of GCPL electrolysis with (a) tubing, as per Electrolysis Procedure F, and (b) with PVC_{tubing} and TOTM, as per Electrolysis Procedure H, both trials 1 and 2 are included.

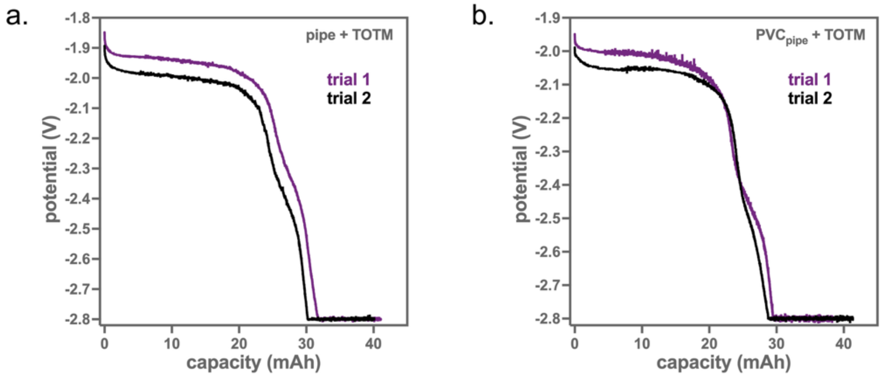

Figure S51. Potential versus capacity of GCPL electrolysis with (a) pipe and TOTM, as per Electrolysis Procedure G, and (b) with PVC_{pipe} and TOTM, as per Electrolysis Procedure H. Both the trials 1 and 2 were included.

Table S29. Combustion ion chromatography of dPVC samples extracted from electrolysis as per Electrolysis Procedure F[a], G[b], and H[c].

experiment	mass %					dechlorination (%)
	C	H	N	Cl	rest	
[a]tubing	59.5	7.36	0.000	27.3	5.83	16.5
[b]TOTM + pipe trial 1	66.6	9.62	0.790	7.94	15.1	85.7
[b]TOTM + pipe trial 2	68.0	9.02	0.34	7.50	15.1	86.8
[c]0.5 equiv. TOTM + 1 equiv. PVC_{tubing} trial 1	70.1	9.70	0.770	2.83	16.6	95.0
[c]0.5 equiv. TOTM + 1 equiv. PVC_{tubing} trial 2	72.5	9.76	1.45	4.31	12.0	92.4
[c]0.5 equiv. TOTM + 1 equiv. PVC_{pipe} trial 1	67.6	9.68	0.730	9.49	12.4	83.2
[c]0.5 equiv. TOTM + 1 equiv. PVC_{pipe} trial 2	66.0	9.15	0.870	8.91	15.1	84.3

Table S30. TOTM loss analysis calculated by ^{1}H NMR spectroscopy (see calculation in Section II.B). Electrolysis was performed as per Electrolysis Procedure G[a], and H[b].

experiment	peak area of TOTM at 8.39 ppm		peak area of TMB at 6.14 ppm		[TOTM] before BE	[TOTM] after BE	TOTM loss (%)
	before BE	after BE	before BE	after BE			
[a]TOTM + pipe trial 1	67.7	9.24	127	139	31.9	3.99	87.5
[a]TOTM + pipe trial 2	67.7	7.31	127	135	31.9	3.25	89.8
[b]0.5 equiv. TOTM + 1 equiv. PVC_{pipe} trial 1	67.7	8.92	127	159	31.9	3.36	89.5
[b]0.5 equiv. TOTM + 1 equiv. PVC_{pipe} trial 2	67.7	7.49	127	135	31.9	3.33	89.6
[b]0.5 equiv. TOTM + 1 equiv. PVC_{tubing} trial 1	74.3	7.98	122	140	36.5	3.41	90.6
[b]0.5 equiv. TOTM + 1 equiv. PVC_{tubing} trial 2	82.8	10.8	132	130	37.6	4.98	86.7

Table S31. Raw data extracted from representative GC-MS chromatograms recorded after a GCPL electrolysis as per Electrolysis Procedure F[a], using tubing in working side and phenetole (**1**) in counter side; as per Electrolysis Procedure G[b], using pipe and TOTM in working side and **1** in counter side; Electrolysis Procedure H[c], using PVC_{tubing} and PVC_{pipe} with TOTM in working side and **1** in counter side. The capacity accessed during electrolysis is included with each experiment. Yield of **2** was calculated according to the representative calculations shown in Section II.C and Section II.D.

experiment and capacity accessed	compounds	GC-MS peak area			yield of **2** (%)			
		c-0 min	c-7 h	w-7 h	GC-MS yield (**assumption A**)	Faradaic yield	GC-MS yield (**assumption B**)	Faradaic yield
[c]0.5 equiv. TOTM + 1 equiv. PVC_{tubing} (38.6 mAh) trial 1	**1**	16303185	2277888	2050959	85	117	79	108
	**2a**	0	1263682	101453				
	**2b**	0	13428985	1082751				
	tridecane	19553615	23076154	23615863				
[c]0.5 equiv. TOTM + 1 equiv. PVC_{tubing} (37.5 mAh) trial 2	**1**	28064462	1223671	2510106	87	124	88	126
	**2a**	0	1741980	54504				
	**2b**	0	21800221	582840				
	tridecane	25264358	25354046	22771754				
[a]tubing (11.5 mAh)	**1**	19292480	15369256	1691006	26	121	25	116
	**2a**	0	471114	0				
	**2b**	0	4780908	143619				
	tridecane	21282550	23050627	21567850				
[c]0.5 equiv. TOTM + 1 equiv. PVC_{pipe} (38.3 mAh) trial 1	**1**	16303185	19553615	2372714	87	121	80	112
	**2a**	0	1357133	70933				
	**2b**	0	16238613	1020545				
	tridecane	19553615	26436038	1020545				
[c]0.5 equiv. TOTM + 1 equiv. PVC_{pipe} (41.4 mAh) trial 2	**1**	28064462	3774575	2274849	75	97.1	79	102
	**2a**	0	1685817	20501				
	**2b**	0	17538633	458332				
	tridecane	25264358	23946326	24680442				
[b]TOTM + pipe (41.1 mAh) trial 1	**1**	16303185	2187587	1166400	81	105	84	109
	**2a**	0	1245412	50392				
	**2b**	0	14527428	783868				
	tridecane	19553615	25208841	24727130				
[b]TOTM + pipe (40.1 mAh) trial 2	**1**	28064462	1212769	2555097	91	122	86	115
	**2a**	0	1715324	61871				
	**2b**	0	20963400	796752				
	tridecane	25264358	23890968	19199846				

IX. Scale-up studies with TOTM and PVC_{37k}

Electrolysis Procedure I: (GCPL electrolysis, TOTM and PVC_{37k}, divided cell, 25.0 mL working side and 20 mL counter side, NBu_4Cl as supporting electrolyte, voltage limit: –2.80 V versus Ag/Ag^+)

A stock solution of the supporting electrolyte was prepared by adding DMF (48.0 mL) to a vial containing NBu_4Cl (5340 mg, 19.2 mmol) using a pipette. Subsequently, 26.0 mL of this supporting electrolyte solution was transferred via pipette to a separate vial containing PVC_{37k} (500 mg, 8.01 mmol (repeat unit), 1.00 equiv. (repeat unit)) to prepare the PVC stock solution. Then, 25.5 mL of this PVC stock solution was transferred via pipette to a separate vial containing TOTM (2150 mg, 3.93 mmol, 0.500 equiv.) to prepare the working side solution stock. Similarly, 20.5 mL of the supporting electrolyte solution was added by a pipette to a vial with phenetole (770 mg, 6.31 mmol, 1.00 equiv.) to prepare the counter side stock solution.

Next, 25.0 mL of the working side solution was dispensed using a pipette into the working compartment of an H-cell (Figure S16) containing RVC electrode and fritted Ag/Ag^+ as a working and reference electrode, respectively. Simultaneously, 20.0 mL of the counter side solution was transferred to the counter compartment of the H-cell containing RVC counter electrode. Aliquots were taken from the remaining working and counter side stocks for ^1H NMR analysis (related to TOTM loss analysis, Section II.B) and GC-MS analysis (related to phenetole chlorination, Section II.C), before electrolysis. Both working and counter side of the H-cell were equipped with a Teflon-coated magnetic stir bar and the reaction was stirred at 500 rpm at room temperature. The electrolysis was performed at constant current (–8.00 mA) with a voltage limit of –2.80 V (versus Ag/Ag^+). After the electrolysis, aliquots were taken for ^1H NMR (for TOTM loss analysis, Section II.B) and GC-MS analysis for determining the yield of Cl-phenetole (Section II.C). After the electrolysis, dPVC was extracted (Section IV.A) and sent for CIC to determine % dechlorination of PVC.

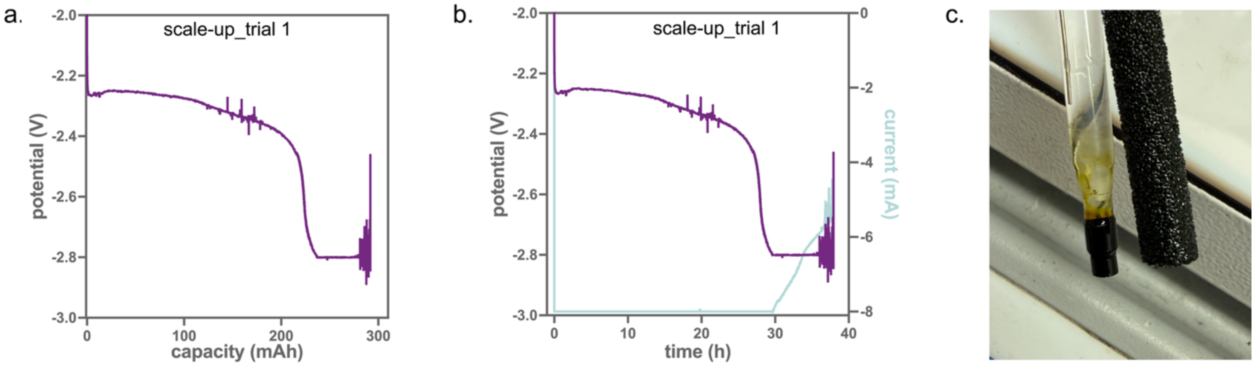

Figure S52. (a) Potential versus capacity plot, (b) potential versus time versus current plot of the GCPL electrolysis of TOTM and PVC_{37k} per Electrolysis Procedure I, (c) deposition of dPVC at the frit of the Ag/Ag^+ reference electrode (left). Reaction proceeded at constant current for ~30 h and the next ~8 h at the voltage limit. The electrolysis stopped at ~38 h due to control amplifier overload in the potentiostat, likely due to increased solution resistance and dPVC precipitation on the frit of the reference electrode. ~96% capacity was accessed (theoretical capacity: 302 mAh).

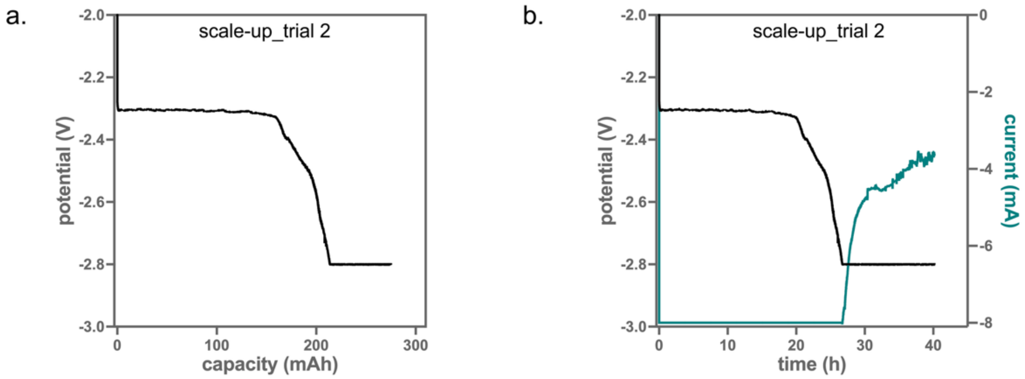

Figure S53. (a) Potential versus capacity plot, (b) potential versus time versus current plot of the GCPL electrolysis of TOTM and PVC_{37k} per Electrolysis Procedure I. Reaction proceeded at constant current (–8 mA) for ~28 h and the next ~12 h at the voltage limit (–2.80 V). The electrolysis stopped at ~40 h due to control amplifier overload in the potentiostat. ~91% capacity was accessed (theoretical capacity: 302 mAh).

Table S32. Combustion ion chromatography of dPVC samples extracted from electrolysis as per Electrolysis Procedure I.

experiment	mass %					dechlorination (%)
	C	H	N	Cl	rest	
TOTM + PVC_{37k} trial1	74.1	10.0	1.30	0.420	14.2	99.3
TOTM + PVC_{37k} Trial2	73.6	9.34	0.84	2.47	13.6	95.6

Table S33. TOTM loss analysis calculated by ^{1}H NMR spectroscopy (see calculation in Section II.B). Electrolysis was performed as per Electrolysis Procedure I.

experiment	peak area of TOTM at 8.39 ppm		peak area of TMB at 6.14 ppm		[TOTM] before BE	[TOTM] after BE	TOTM loss (%)
	before BE	after BE	before BE	after BE			
TOTM + PVC_{37k} trial 1	102	8.45	132	134	46.4	3.77	92.8
TOTM + PVC_{37k} trial 2	111	20.2	131	138	50.8	8.78	82.7

Table S34. Raw data extracted from representative GC-MS chromatograms recorded after a GCPL electrolysis of TOTM with PVC_{37k} as per Electrolysis Procedure I. The capacity accessed during electrolysis is included with trials. Yield of **2** was calculated according to the representative calculations shown in Section II.C and Section II.D.

experiment and capacity accessed	compounds	GC-MS peak area			yield of **2** (%)			
		c-0 min	c-after BE	w-after BE	GC-MS yield (**assumption A**)	Faradaic yield	GC-MS yield (**assumption B**)	Faradaic yield
TOTM + PVC_{37k} (292 mAh) trial 1	**1**	13958111	4448252	1367729	57	64	59	67
	**2a**	0	744252	14350				
	**2b**	0	6608295	218537				
	tridecane	15391144	16191163	17951065				
TOTM + PVC_{37k} (275 mAh) trial 2	**1**	39572188	10631056	3420792	58	70	57	68
	**2a**	0	1706480	82436				
	**2b**	0	17434531	754217				
	tridecane	26793694	24079634	24430498				

X. References

[1] D. E. Fagnani, D. Kim, S. I. Camarero, J. F. Alfaro, and A. J. McNeil, "Using waste poly(vinyl chloride) to synthesize chloroarenes by plasticizer-mediated electro(de)chlorination," *Nature Chemistry* 15 (2023): 222–229.

[2] J. Wu, K. G. Papanikolaou, F. Cheng, B. Addsion, A. A. Cuthbertson, M. Mavrikakis, and G. W. Huber, "Kinetic study of polyvinyl chloride pyrolysis with characterization of dehydrochlorinated PVC," *ACS Sustainable Chemistry & Engineering* 12 (2024): 7402–7413.

[3] A. V. Benedetti, C. S. Fugivara, M. Cilense, and T. Rabockai, "Reference electrodes in dimethylformamide," *Analytical Letters* 16 (1983): 1357–1370.

[4] B. J. Neyhouse, G. E. Cook, R. K. Jha, and A. J. McNeil, "Electrochemical generation of chlorine and hydrogen from waste poly(vinyl chloride)," *ACS Sustainable Chemistry & Engineering* 13 (2025): 13042–13050.

[5] A. A. Cuthbertson, C. Lincoln, J. Miscall, L. M. Stanley, A. K. Maurya, A. S. Asundi, C. J. Tassone, N. A. Rorrer, and G. T. Beckham, "Characterization of polymer properties and identification of additives in commercially available research plastics," *Green Chemistry* 26 (2024): 7067–7090.
